# Fibroblast growth factor receptor type 4 as a potential therapeutic target in clear cell renal cell carcinoma

**DOI:** 10.1186/s12885-023-10638-3

**Published:** 2023-02-20

**Authors:** Takafumi Narisawa, Sei Naito, Hiromi Ito, Osamu Ichiyanagi, Toshihiko Sakurai, Tomoyuki Kato, Norihiko Tsuchiya

**Affiliations:** Department of Urology, Yamagata University Faculty of Medicine, 2-2-2 Iida-nishi, Yamagata, 990-9585 Japan

**Keywords:** Clear cell renal cell carcinoma (ccRCC), FGFR4, BLU9931

## Abstract

**Background:**

Several clear cell renal cell carcinoma (ccRCC) cases harbour fibroblast growth factor receptor 4 (*FGFR4*) gene copy number (CN) gains. In this study, we investigated the functional contribution of *FGFR4* CN amplification in ccRCC.

**Methods:**

The correlation between *FGFR4* CN determined via real-time PCR and protein expression evaluated using western blotting and immunohistochemistry was assessed in ccRCC cell lines (A498, A704, and 769-P), a papillary RCC cell line (ACHN), and clinical ccRCC specimens. The effect of *FGFR4* inhibition on ccRCC cell proliferation and survival was assessed via either RNA interference or using the selective FGFR4 inhibitor BLU9931, followed by MTS assays, western blotting, and flow cytometry. To investigate whether FGFR4 is a potential therapeutic target, a xenograft mouse model was administered BLU9931.

**Results:**

60% of ccRCC surgical specimens harboured an *FGFR4* CN amplification. *FGFR4* CN was positively correlated with its protein expression. All ccRCC cell lines harboured *FGFR4* CN amplifications, whereas ACHN did not. FGFR4 silencing or inhibition attenuated intracellular signal transduction pathways, resulting in apoptosis and suppressed proliferation in ccRCC cell lines. BLU9931 suppressed tumours at a tolerable dose in the mouse model.

**Conclusion:**

FGFR4 contributes to ccRCC cell proliferation and survival following *FGFR4* amplification, making it a potential therapeutic target for ccRCC.

**Supplementary Information:**

The online version contains supplementary material available at 10.1186/s12885-023-10638-3.

## Background

Renal cell carcinoma (RCC) is the seventh most common cancer worldwide, and its incidence has been on the rise in many countries [1]. Approximately 50% of patients with RCC would have already developed metastasis at diagnosis or go on to develop a metastatic disease during their lifetime [2]. Targeted treatments, including VEGF receptor inhibitors and mTOR inhibitors, have been shown to improve the survival of patients with metastatic RCC [3, 4]. During recent years, the use of immuno-oncological drugs (I-O drugs) has improved the prognosis of RCC in clinical trials [5, 6]. As a characteristic clinical trend, targeted treatments rarely exhibit long-term efficacy [7]. In contrast, immune checkpoint inhibitors provide long-term disease control, but only in some patients. CheckMate 214, a phase III trial on the efficacy of immune checkpoint inhibitor combination therapies, revealed that the long-term progression-free survival rate (> 2 years) was limited to 35% [8]. Recently, combination treatments of I-O drug and VEGF inhibitor, such as avelumab + axitinib and pembrolizumab + axitinib, have been developed [9, 10]. Moreover, combinations of I-O drug and multi-kinase inhibitor that target concomitant pathways of VEGF inhibition have been developed, for example, nivolumab + cabozantinib and pembrolizumab + lenvatinib [11, 12].

However, these novel treatment strategies are highly intensive as first-line treatments. There are several problems such as patient selectivity, highly severe adverse events, and a lack of biomarkers of patient benefits. Hence, the development of novel treatments remains a necessity.

A pan-fibroblast growth factor receptor (FGFR) inhibitor, lenvatinib, has been developed and used clinically [12, 13]. The results of the CLEAR trial have shown an extremely high tumour reduction rate (objective response 71.0%, complete response 16.1%) [12]. On the contrary, the basic mechanism through which FGFR inhibition directly suppresses RCC is still not clear.

Clear cell renal cell carcinoma (ccRCC) is the most common histological RCC subtype, accounting for 70–75% of all cases [2]. Approximately 90% of ccRCCs harbour genetic alterations in von Hippel Lindau (*VHL*) tumour suppressor gene alleles [14]. A VHL loss of function leads to the accumulation of hypoxia-inducible factors and the subsequent upregulation of various hypoxia-inducible factor target genes such as *VEGF*, which drives tumour-associated angiogenesis [15]. As a result, VEGFRs have emerged as major targets for mRCC treatment. Several integrated whole-genome analyses have suggested that 3p deletion and compensatory 5q extension occur at the onset of ccRCC carcinogenesis in 30–60% of cases [16]. In addition, the integrated gene analysis of Japanese patients with ccRCC performed by Sato et al. revealed that the long arm of chromosome 5 was amplified in 60% of ccRCC cases [14]. While abnormalities in *VHL* and chromatin repair genes on 3p have been widely investigated, the biological significance of 5q extension remains unknown. In this study, we focused on fibroblast growth factor receptor 4 (*FGFR4*), a gene present on the long arm of chromosome 5, whose amplification is expected to have functional consequences.

FGFR is a single-pass transmembrane protein consisting of an extracellular domain with an immunoglobulin-like structure, a transmembrane domain, and an intracellular domain with two tyrosine kinase domains. There are four FGFR types (FGFR1–FGFR4), each of which is encoded by distinct genes. Ligand-dependent FGFR dimerisation leads to the activation of multiple intracellular signalling pathways, including the extracellular signal-related protein kinase 1/2 (ERK1/2) pathway, Akt/mTOR pathway, and STAT3 pathway [17, 23]. This, in turn, promotes cell growth, migration, and survival. *FGFR4* is widely expressed in the embryonic stage and functions in tissue differentiation and organogenesis [18]. The expression of *FGFR4* after birth is limited to the liver, lungs, and bones, with functions in the regulation of bile acid production, metabolism, muscle differentiation, and tissue repair [19, 20]. Previous reports indicate that FGFR4 is overexpressed in cancers such as breast cancer, rhabdomyosarcoma, and ovarian cancer [21, 22]. Furthermore, several studies have demonstrated that FGFR4 inhibition suppresses tumour growth in hepatocellular, ovarian, and oesophageal cancers [22–24]. Treatment with FGFR4-specific inhibitors was reportedly well tolerated by mice [25]. However, the significance of FGFR4 in ccRCC remains unclear. Hence, we sought to evaluate the function of FGFR4 in ccRCC, with a focus on its direct effects on tumour cell proliferation and the potential of selective pharmacological FGFR4 inhibition as a therapeutic strategy.

## Materials and methods

### Protein expression of FGFR4 in clinical ccRCC specimens

We analysed the expression of FGFR4 in 74 human renal cancer surgical specimens using immunohistochemical staining. We examined specimens of patients who were histologically diagnosed to have ccRCC by a pathologist between 2010 and 2016 at Yamagata University Hospital (Table S1). This study was approved by the Ethical Committee of Yamagata University (H30-534) based on the tenets of the Declaration of Helsinki, and informed consent for the use of clinical specimens was obtained from all patients. Immunohistochemical staining and slide preparation were performed as previously described [26].

**Fig. 1:**
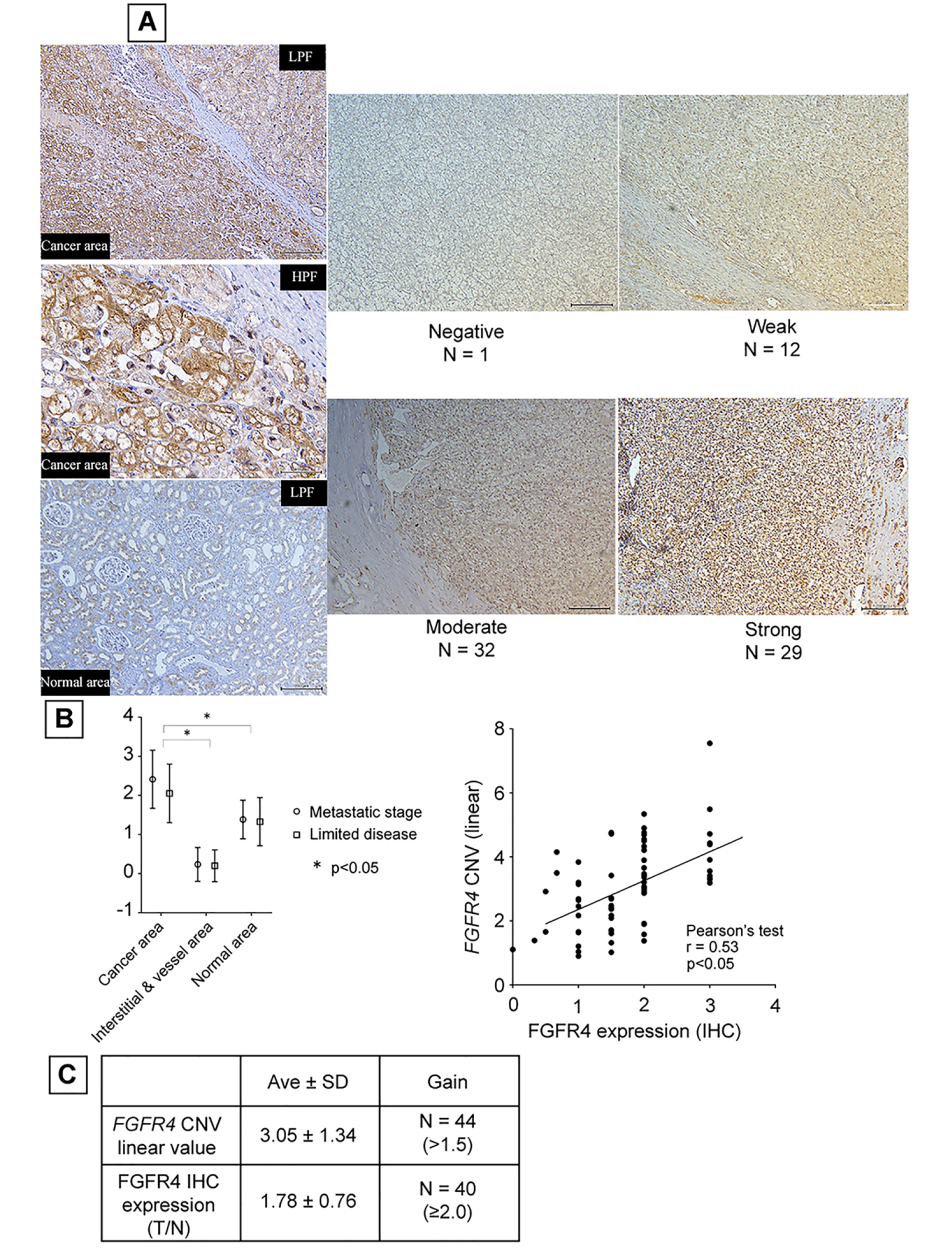
**Relationship between*FGFR4*copy number and protein expression in ccRCC clinical specimens**. **A**, Immunohistochemistry of ccRCC clinical specimens. Representative slide of the four-point scale evaluation shown (upper row), and staining characteristic of cancer and non-cancerous areas are shown in the lower panel. **B**, Comparison of FGFR4 expression among ccRCC clinical stages. Standardised staining score were estimated by regions (cancer, interstitial and vessel, normal area) and compared by clinical stage. **C**, Comparison between estimated *FGFR4* copy number and FGFR4 protein expression. Each average value is shown (left) and the correlation diagram is shown (right). Abbreviations: FGFR4, fibroblast growth factor receptor 4; ccRCC, clear cell renal cell carcinoma

Formalin-fixed paraffin-embedded (FFPE) tissue blocks were sliced into 3-mm thick sections. After mounting the thin sections onto silane-coated glass slides (Dako, Tokyo, Japan), the slides were baked for 1 h on a 60 °C thermal plate. The slides were then immersed in xylene (three times for 5 min each) as well as in 100% ethanol (three times for 5 min each) to deparaffinate and dehydrate before hydrating again. Thereafter, the slides were submerged in 10 mM citrate buffer (pH 6.0) and autoclaved at 120 °C for 10 min to retrieve antigens. This was followed by immersion in methanol containing 3% hydrogen peroxide for 10 min to inhibit endogenous peroxidase activity. The slides were then immersed in PBS containing 1% bovine serum albumin to block non-specific primary antibody binding. The slides were then incubated with an anti-FGFR4 rabbit polyclonal antibody (1:100, HPA027369; Sigma-Aldrich, St. Louis, MO, USA) at 4 °C in a moist environment overnight, followed by washing with PBS. To visualise the secondary antibody reaction, the sections were stained with N-Histofine Simple Stain MAX-PO® (Nichirei Biosciences Inc., Tokyo, Japan) for 30 min at room temperature, followed by washing and reacting with 3,3ʹ-diaminobenzidine tetrahydrochloride (D5905; Sigma-Aldrich) for the colour reaction. The nuclei were counterstained with haematoxylin. The prostate tissues were used as negative exogenous controls, normal renal tubule tissues were used as positive exogenous controls, and red blood cells from normal renal glomeruli were used as negative endogenous controls. Staining was evaluated using a four-point scale (negative – no staining: 0 pt, weak – stains to the same degree as the normal renal tubule: 1 pt, moderate – stains stronger than the normal renal tubule: 2 pt, strong – stains considerably stronger than the normal renal tubule: 3 pt, Fig. 1A, top), and scores were calculated. To standardise the quantification of expression, the ratios of the aforementioned tumour and normal renal tubule scores were calculated and used for analysis.

### DNA extraction and gene copy number analysis

After thawing each ccRCC specimen preserved in RNA at ˗80 °C, DNA extraction was performed according to the recommended protocol using the Easy DNA Extraction Kit® (KANEKA, Tokyo, Japan). DNA concentration was measured using the NanoDrop Lite spectrometer® (Thermo Fisher Scientific, Waltham, MA, USA). The DNA concentration of each specimen was adjusted to 5 ng/µL using deuterium depleted water. The PCR components included the TaqMan Copy Number Assays® (ID: Hs01973966_cn, Life Technologies, Carlsbad, CA, USA) for FGFR4 detection, the TaqMan RNaseP control reagent® (Life Technologies) as an endogenous control, and the TaqMan Genotyping Master Mix® (Life Technologies) as the substrate. Multiplex PCR mixtures, of total volume 20 µL, contained the TaqMan Genotyping Master Mix (Life Technologies), RNaseP Primer-Probe (VIC dye) Mix, FGFR4 Primer-Probe Mix (FAM dye), and 5 ng of genomic DNA. All experiments were conducted in triplicate. The PCRs were performed on the ABI7300 Fast Real-Time PCR System with TaqMan detector® (Life Technologies), and data were analysed using Copy Caller software version 2.1® (Thermo Fisher Scientific). Detection of 2.5 or more copies of the gene indicated copy number amplification.

### Analysis of the relationship between ***FGFR4*** copy number and mRNA expression from The Cancer Genome Atlas (TCGA) datasets

We investigated the correlation between *FGFR4* copy number and mRNA expression in TCGA cohort. TCGA data on *FGFR4* copy alterations and RNA-Seq values for ccRCC were downloaded from cBioportal in June 2018. Data of 522 patients were retrieved. Cases without sufficient data on copy number alterations and RNA-Seq or outliers determined were excluded based on the Smirnov–Grubbs test.

### Cells and culture

RCC cell lines A498, A704, 769-P, 786-O, and ACHN were purchased from ATCC (Manassas, VA, USA), and the human normal renal cortical epithelial cell line HRCEpC was purchased from PromoCell (Heidelberg, Germany). The cells were cultured as described previously [26]. RPMI 1640 medium (containing 10% foetal bovine serum and 50 µg/mL kanamycin) was used for RCC cell lines, and Renal Epithelial Cell Growth Medium 2 was used for HRCEpC, both in the presence of 5% CO_2_ at 37 °C in an incubator with high humidity. For cell migration and recovery, trypsin-EDTA (T4049, Sigma-Aldrich) was used, and PBS was used as the wash solution before subculture and during the various tests.

We used T-25 flasks for cell culture and transfection. We seeded 2.0 × 10^5^ cells/well in six-well plates and 1.0 × 10^3^ cells/well in 96-well plates. Cell treatments were performed 24 h after cell seeding, and cell adhesion stability was confirmed using microscopy. Each evaluation was performed when the cells reached 80% confluency.

### Western blotting

Western blotting was performed as previously described [27]. The antibodies used included α-tubulin (1:500; Wako Pure Chemical, Osaka, Japan), FGFR4, pFGFR (Tyr653/654), Akt, pAkt (Ser437), ERK1/2, pERK1/2 (Thr202/Tyr204), STAT3, pSTAT3 (Tyr705), 4eBP1, p4eBP1 (Thr70), S6RP, and pS6RP (Ser235/236) (1:1000; Cell Signaling Technology Japan, Osaka, Japan). TBS-T containing 3% foetal bovine serum (PBS containing 0.05% Tween 20) was used to dilute the antibodies.

### RNA interference

*FGFR4* knockdown was achieved in A498, A704, and 769-P cells using two siRNAs: 5ʹ-CAUUGACUACUAUAAGAAATT-3ʹ and 5ʹ-CCACCACAUUGACUACUAUTT-3ʹ (Thermo Fisher Scientific, Tokyo, Japan). An unrelated control siRNA was used (Applied Biosystems, Thermo Fisher Scientific, Tokyo, Japan). Transfection was carried out using Lipofectamine RNAiMax (Invitrogen, Thermo Fisher Scientific, Inc., USA) according to the manufacturer’s recommendations.

### Pharmacological FGFR4 inhibition study

BLU9931 (Cayman Chemical, Ann Arbor, MI, USA) was used for pharmacological FGFR4 inhibition. It was diluted in DMSO and stored according to the manufacturer’s recommendations.

### Cell viability analysis

We analysed cell viability after siRNA-mediated or pharmacological FGFR4 inhibition via an MTS assay using the CellTiter 96® Aqueous One Solution Cell Proliferation Assay (Promega, Madison, WI, USA) as previously described [28]. We performed all MTS assays in six wells per group, for three times.

### Assessment of apoptotic cell death

The apoptosis assay was performed as follows. A498 cells were cultured in the presence or absence of BLU9931 and observed over time. First, we used flow cytometry (FACS Canto II, BD Bioscience, San Jose, CA, USA) to assess cells for the loss of cell membrane phospholipid asymmetry, thereby detecting apoptosis via dual staining with the FITC Annexin V Apoptosis Detection Kit (BD Bioscience) and propidium iodide (Sigma-Aldrich). The cells were also morphologically assessed under an inverted fluorescent microscope 4 h after treatment with 10 µM BLU9931. Next, the progression of apoptosis was determined using 10 µM CellEvent™ Caspase-3/7 Green Detection Reagent and 100 nM tetramethylrhodamine ethyl ester (TMRE) (Thermo Fisher Scientific, Tokyo, Japan), which reflects mitochondrial membrane polarity. We used an inverted fluorescence microscope (BZ-X800; KEYENCE, Osaka, Japan) for the observation of caspase-3/7 fluorescence and TMRE decrease. FACS and fluorescence microscopy observations were performed in triplicate.

### Pharmacological inhibition in a xenograft mouse model of ccRCC

All procedures were performed using BALB/c-nu mice according to the animal welfare regulations of Yamagata University Faculty of Medicine based on the “Guidelines for the implementation of animal experiments” established by the Ministry of Education, Culture, Sports, Science, and Technology of Japan. A498 cells were resuspended in a mixed solution of RPMI1640 and Matrigel (adjusted to 1.8 × 10^7^ cells/200 µL; Corning Life Sciences, Corning, NY, USA). The cells were then subcutaneously injected into the right flank of 6-week-old female mice, as described previously [29]. When the tumour volume reached approximately 400 mm^3^, mice were randomised to the BLU9931 treatment and control groups. The treatment arm has two different dose groups: 30 mg/kg and 100 mg/kg groups, against each control. The mice were treated with BLU9931 (30 mg/kg and 100 mg/kg body weight; five times per week) or vehicle (0.5% carboxymethyl cellulose), orally administered once a day, five times per week, for 2 weeks. Animal health was monitored daily, and palpable tumours were measured every 2–3 days with a calliper. Tumour volume was calculated as (L × W2) × 0.5. All mice were sacrificed via cervical dislocation under anaesthesia using isoflurane. Xenograft tumours were removed after sacrifice, and FFPE sections were prepared. These sections were stained for Ki-67, CD34, and D2-40 (DAKO, Tokyo, Japan), following the above immunohistochemistry procedure.

### Statistical analysis

Statistical analyses were performed using GraphPad Prism7 and R version 3.3.1. We used the Kruskal–Wallis test to analyse the association between FGFR4 immunohistochemical staining and clinical stage, as well as for the comparison of viability between cells with or without *FGFR4* knockdown. The correlation between *FGFR4* copy number variation (CNV) and protein expression was estimated using Pearson’s test. A *P*-value of < 0.05 was considered to indicate statistical significance.

## Results

### ***FGFR4*** copy number and protein expression

We analysed the expression of FGFR4 in 74 human clear cell carcinoma surgical specimens from patients with renal cancer using immunohistochemical staining. FGFR4 was detected in the cytoplasm and cell membrane of RCC cells but was not abundant in vessels and interstitial tissues (Fig. 1A). There was a significant difference in FGFR4 expression between the ccRCC area and control tissues, but no difference was observed between clinical stages, as FGFR4 expression was consistent from limited ccRCC to advanced-stage disease (Fig. 1B).

Sixty-one cases exhibited moderate or strong FGFR4 protein expression within the clear cell cancer region. We estimated the ratio of FGFR4 expression between the cancer region and normal renal epithelium areas (T/N ratio) as a quantitative index of FGFR4 expression, with an average FGFR4 T/N ratio of 1.786 and a standard deviation of 0.76. Forty cases (54%) exhibited high FGFR4 expression when the cut-off value was set at 1.5.

Forty-four out of 74 specimens (59.5%) presented *FGFR4* copy number amplification. The increased *FGFR4* copy number correlated with higher FGFR4 protein expression (Pearson’s correlation test; r = 0.5371 and *P* < 0.01, Fig. 1C). To validate this finding, we analysed the relationship between *FGFR4* CNV and FGFR4 mRNA in TCGA database, confirming the correlation in this external dataset (r = 0.239, *P* < 0.01) (Fig. S1).

### Analysis of ***FGFR4*** copy number and FGFR4 protein expression in RCC cell lines

**Fig. 2:**
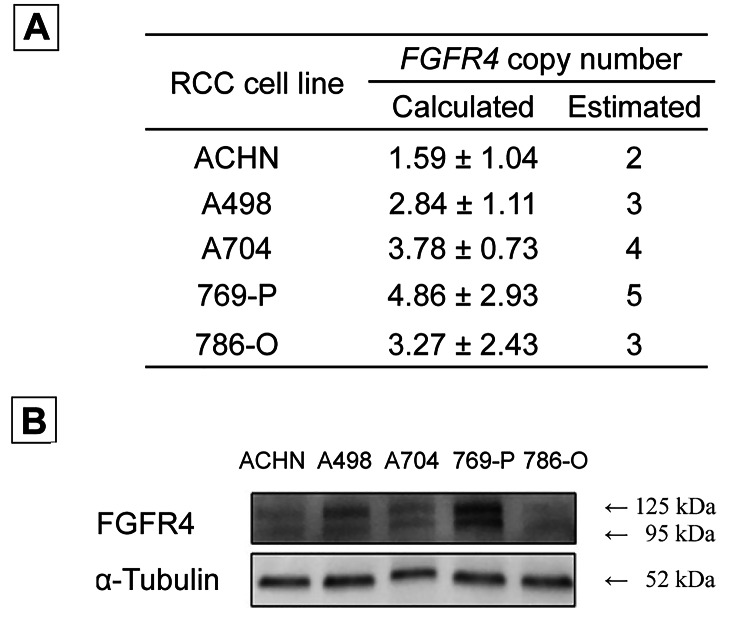
**Comparison of*FGFR4*copy number and FGFR4 protein expression in RCC cell lines**. **A**, FGFR4 copy number in RCC cell lines. **B**, Western blot analysis of FGFR4 protein expression in RCC cell lines. Abbreviations: FGFR4, fibroblast growth factor receptor 4; RCC, renal cell carcinoma

We analysed FGFR4 protein expression with respect to *FGFR4* copy number in RCC cell lines. While the papillary RCC cell line ACHN exhibited normal CNV, all ccRCC cell lines (A498, A704, 769-P, and 786-O) harboured an *FGFR4* copy number amplification (Fig. 2A). The FGFR4 levels in 769-P cells were the highest, reflecting the relationship between CNV and FGFR4 protein expression (Fig. 2B).

### ***FGFR4*** knockdown

**Fig. 3:**
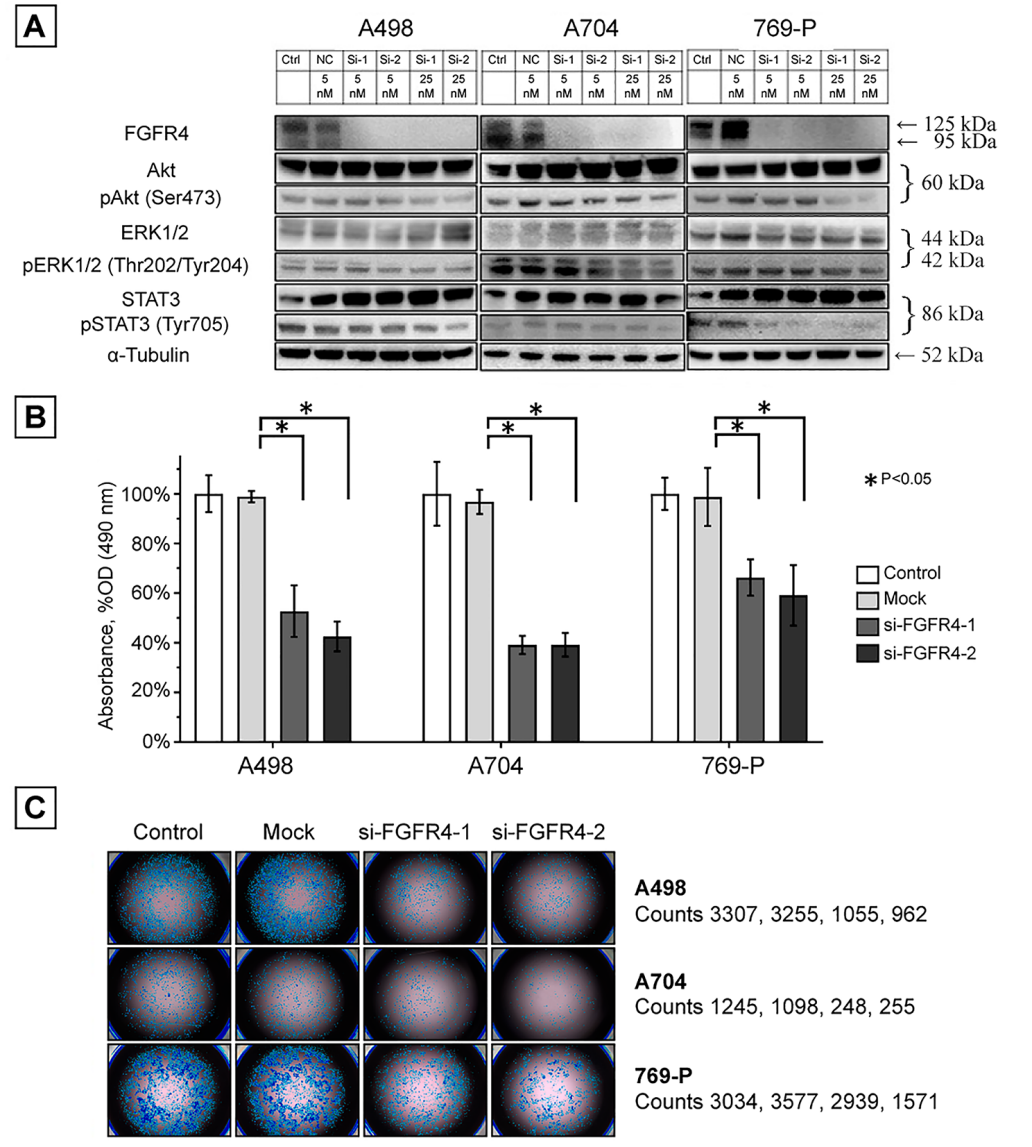
***FGFR4*knockdown in ccRCC cell lines**. **A**, Immunoblot analysis of Akt/mTOR, ERK1/2, and STAT3 pathway phosphorylation after *FGFR4* knockdown. **B**, MTS assay in ccRCC cell lines 72 h after *FGFR4* knockdown. **C**, Morphological observation using an inverted fluorescence phase-contrast microscope before the MTS assay (left). Cells were counted using dedicated imaging software (right). Abbreviations: FGFR4, fibroblast growth factor receptor 4; RCC, renal cell carcinoma; mTOR, mammalian target of rapamycin; ERK, extracellular signal-regulated protein kinase; STAT, signal transducer and activator of transcription

To investigate the function of *FGFR4*, cancer cell viability and downstream molecule phosphorylation were investigated under RNA-mediated FGFR4 inhibition. Western blotting revealed that the phosphorylation of AKT/mTOR, ERK1/2, and STAT3 was suppressed in *FGFR4* knockdown cells (Fig. 3A). The viability of knockdown cells (subjected to 5-nM siFGFR4 transfection) was decreased compared with that of the controls (Fig. 3B), as confirmed by the lower *FGFR4* knockdown cell counts (Fig. 3C).

### Pharmacological FGFR4 inhibition using BLU9931

**Fig. 4:**
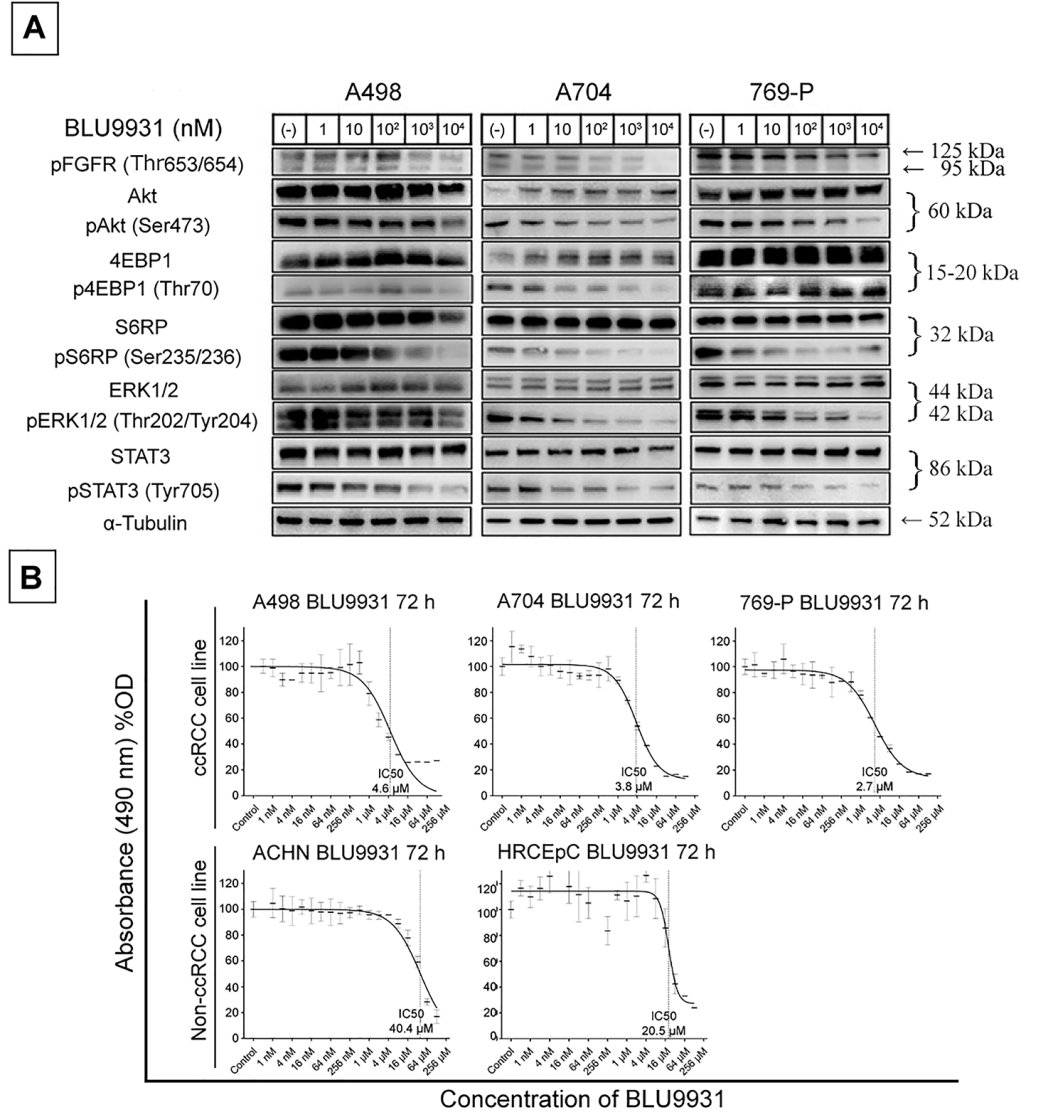
**Selective pharmacological inhibition of FGFR4 by BLU9931**. **A**, Western blot analysis of intracellular signalling pathways Akt/mTOR, ERK1/2, and STAT3 3 h after BLU9931 treatment. The phosphorylation level transition of each signal pathway is shown according to the concentration gradient of BLU9931. **B**, MTS assay 72 h after BLU9931 treatment in ccRCC cell lines (A498, A704, 769-P), a non-ccRCC cell line (ACHN), and a normal renal cortical cell line (HRCEpC). ccRCC cell growth suppression was observed via the concentration gradient of BLU9931 at a lower level compared to HRCEpC cell suppression. Abbreviations: FGFR4, fibroblast growth factor receptor 4; RCC, renal cell carcinoma; mTOR, mammalian target of rapamycin; ERK, extracellular signal-regulated protein kinase; STAT, signal transducer and activator of transcription

To further confirm the function of FGFR4 and assess its potential as a therapeutic target in ccRCC, we used the small molecule inhibitor BLU9931, which selectively inhibits FGFR4 kinase activity. Western blot analysis revealed that the phosphorylation of AKT/mTOR, ERK1/2, and STAT3 was suppressed after BLU9931 treatment in RCC cell lines (Fig. 4A). While the IC50 values for the non-ccRCC cell line ACHN and the human normal renal cell line HRCEpC were 40.4 µM and 20.5 µM, respectively, those for ccRCC cell lines A498, A704, and 769-P were considerably low, at 4.6 µM, 3.8 µM, and 2.7 µM, respectively (Fig. 4B).

### BLU9931 treatment leads to apoptotic cell death

**Fig. 5:**
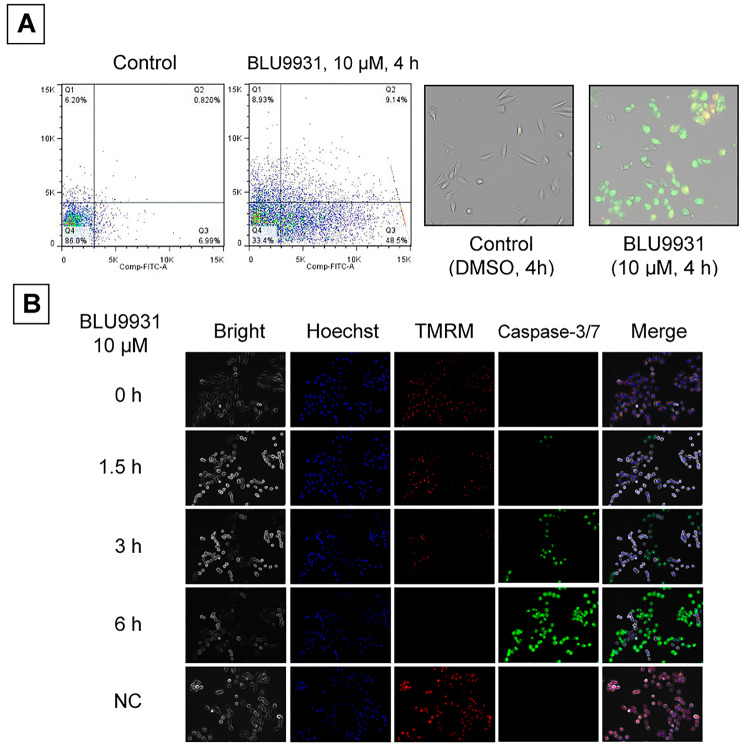
**Cell death after BLU9931 treatment**. **A**, Loss of cell membrane phospholipid asymmetry observed via Annexin V detection after BLU9931 treatment. **B**, Time-course assessment of caspase-3/7 and mitochondrial polarity (TMRM) after BLU9931 treatment. Time-course morphological observation under an inverted fluorescence microscope. Abbreviations: TMRM, tetramethylrhodamine ethyl ester

To investigate the apoptotic effect of BLU9931, we analysed Annexin V expression in A498 cells 48 h after BLU9931 treatment. Flow cytometry data indicated that the FITC-Annexin V^+^ fraction increased after treatment (Fig. 5A). We then observed changes in the mitochondrial membrane potential and caspase-3/7 activity after BLU9931 treatment. The mitochondrial membrane potential observed via TMRM decreased 1.5 h after treatment, whereas the caspase-3/7 levels increased (Fig. 5B). These findings indicate that BLU9931 induces apoptotic cell death.

### Therapeutic efficacy of BLU9931 in an A498 xenograft mouse model

**Fig. 6:**
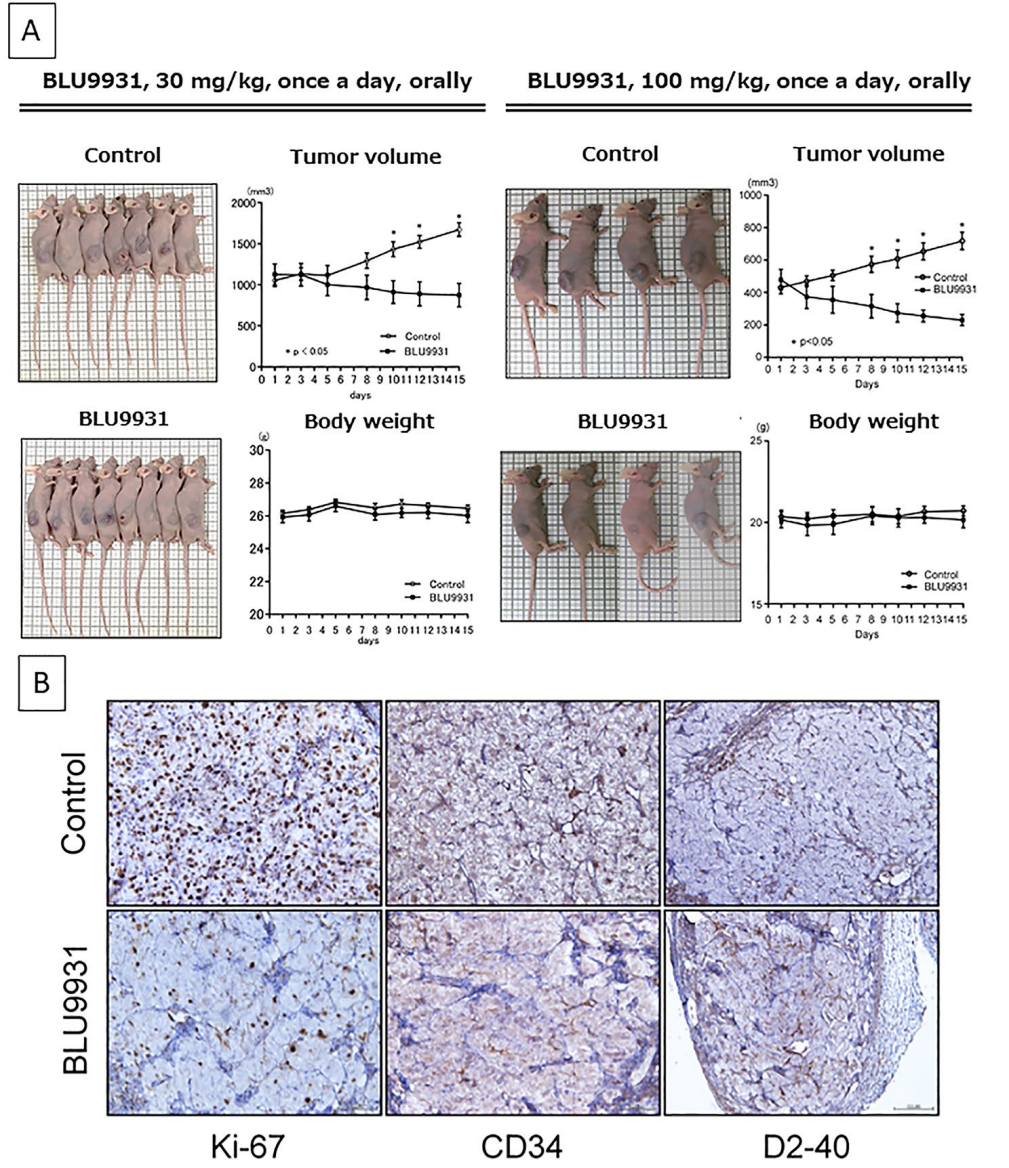
**A498 xenograft mouse model treated with the FGFR4 selective inhibitor BLU9931.**. **A**, Mice at the end of the study, tumour volume, and body weight course. **B**, Ki-67, CD34, and D2-40 expression in mouse tumours was assessed via immunohistochemistry

To investigate the anti-cancer effect of BLU9931 in vivo, we subcutaneously implanted mice with A498 xenografts. The tumour size decreased 3 days after the initiation of treatment. The two treatment groups showed similar results, compared with the control groups. A significant difference in tumour volume between the 30 mg/kg and 100 mg/kg groups was observed on days 10 and 8, respectively (Fig. 6A). No weight loss or clear adverse events were observed in mice during the treatment (Fig. 6A). Furthermore, the expression of the proliferation marker Ki67 was suppressed in the BLU9931 treatment group. In contrast, the expression of the neovascularisation marker CD34 was slightly low, whereas no significant difference was observed in the expression of the lymphatic endothelium marker D2-40. These observations suggest that BLU9931 mainly affects ccRCC cells rather than vessels and lymphatic endothelium (Fig. 6B).

## Discussion

Several studies have reported FGFR4 as a potential therapeutic target in hepatic, ovarian, and oesophageal cancers. A phase I clinical trial of the highly selective FGFR4 inhibitor fisogatinib for the treatment of hepatic cancer with the amplification of *FGF19*, encoding an FGFR4 ligand, is currently on-going [30]. A comprehensive analysis of ccRCC genetic alterations revealed that 60% of ccRCC tumours harboured a copy number amplification of *FGFR4* [14]. Nevertheless, the function of FGFR4 in ccRCC remains unclear. To the best of our knowledge, this is the first report on the function of FGFR4 in ccRCC. In this study, we showed that (1) 60% of ccRCC specimens harboured an *FGFR4* copy number amplification; (2) *FGFR4* copy number correlated with FGFR4 protein expression; (3) FGFR4 inhibition suppressed intracellular signalling pathways, including ERK1/2, Akt/mTOR, and STAT3 pathways; (4) the selective FGFR4 inhibitor BLU9931 suppressed tumour growth, leading to apoptotic cell death in vitro; and (5) BLU9931 treatment shrunk tumours in ccRCC xenograft mice.

The TRACERx RENAL group reported that chromothripsis on 3p occurs as an initial event in ccRCC development, with 30–40% of chromothripsis cases accompanied by 5q extension [16]. In addition, Sato et al. demonstrated that 60% of ccRCC specimens from Japanese patients harboured a 5q gain and a copy number amplification of *FGFR4* on 5q [14]. Consistent with this report, 60% of ccRCC cases in our Japanese cohort were confirmed to have an *FGFR4* copy number amplification. These findings indicate that *FGFR4* copy number amplification is rather common and support the notion that chromothripsis occurs before tumours acquire genetic heterogeneity.

Our study demonstrated that FGFR4 inhibition suppresses intracellular signalling pathways for cell proliferation and survival. In particular, the selective FGFR4 inhibitor BLU9931 suppressed cell proliferation and led to apoptosis. The BLU9931 treatment also shrunk tumours in ccRCC xenograft mice. Importantly, the BLU9931 dose tolerated by normal cells was considerably higher than that tolerated by ccRCC cells. BLU9931 was also well tolerated in all experimental mice, with no apparent signs of toxicity. Several previous reports have supported the tolerability of FGFR4 inhibition. For example, *FGFR4*-knockout mice were reportedly healthy [30]. In addition, multiple highly selective pharmacological FGFR4 inhibitors, such as FGF-401, BLU-9931, and BLU-554, were well tolerated by xenograft mice [24, 31, 32]. These results indicate that BLU9931 holds promise as a therapeutic agent in ccRCCs harbouring *FGFR4* copy number amplifications, which account for 30–60% of total ccRCC cases.

## Conclusion

In summary, our research shows that 60% of ccRCCs harbour a copy number amplification of *FGFR4*, which encodes a receptor with critical roles in ccRCC proliferation and survival. Furthermore, we identified FGFR4 as a potential therapeutic target in ccRCCs harbouring *FGFR4* copy number amplifications.

## Electronic supplementary material

Below is the link to the electronic supplementary material.

Supplementary Material 1

## Data Availability

The datasets generated during and/or analysed during the current study are available from the corresponding author on reasonable request.
