# Supplementary material for: Fibroblast growth factor receptor type 4 as a potential therapeutic target in clear cell renal cell carcinoma
Source: BMC Cancer. 2023 Feb 20;23:170. doi: 10.1186/s12885-023-10638-3 (PMC9942348; doi:10.1186/s12885-023-10638-3)
Supplement: Supplementary file 1 — Supplementary Material 1 [file 12885_2023_10638_MOESM1_ESM.pdf]

Supplementary Table 1.
Patient characteristics for the analyzed ccRCC specimens

		Number	%
Total		74	
Median observation time, months (range)		61.15 (0.2–104.2)	
Median age (range)		63.8 (44.2–81.7)	
Sex			
	Male	60	81
	Female	14	19
Clinical T stage (UICC)			
	1a	27	36
	1b	16	22
	2a	8	11
	2b	2	3
	3a	15	20
	3b	3	4
	4	3	4
Clinical N stage (UICC)			
	0	66	89
	1	5	7
	2	3	4
Clinical M stage (UICC)			
	0	58	78
	1	16	22
Grade (Fuhrman)			
	1	15	20
	2	25	34
	3	26	35
	4	8	11
Infiltrative growth (INF)			
	α	49	66
	β	23	31
	γ	1	1
	N/A	1	1
Treatment outcome			
	Cancer-free survival	48	65
	Survival with RCC	8	11
	Cancer-specific death	14	19
	Death due to other causes	4	5

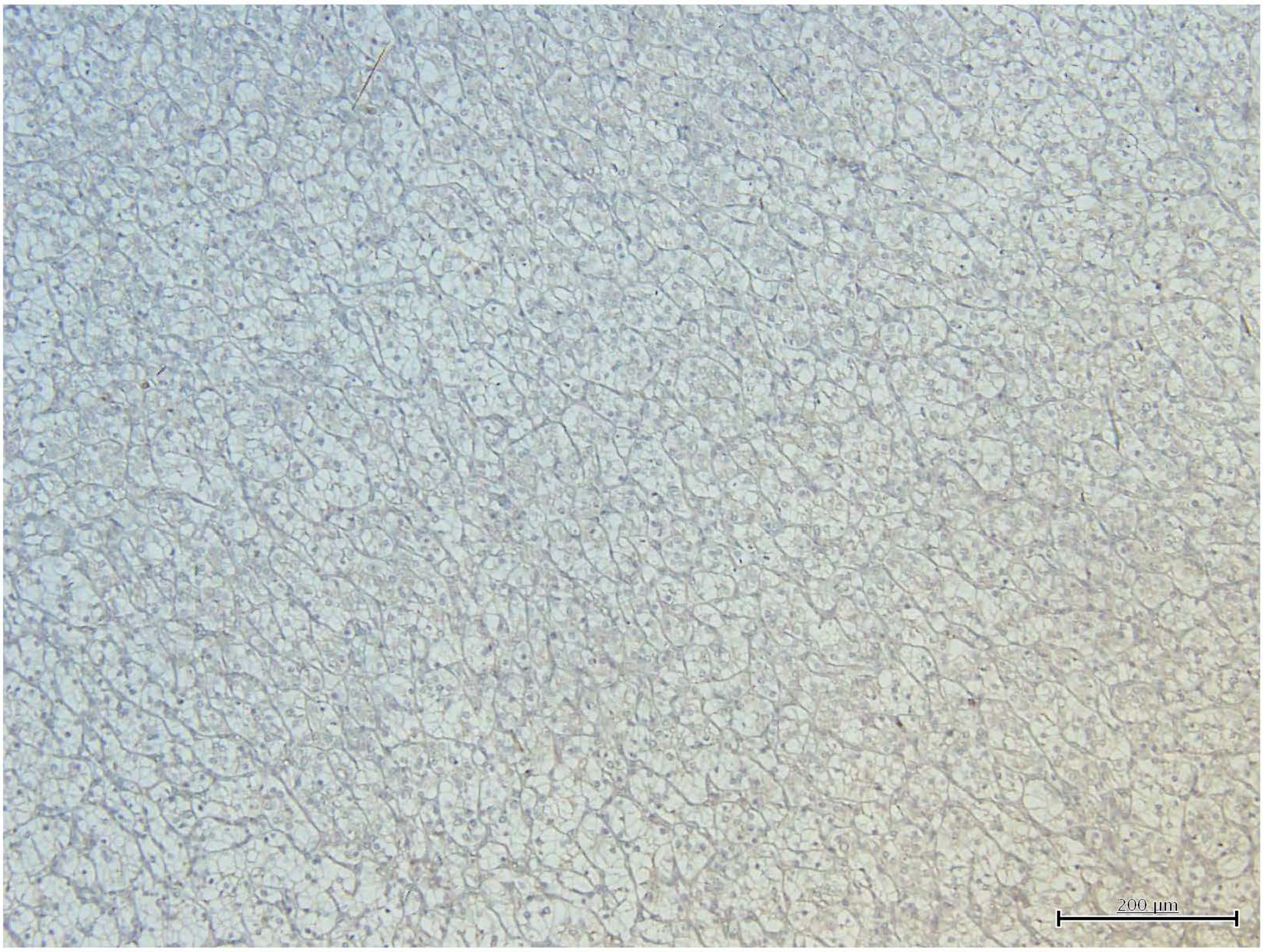

Fig S1. Uncompressed image of FGFR4 IHC (Figure 1A, Negative expression)

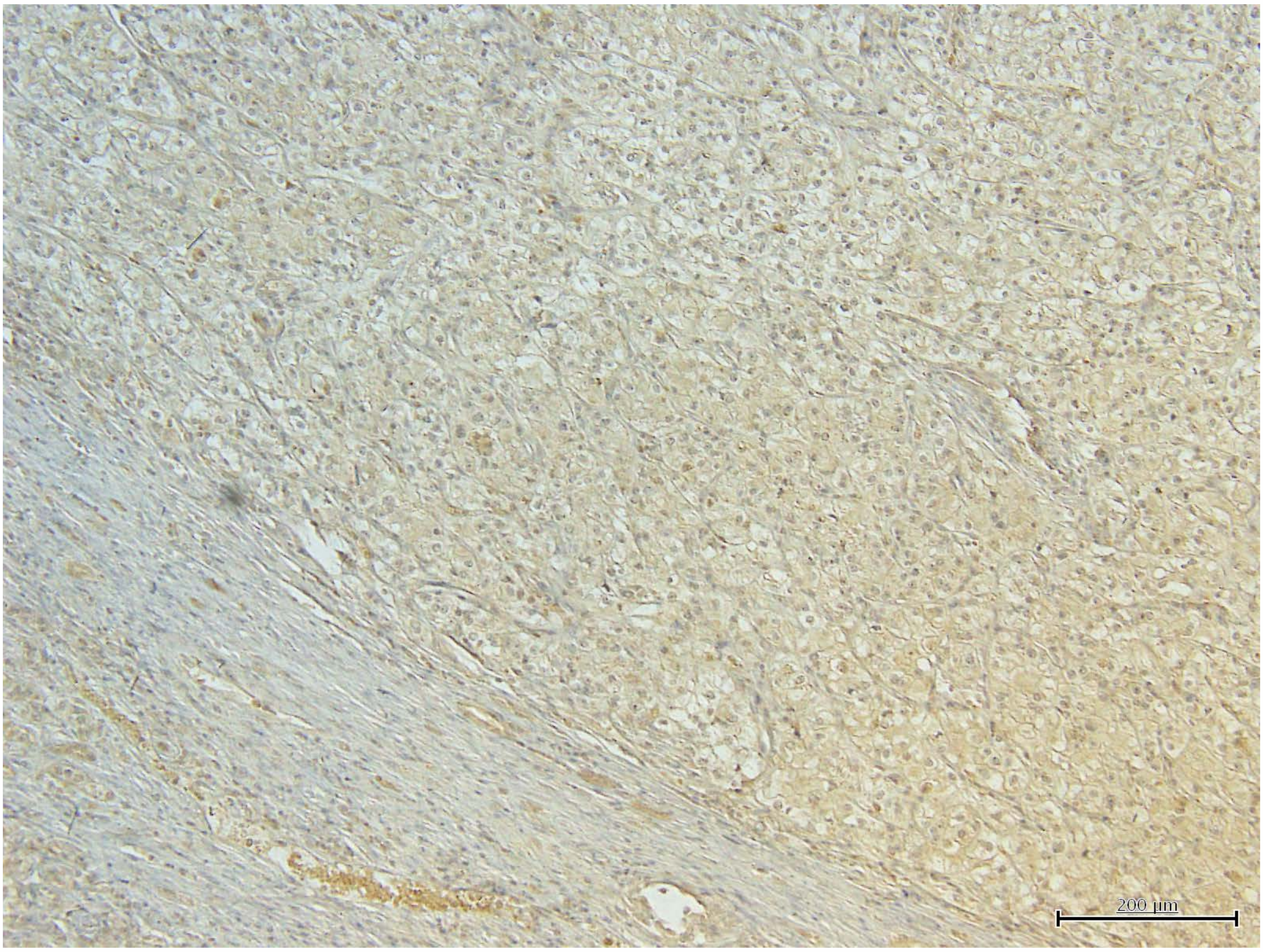

Fig S2. Uncompressed image of FGFR4 IHC (Figure 1A, Weak expression)

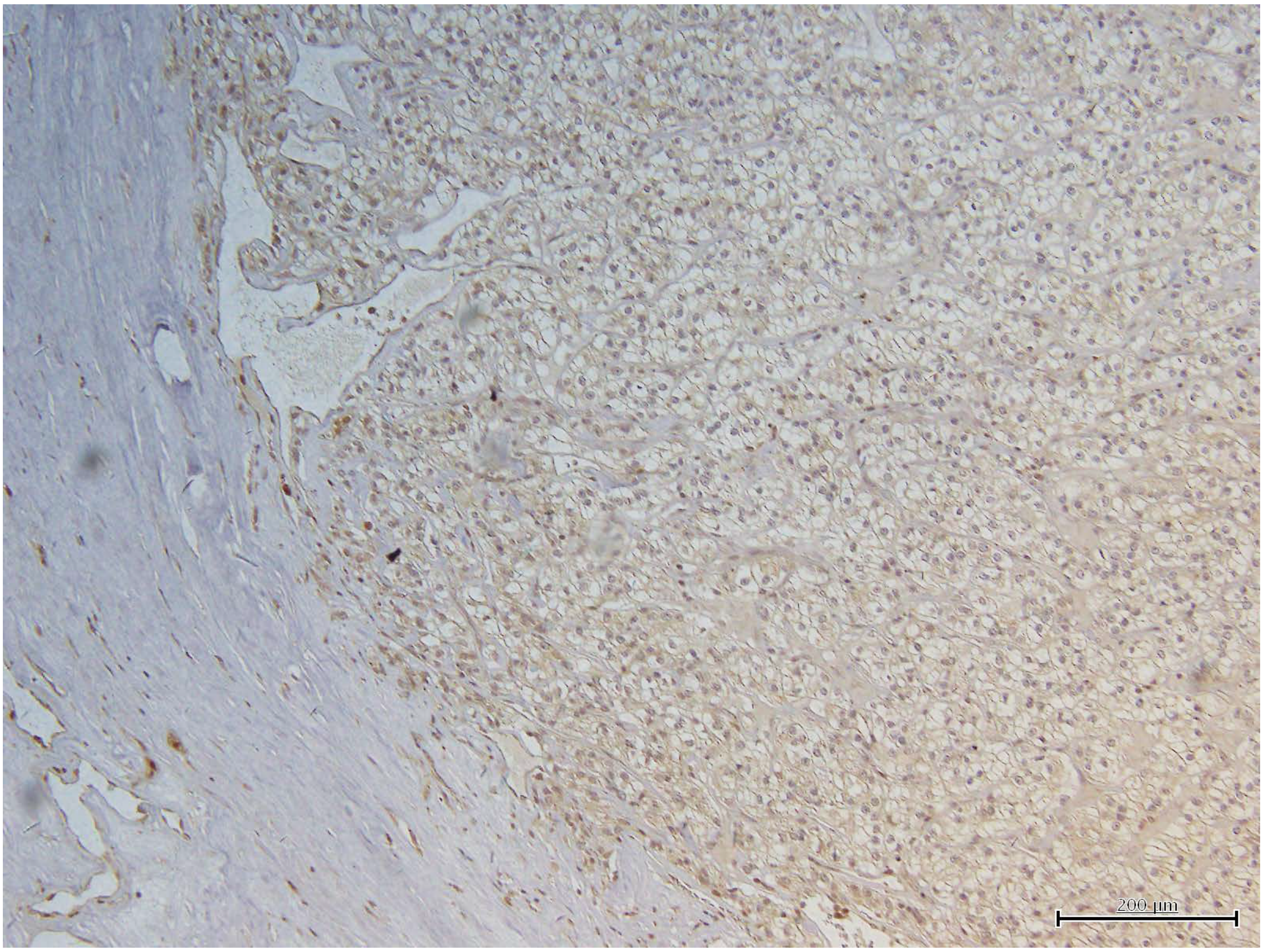

Fig S3. Uncompressed image of FGFR4 IHC (Figure 1A, Moderate expression)

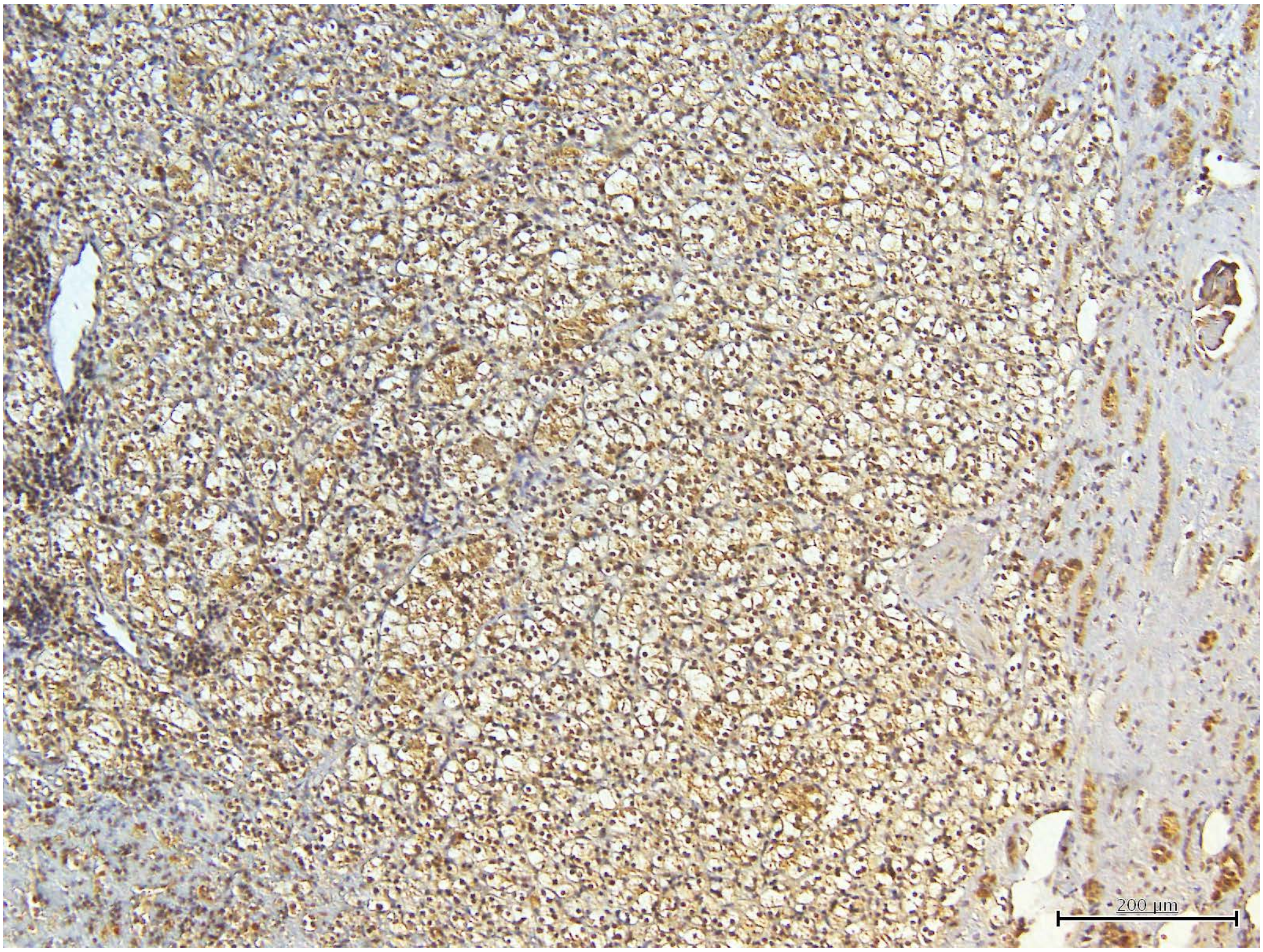

Fig S4. Uncompressed image of FGFR4 IHC (Figure 1A, Strong expression)

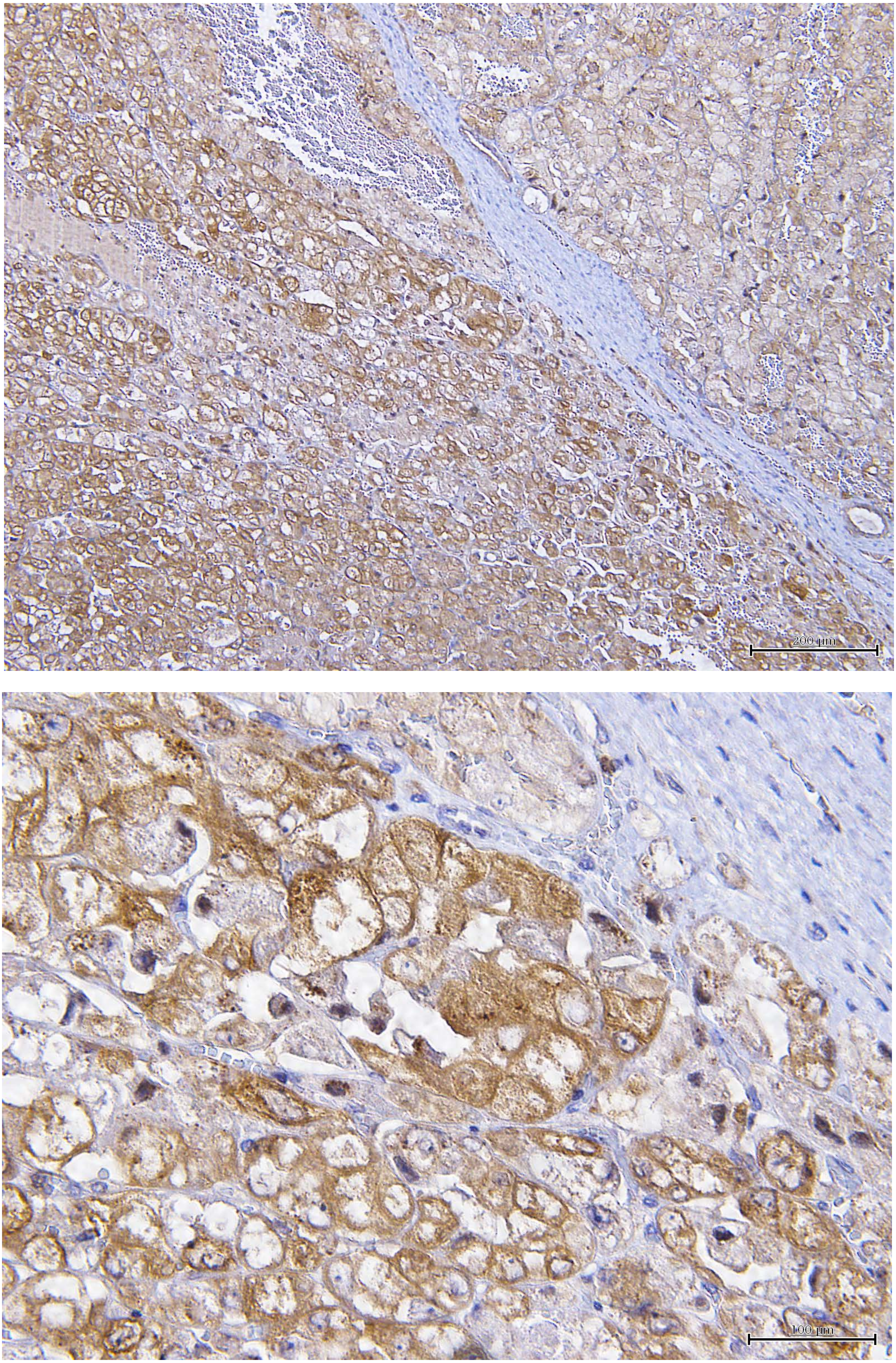

Fig S5. Uncompressed image of FGFR4 IHC (Figure 1A, Cancer area, LPF, HPF)

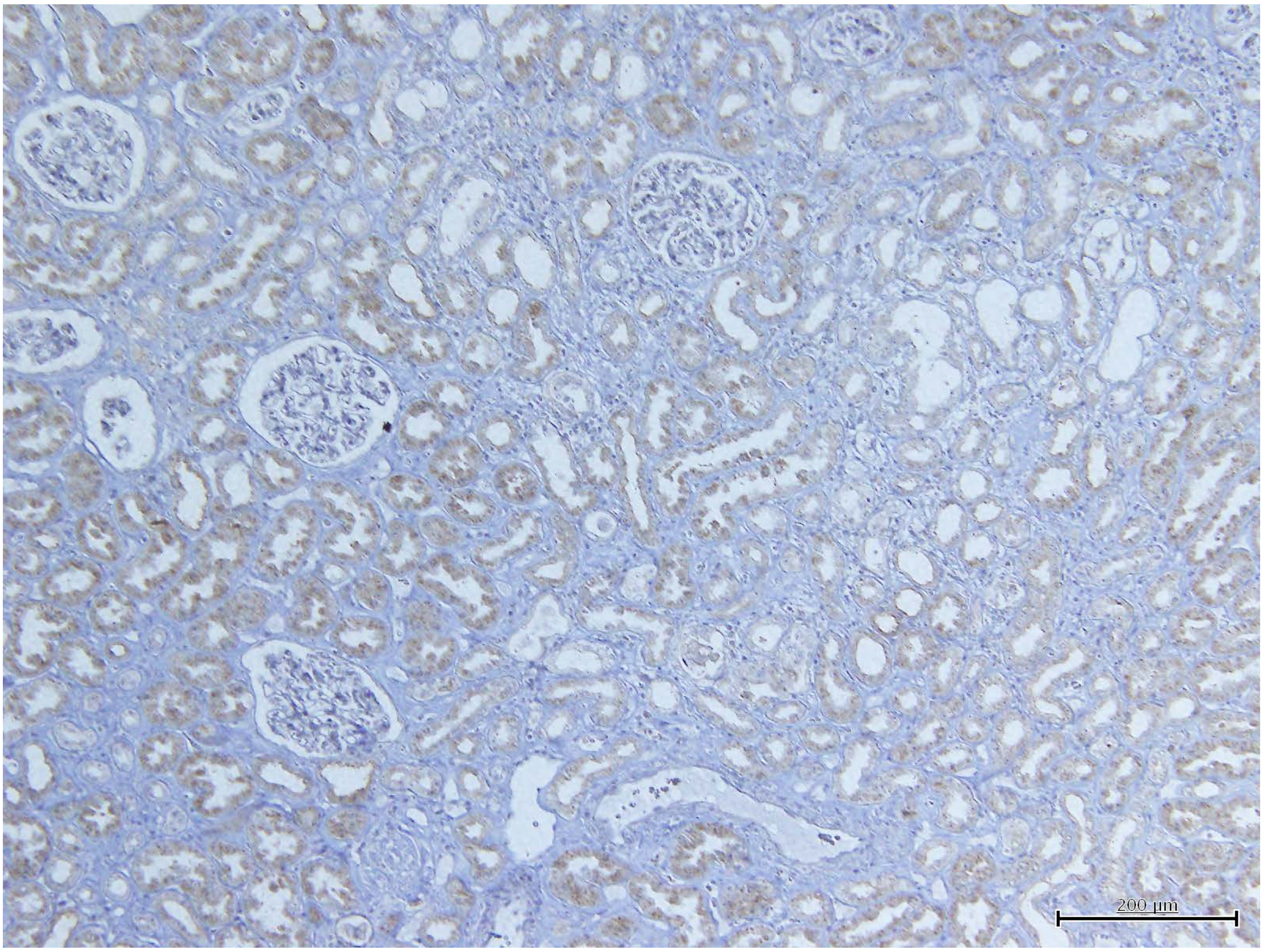

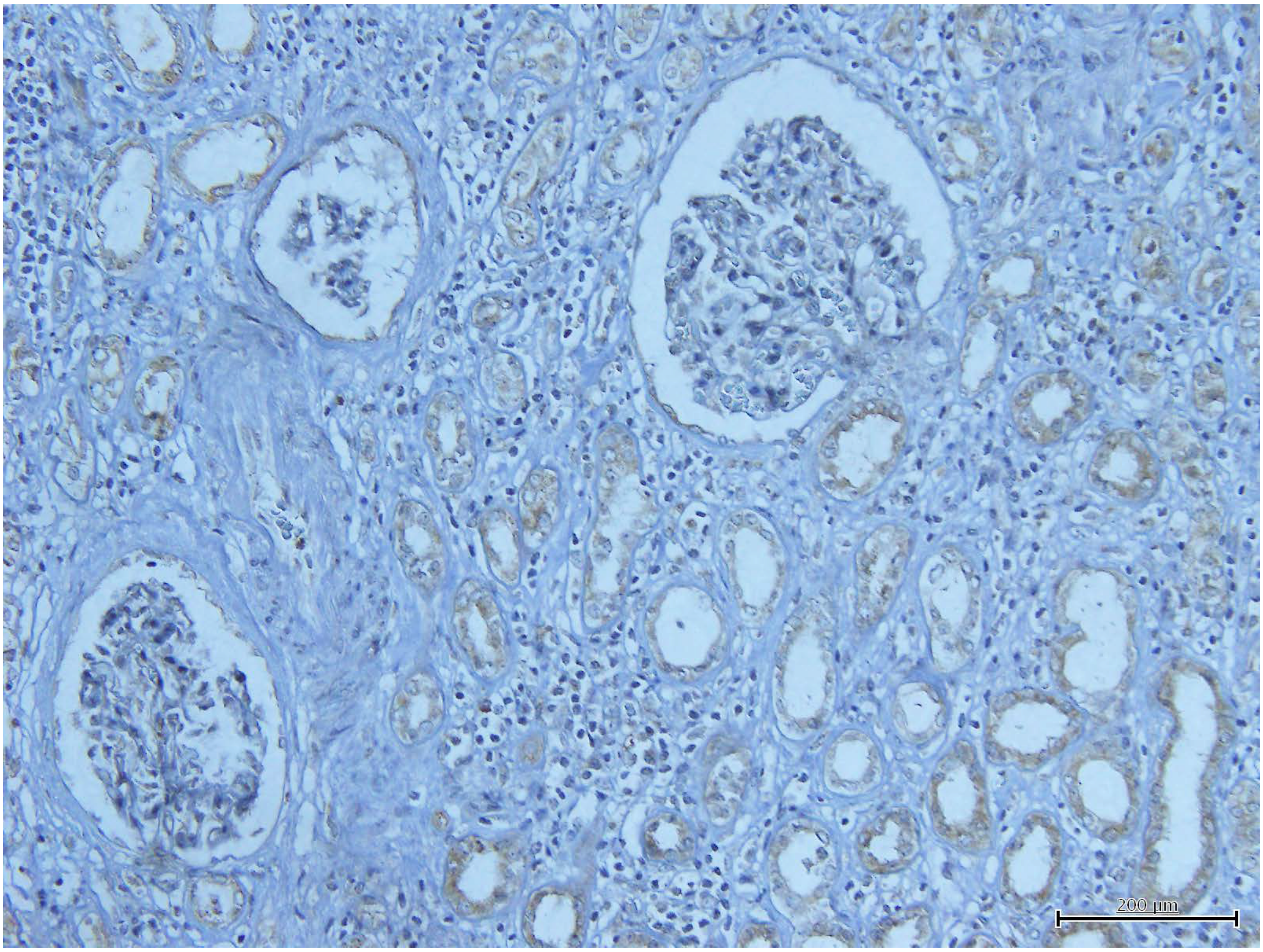

Fig S6. Uncompressed image of FGFR4 IHC (Figure 1A, Normal area)

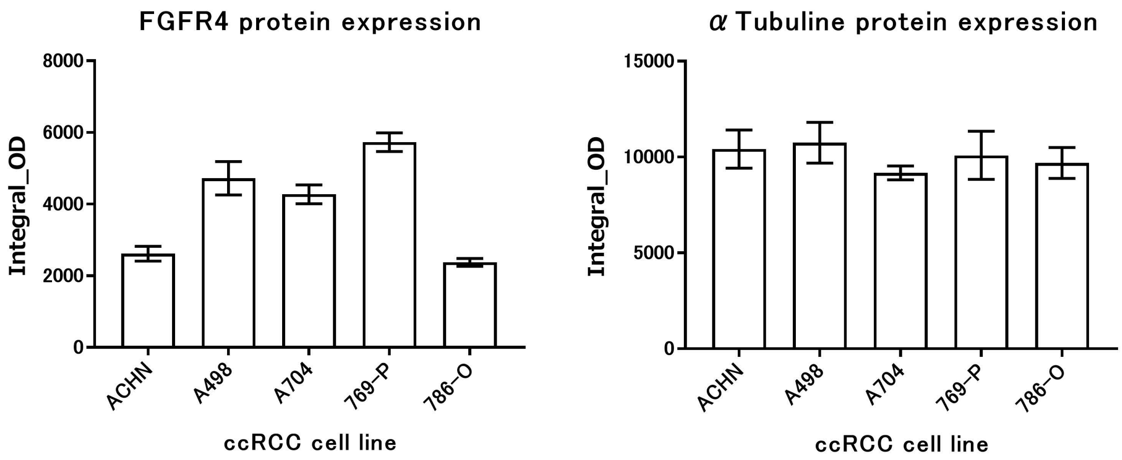

Figure S7. Densitometry analisys of western blot (Figure 2B).

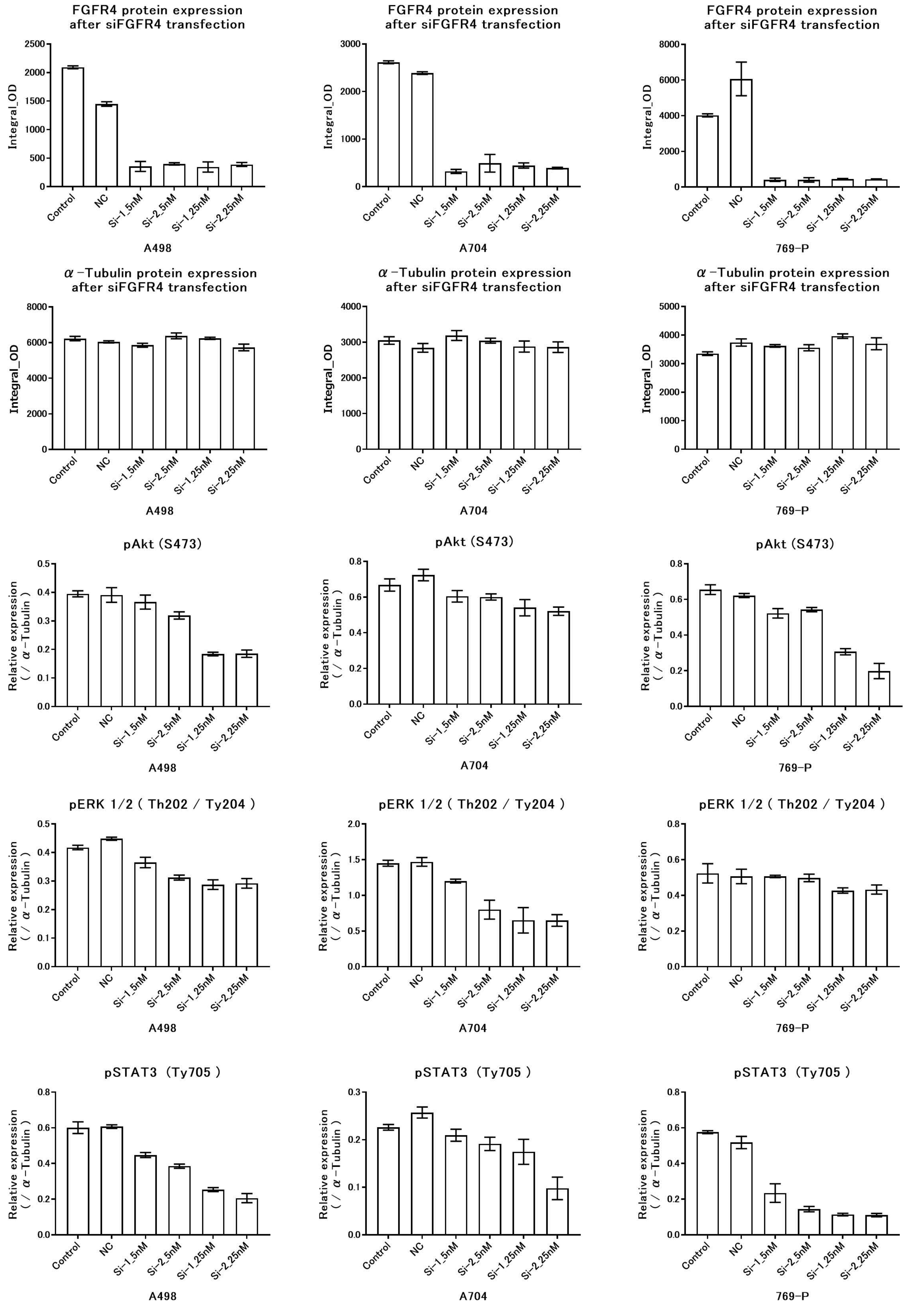

Figure S8. Densitometry analisys of western blot (Figure 3A).

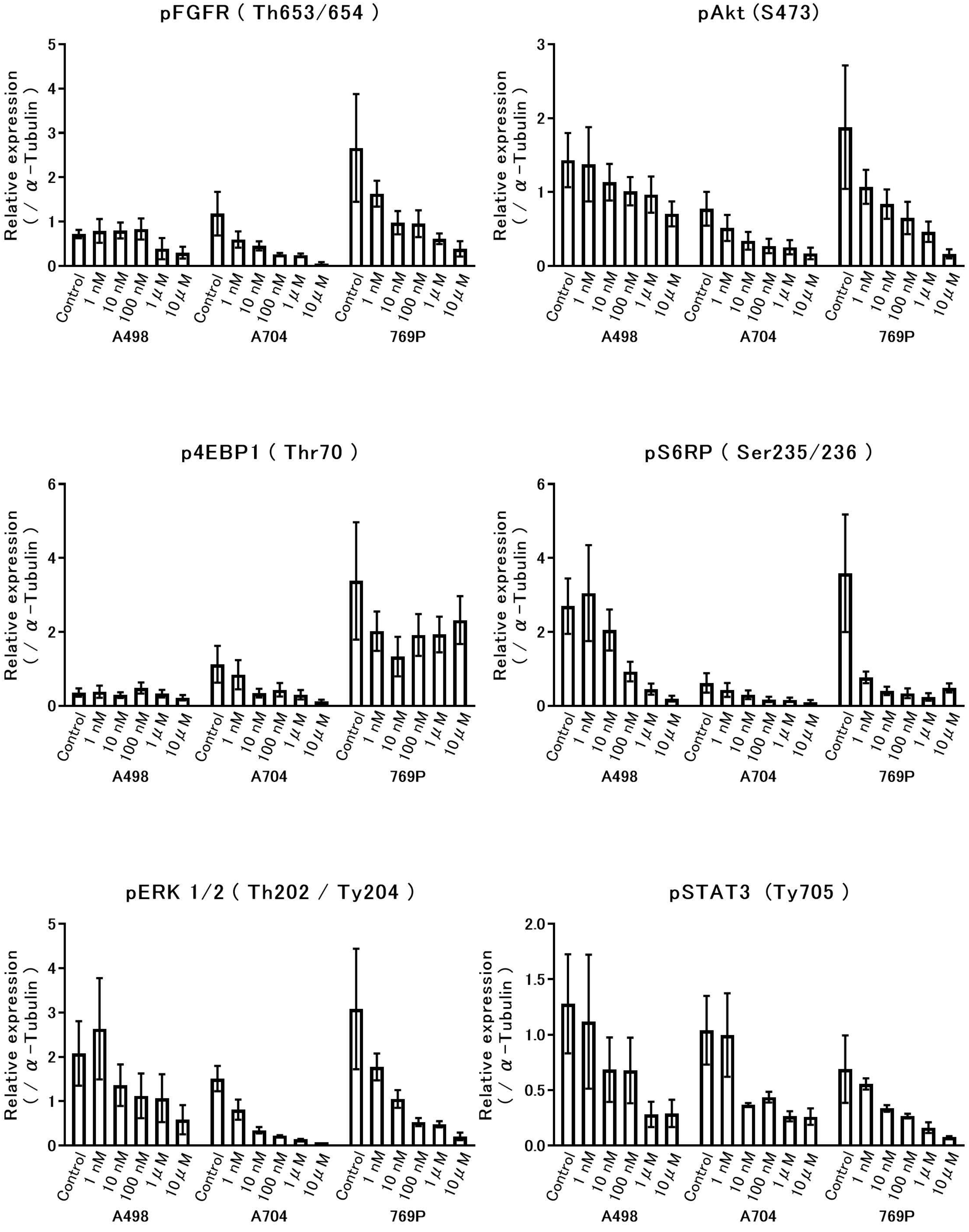

Figure S9. Densitometry analisys of western blot (Figure 4A).

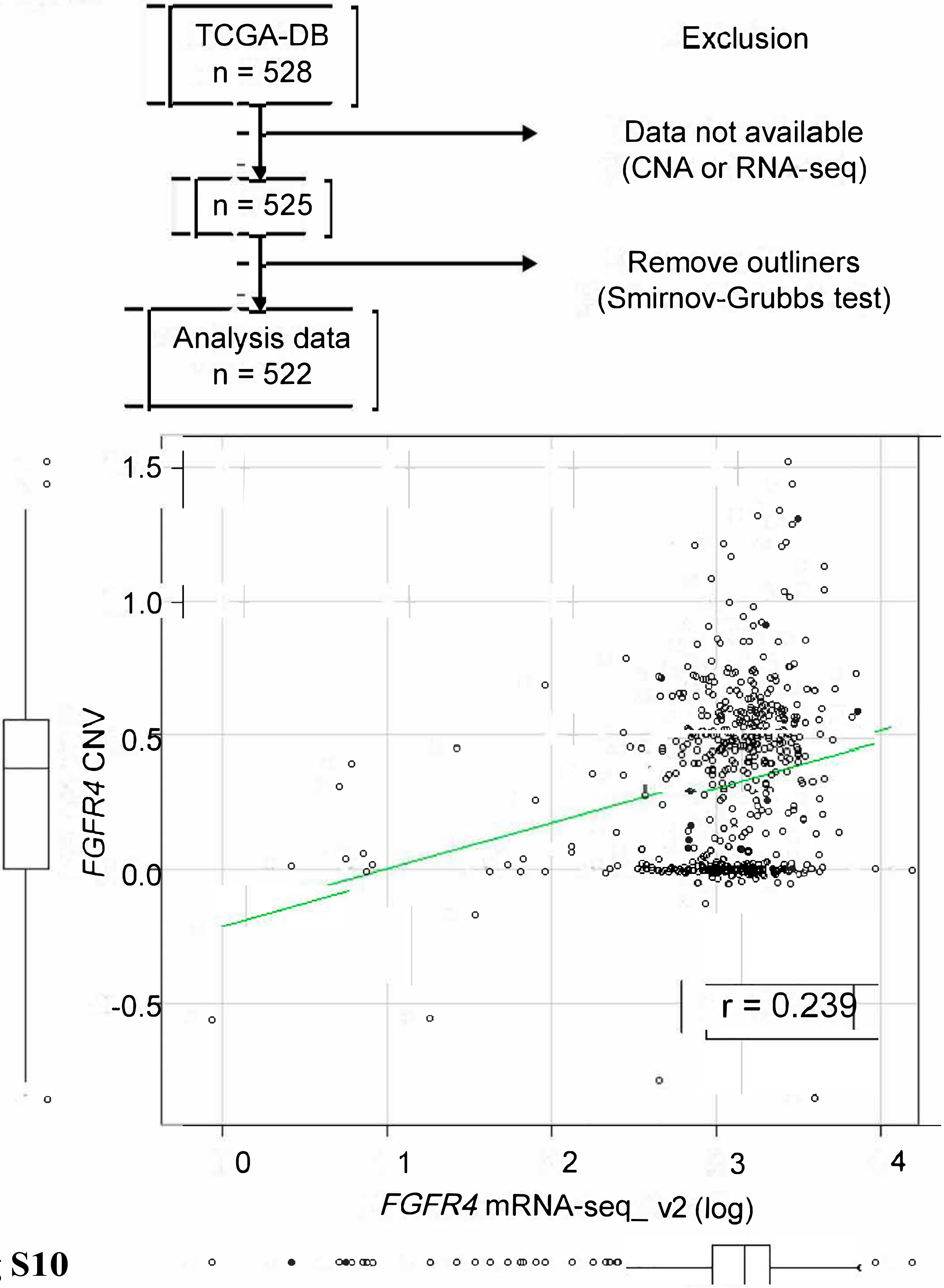

Fig S10

Analysis of the relationship between *FGFR4* gene copy number and *FGFR4* mRNA expression in TCGA database.

Correlation analysis was performed on FGFR4 CNV and FGFR4 mRNA-seq V2 data of 522 cases from TCGA database, excluding outliers for CNV (Smimov-Grubbs test). Normality was determined using the logai·itlunic value of RNAseqV2 data.
Pearson's f'actional test revealed a weak coirrelation (r = 0.239, P < 0.01).

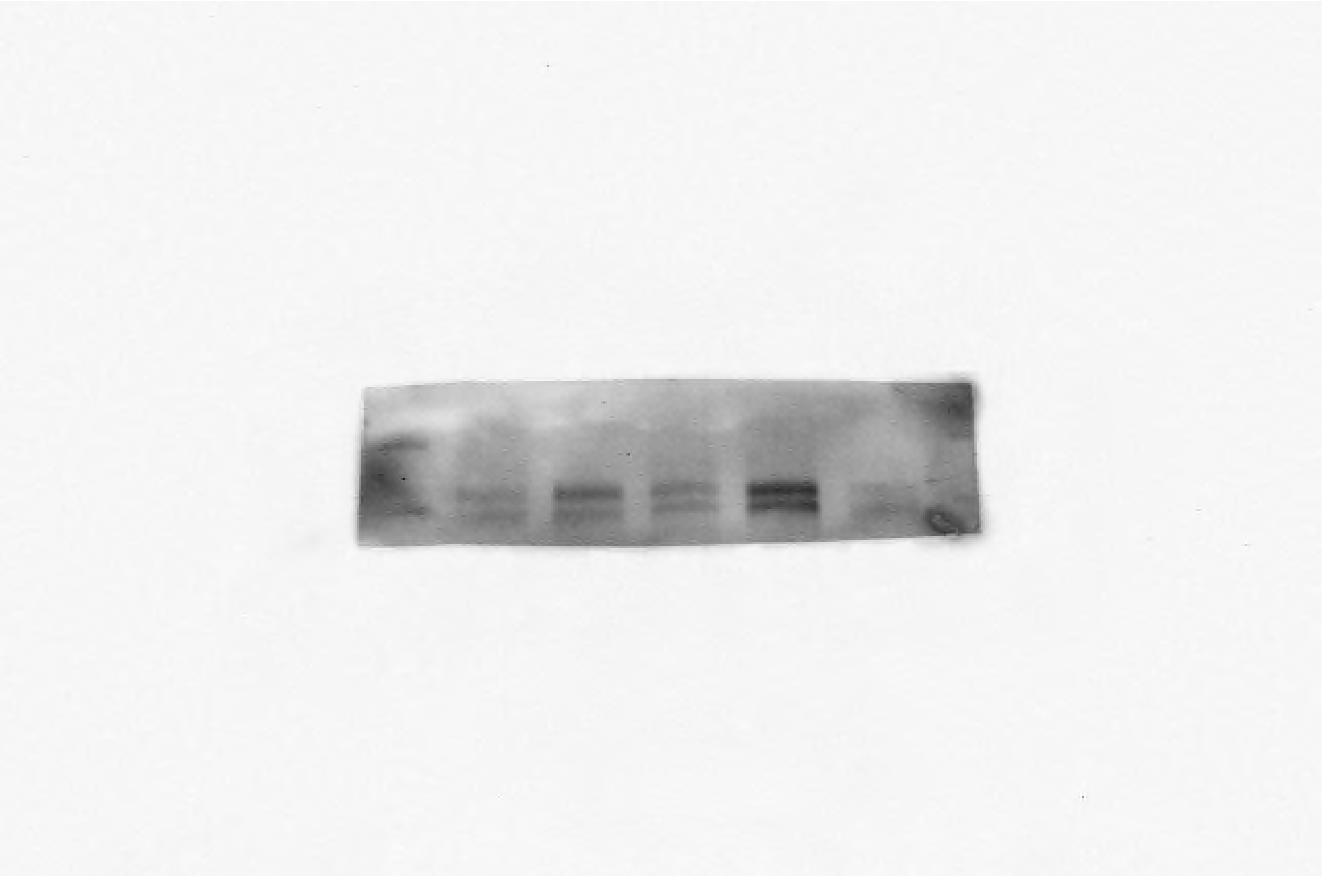

Figure S11. Western blot for FGFR4 protein expression in RCC cell lines.
From left, molecular weight marker, ACHN, A498, A704, 769-P, 786-O.
Gels were cut at 70 kDa and other side membrane was used in Western blots for other examination.

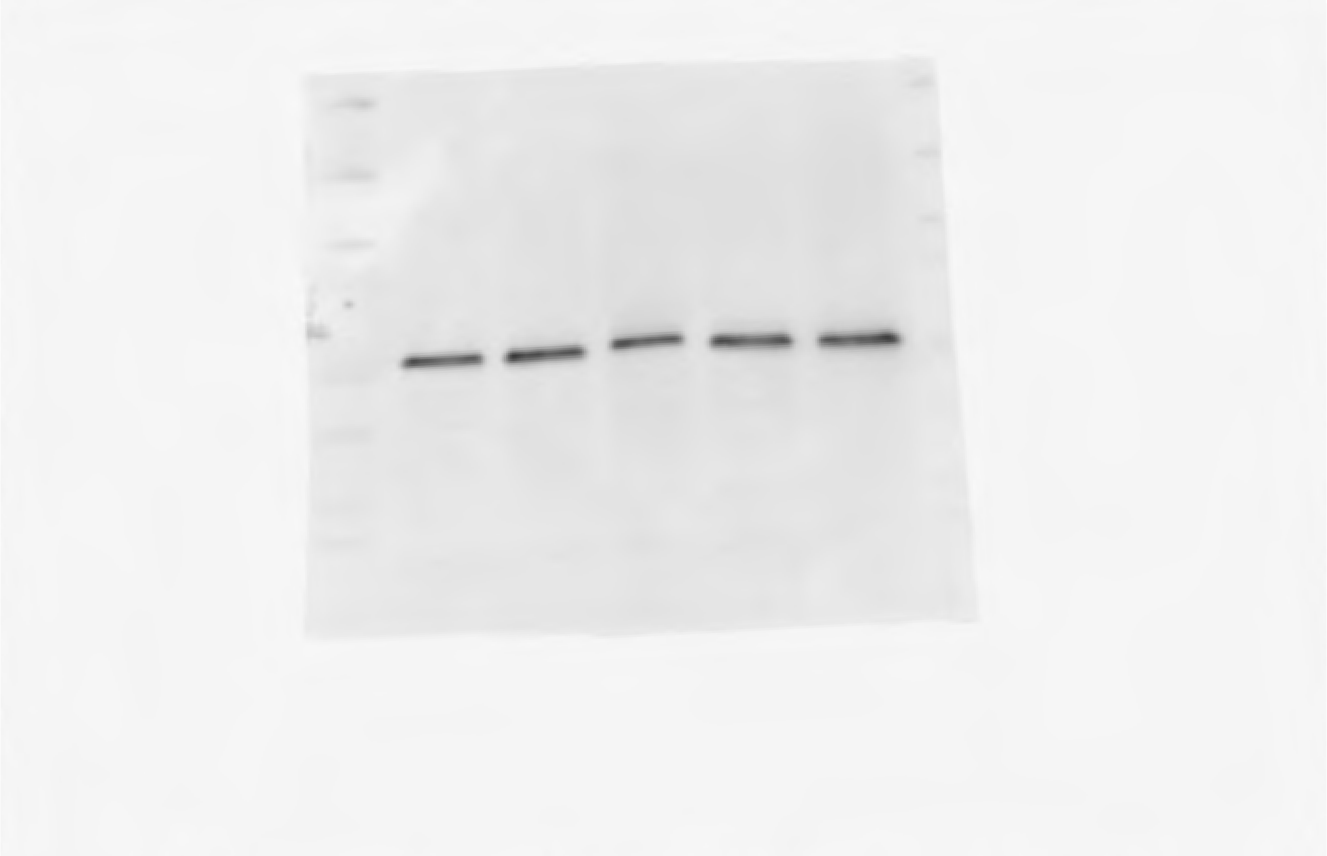

Figure S12. Western blot for α-Tubulin protein expression in RCC cell lines.
From left, molecular weight marker, ACHN, A498, A704, 769-P, 786-O.

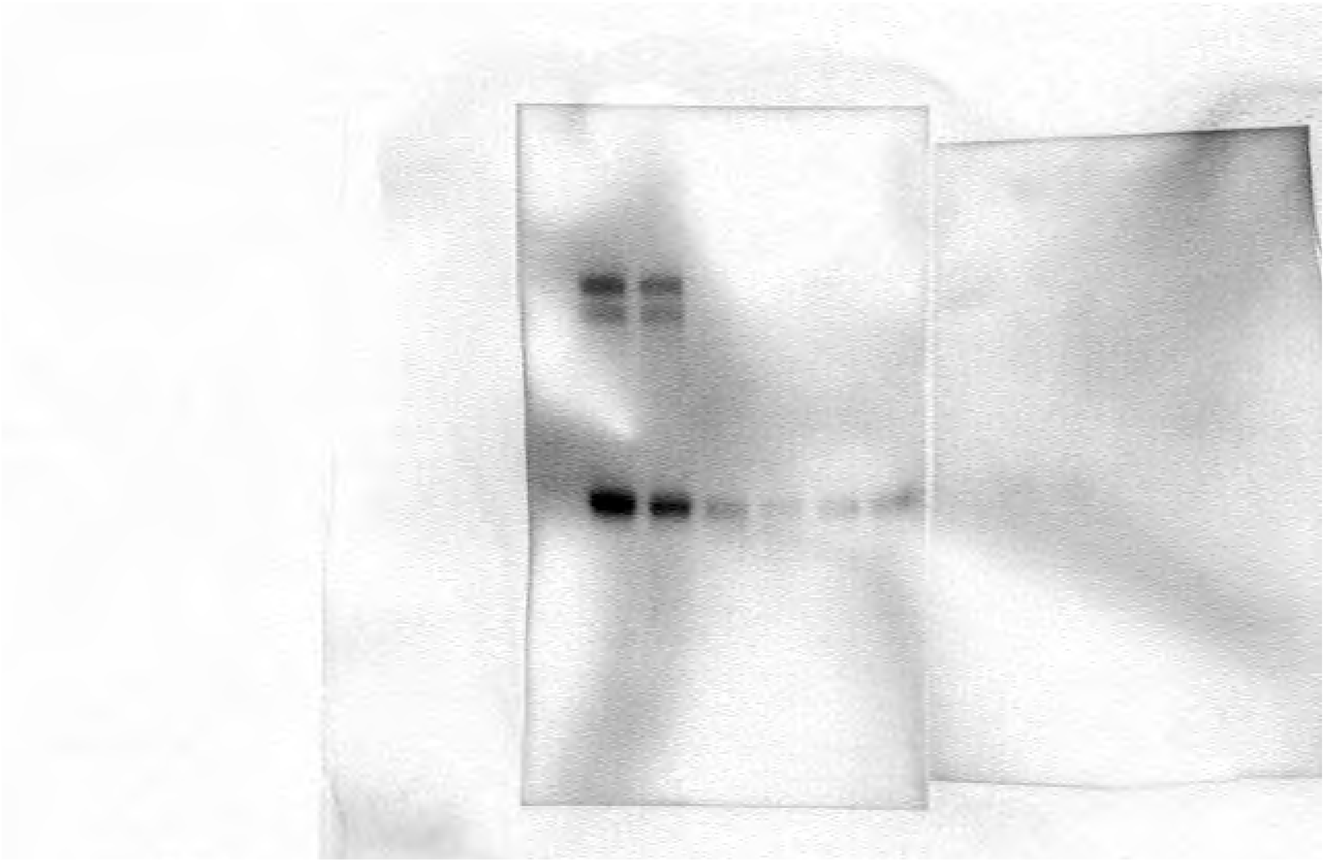

Figure S13. Western blot for FGFR4 protein expression in A498 cell line after siFGFR4 transfection, from left, Control (treatment free), Negative control, siFGFR4-1 (5 nM), siFGFR4-2 (5 nM), siFGFR4-1 (25 nM), siFGFR4-2 (25 nM). The right side menbrane was external control.

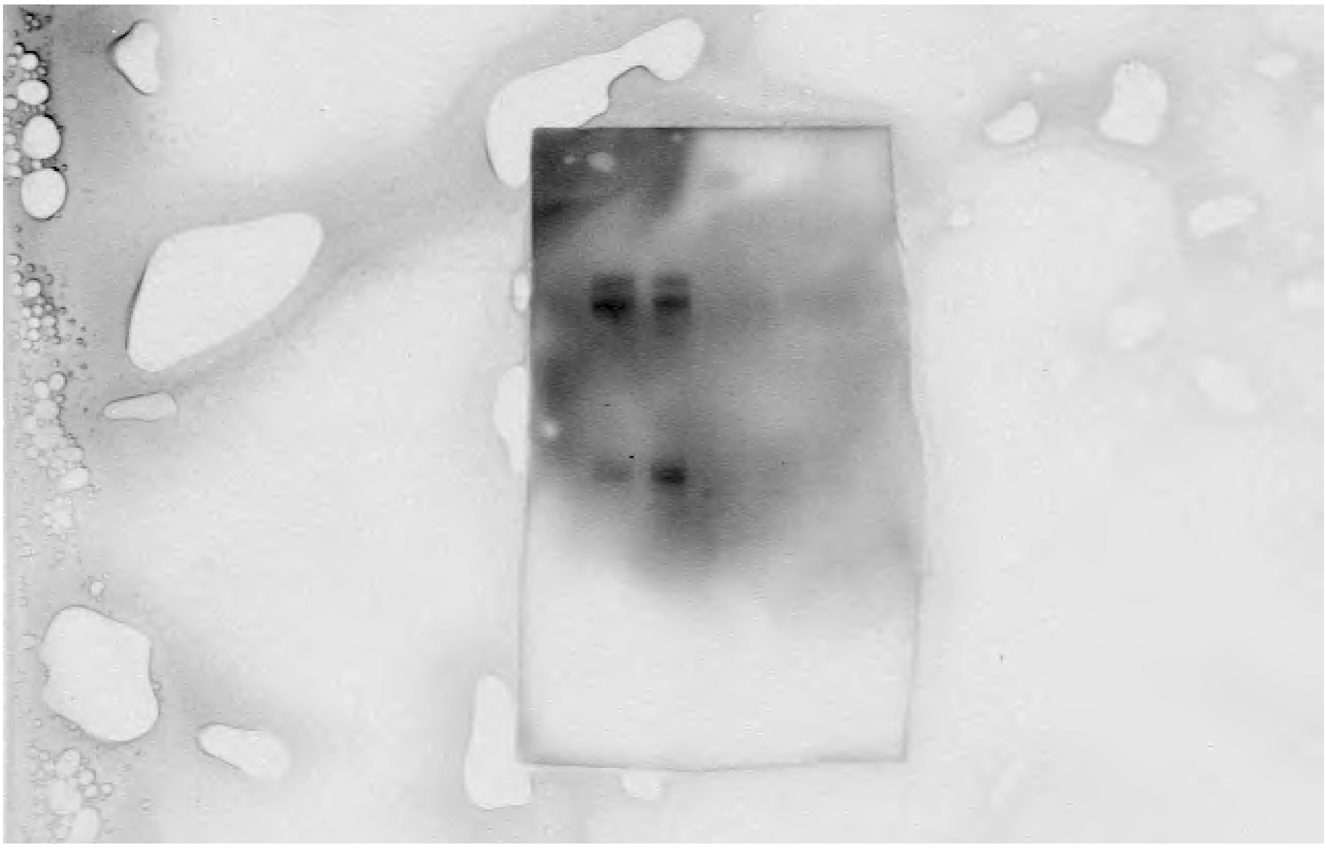

Figure S14 .Western blot for FGFR4 protein expression in A704 cell line after siFGFR4 transfection. From left (right gel) , Control (treatment free), Negative control, siFGFR4-1 (5 nM), siFGFR4-2 (5 nM), siFGFR4-1 (25 nM), siFGFR4-2 (25 nM).

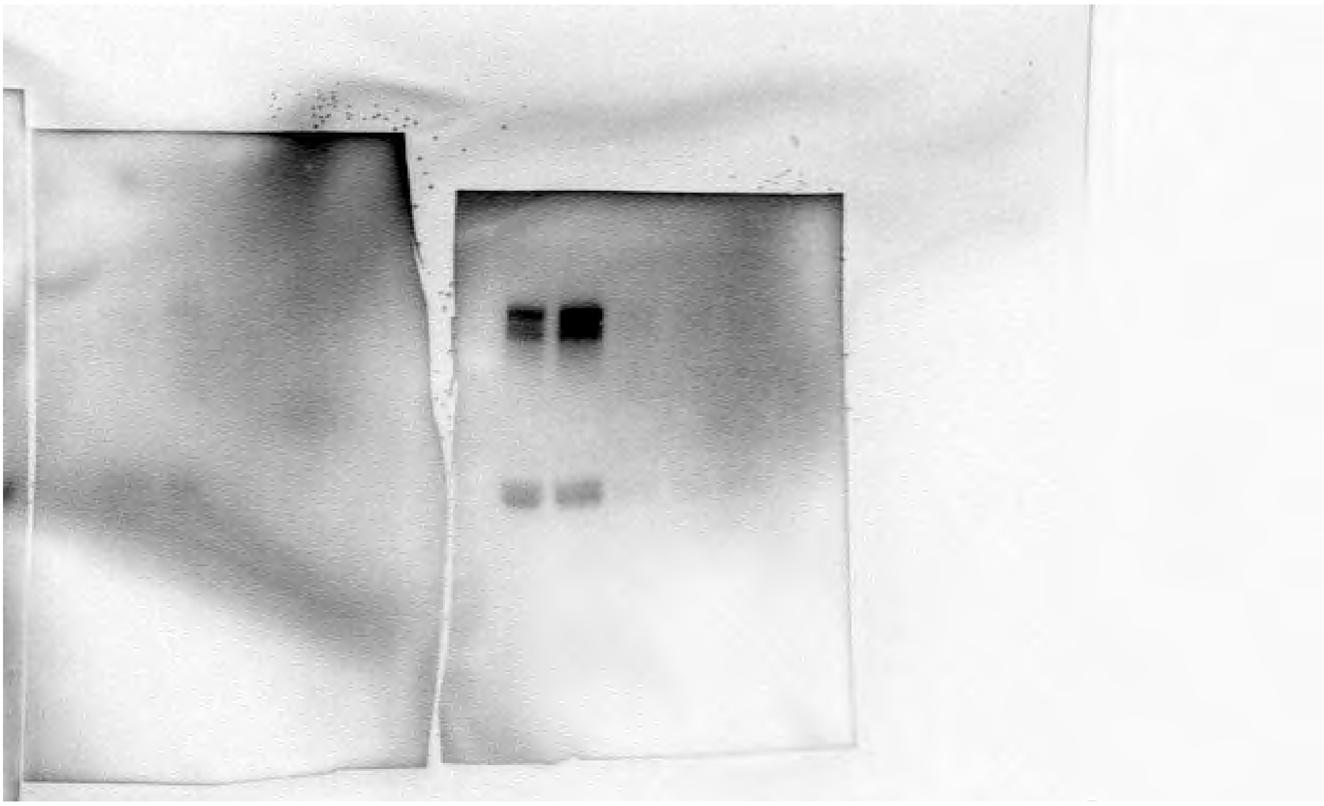

Figure S15 Western blot for FGFR4 protein expression in 769P cell line after siFGFR4 transfection. From left (right menbrane) , Control (treatment free), Negative control, siFGFR4-1 (5 nM), siFGFR4-2 (5 nM), siFGFR4-1 (25 nM), siFGFR4-2 (25 nM). the left side membrane was external control.

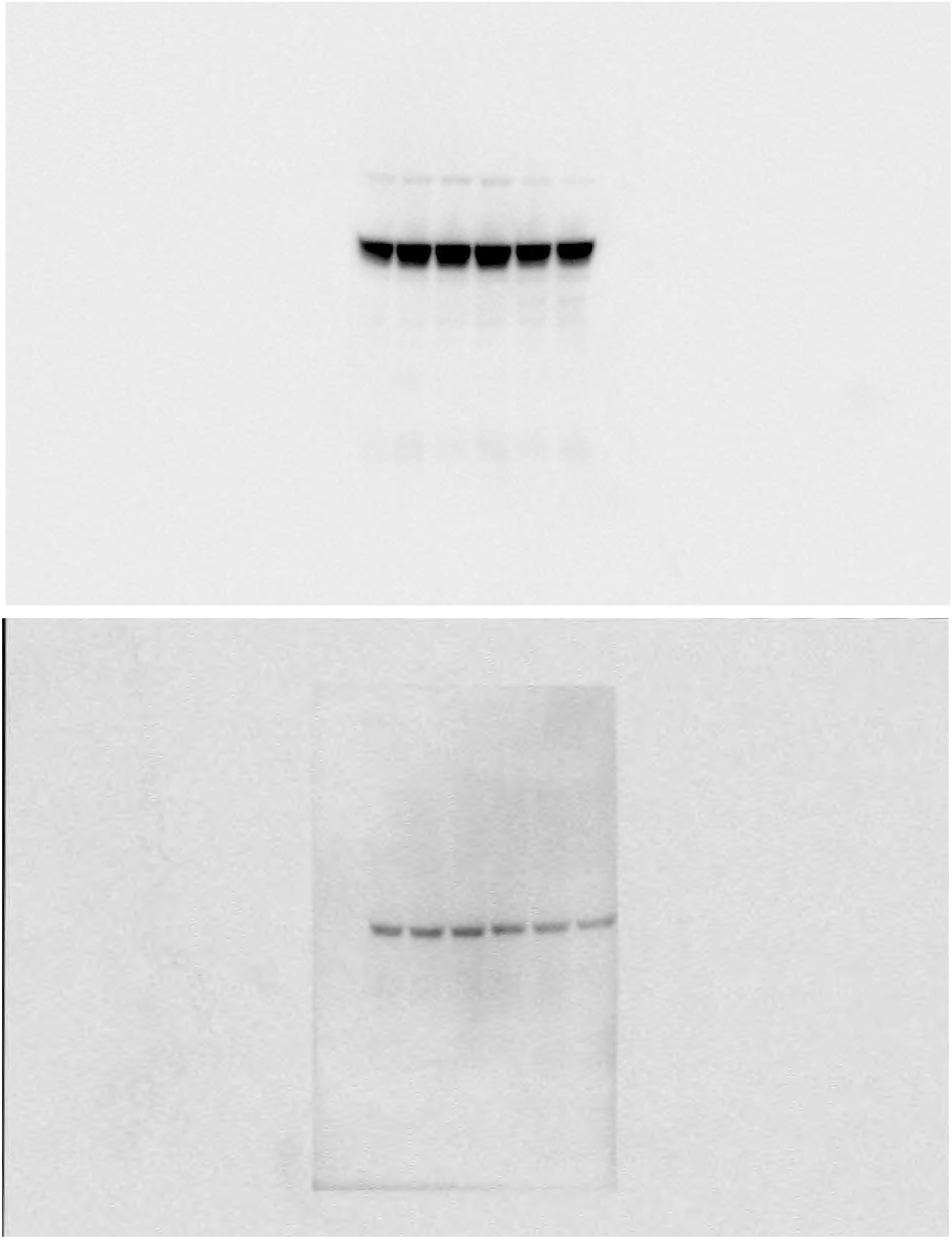

Figure S16 Western blot for AKT (Upper gel) and Phospho-AKT (Ser473, lower gel) protein expression in A498 cell line after siFGFR4 transfection. From left, Control (treatment free), Negative control, siFGFR4-1 (5 nM), siFGFR4-2 (5 nM), siFGFR4-1 (25 nM), siFGFR4-2 (25 nM).

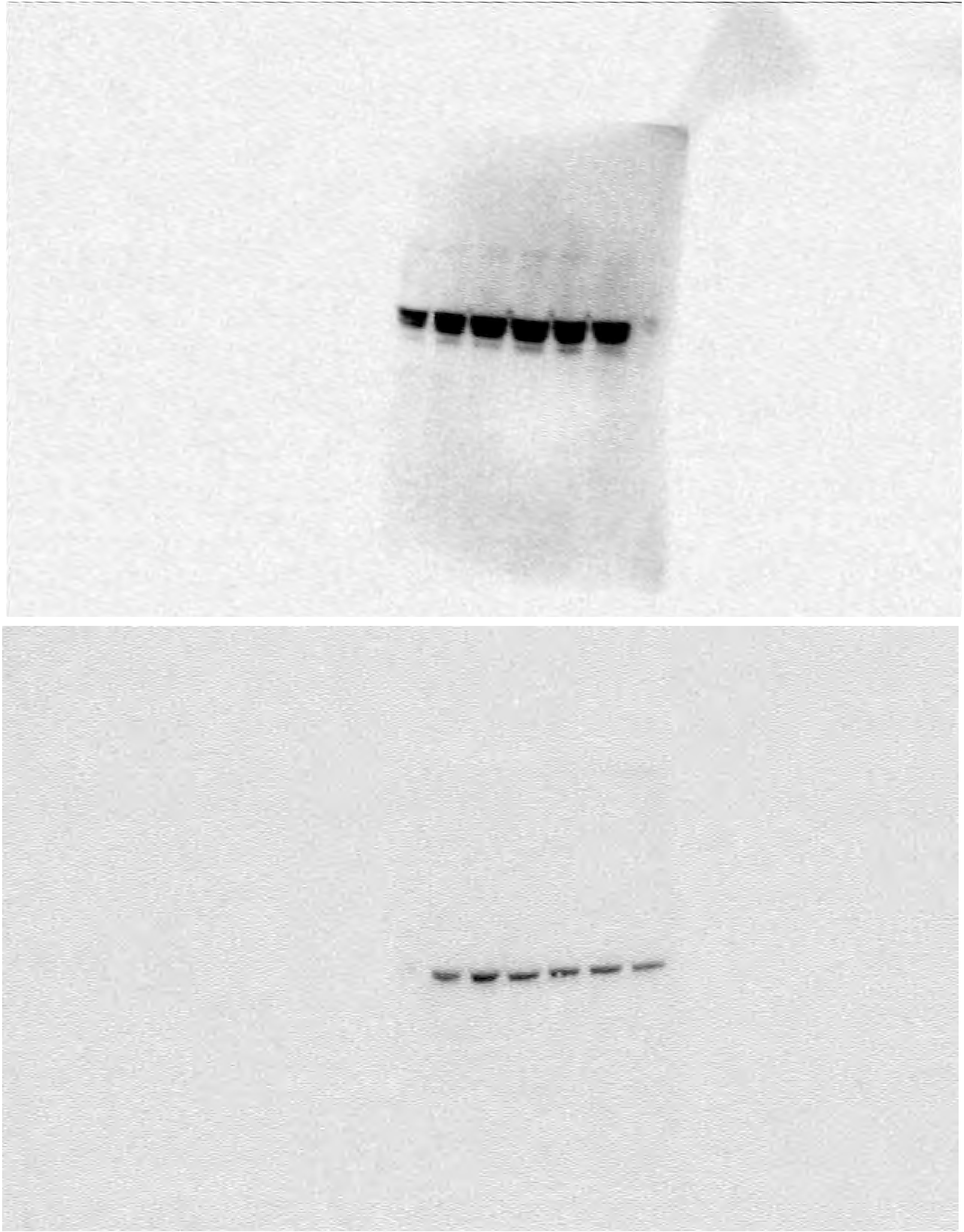

Figure S17 Western blot for AKT (Upper gel) and Phospho-AKT (Ser473, lower gel) protein expression in A704 cell line after siFGFR4 transfection. From left, Control (treatment free), Negative control, siFGFR4-1 (5 nM), siFGFR4-2 (5 nM), siFGFR4-1 (25 nM), siFGFR4-2 (25 nM).

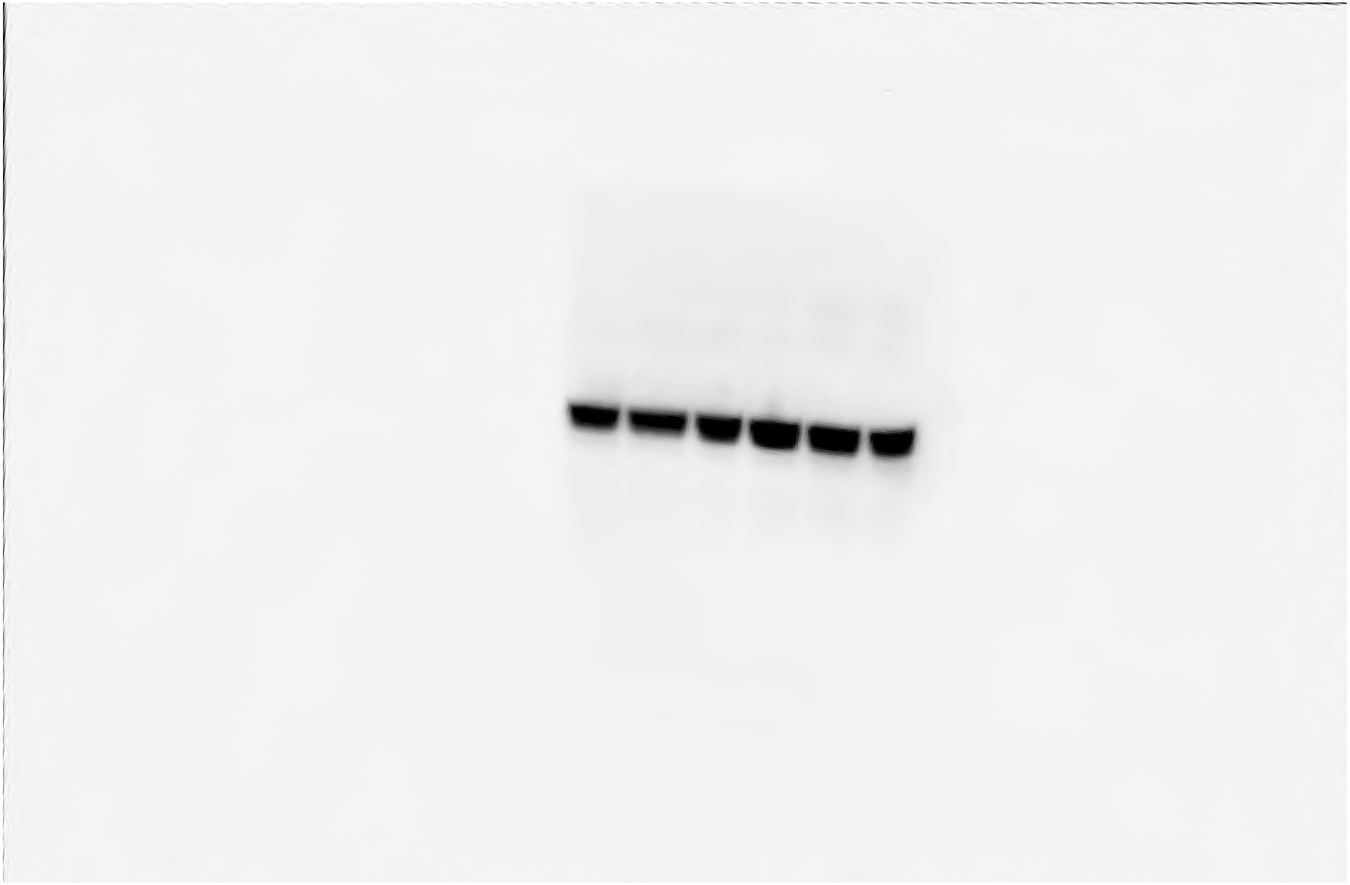

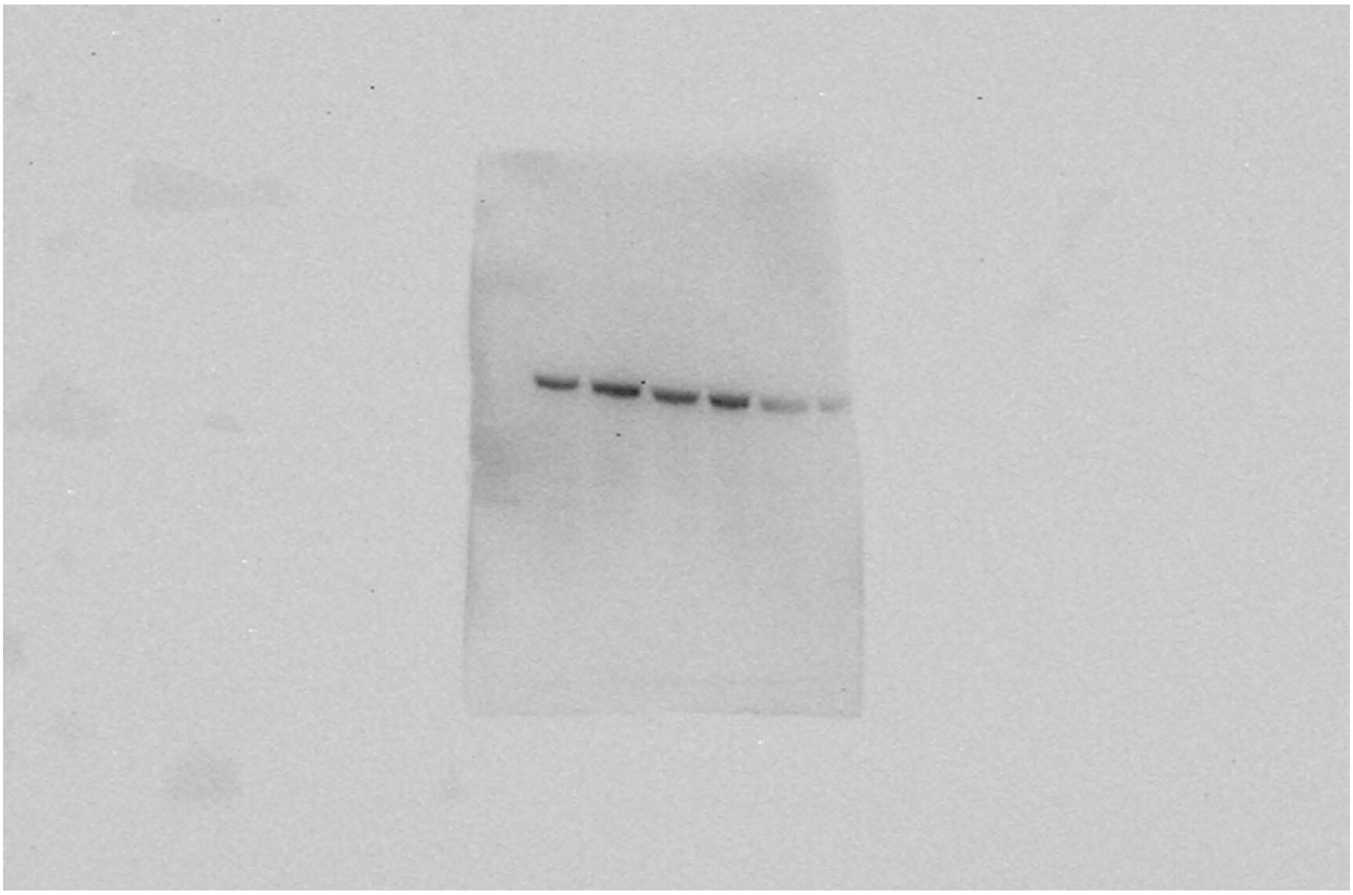

Figure S18 Western blot for AKT (Upper gel) and Phospho-AKT (Ser473, lower gel) protein expression in 769P cell line after siFGFR4 transfection. From left, Control (treatment free), Negative control, siFGFR4-1 (5 nM), siFGFR4-2 (5 nM), siFGFR4-1 (25 nM), siFGFR4-2 (25 nM).

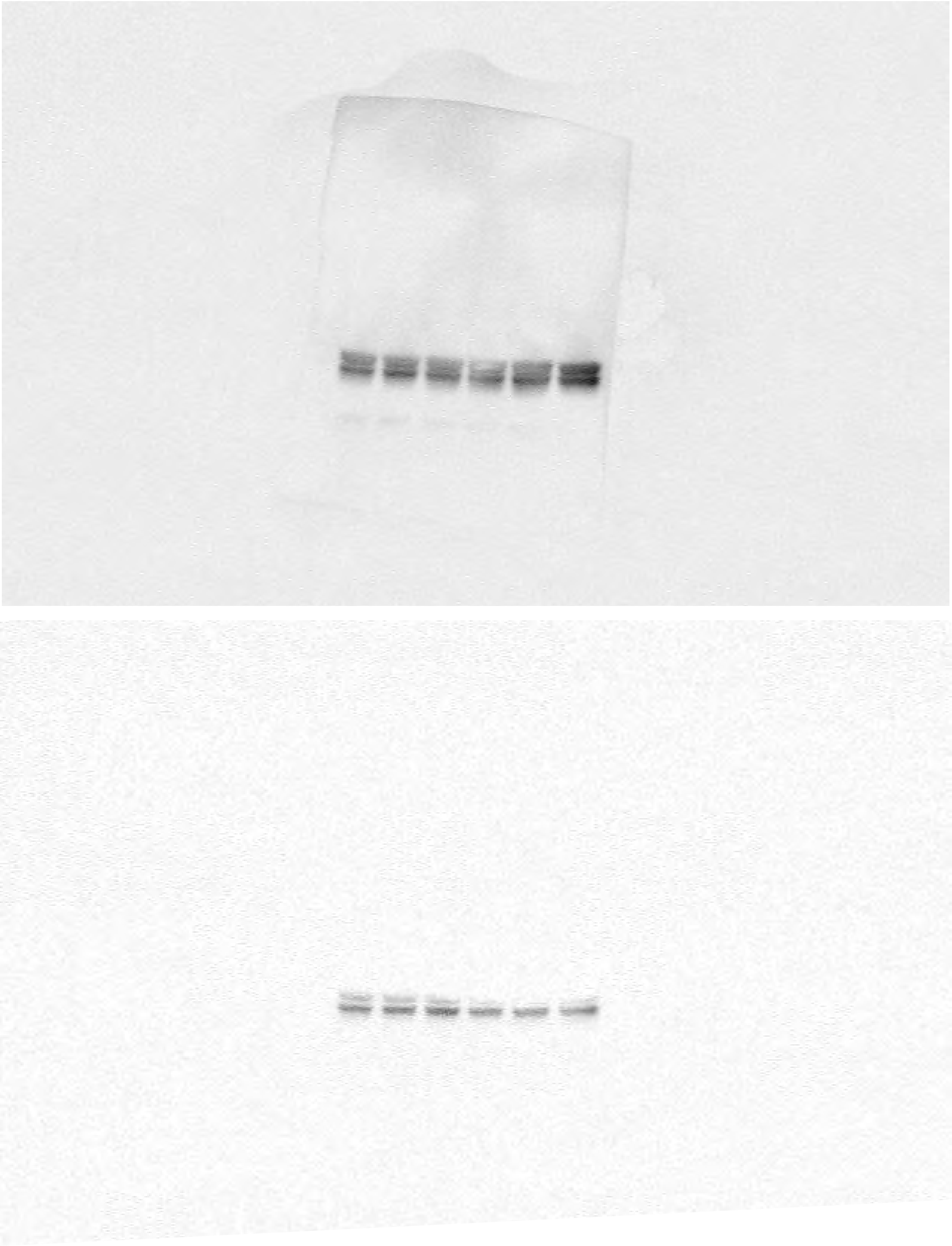

Figure S19 Western blot for ERK1/2 (Upper gel) and Phospho-ERK1/2 (Thr202/Tyr204, lower gel) protein expression in A498 cell line after siFGFR4 transfection. From left, Control (treatment free), Negative control, siFGFR4-1 (5 nM), siFGFR4-2 (5 nM), siFGFR4-1 (25 nM), siFGFR4-2 (25 nM).

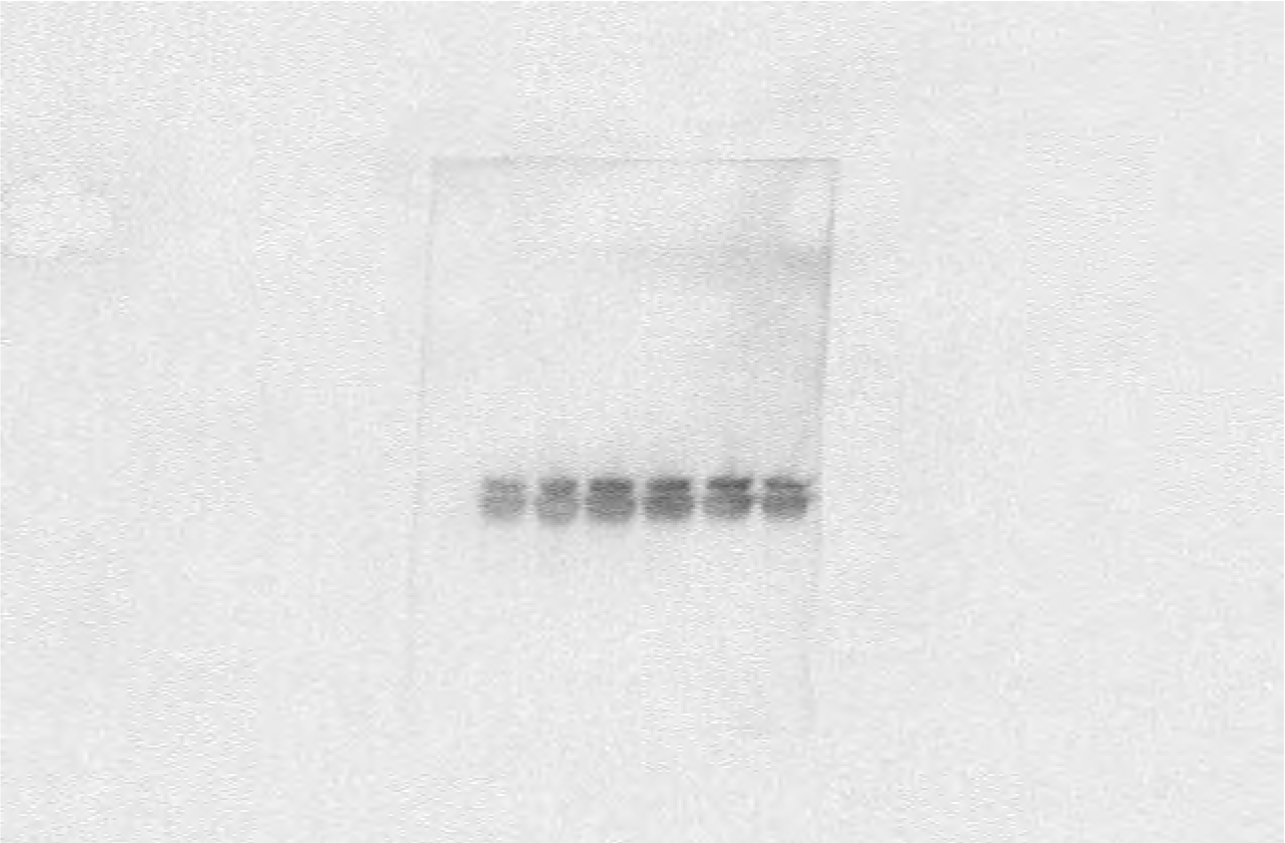

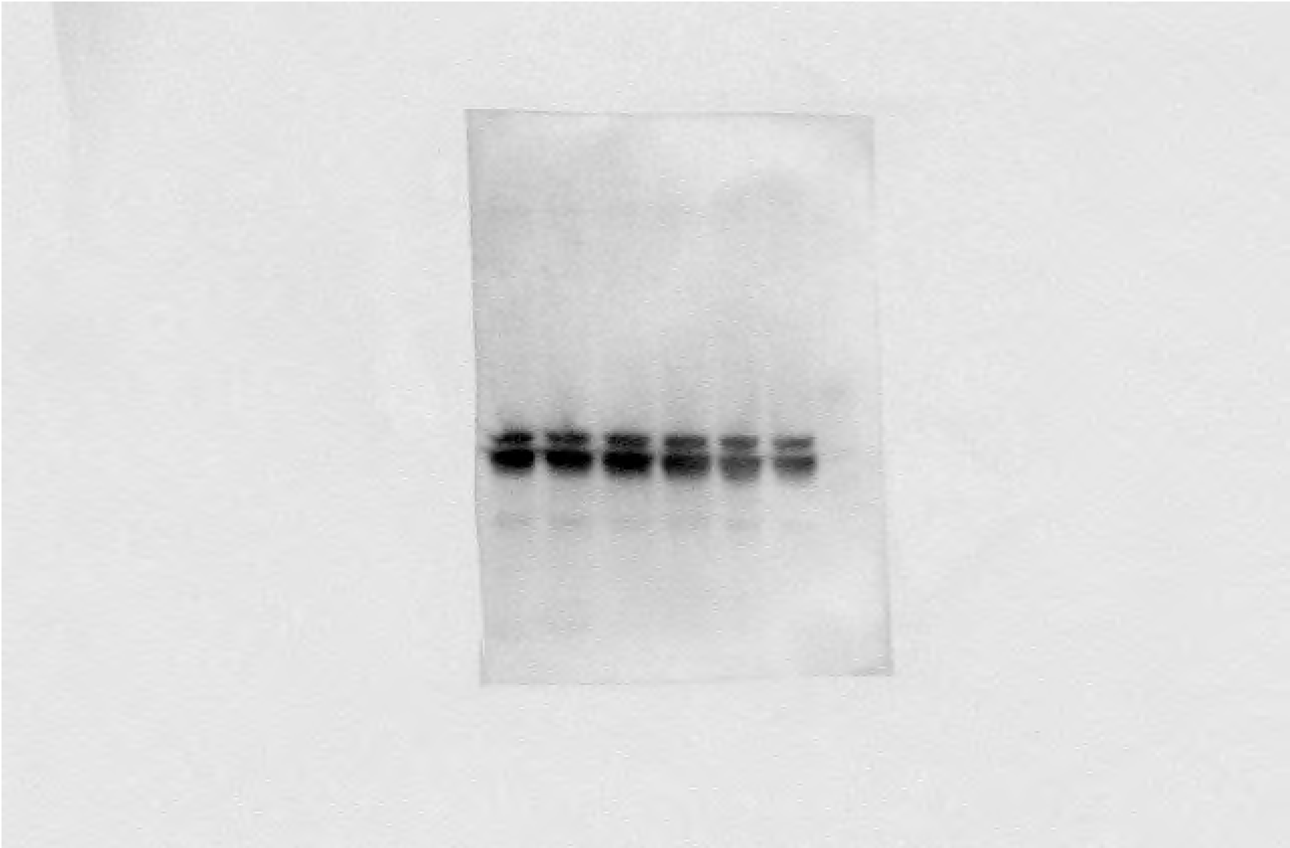

Figure S20 Western blot for ERK1/2 (Upper gel) and Phospho-ERK1/2 (Thr202/Tyr204, lower gel) protein expression in A704 cell line after siFGFR4 transfection. From left, Control (treatment free), Negative control, siFGFR4-1 (5 nM), siFGFR4-2 (5 nM), siFGFR4-1 (25 nM), siFGFR4-2 (25 nM).

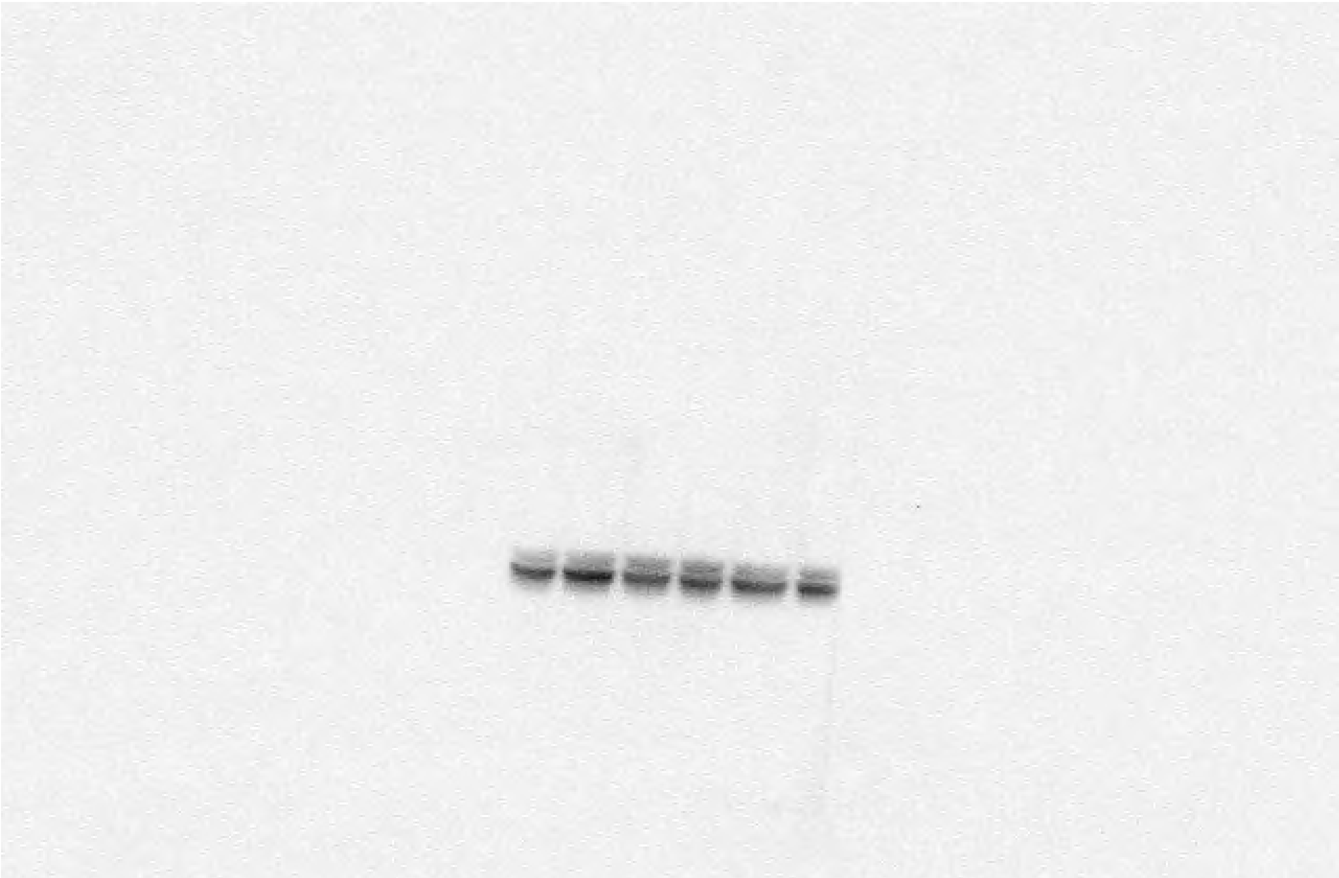

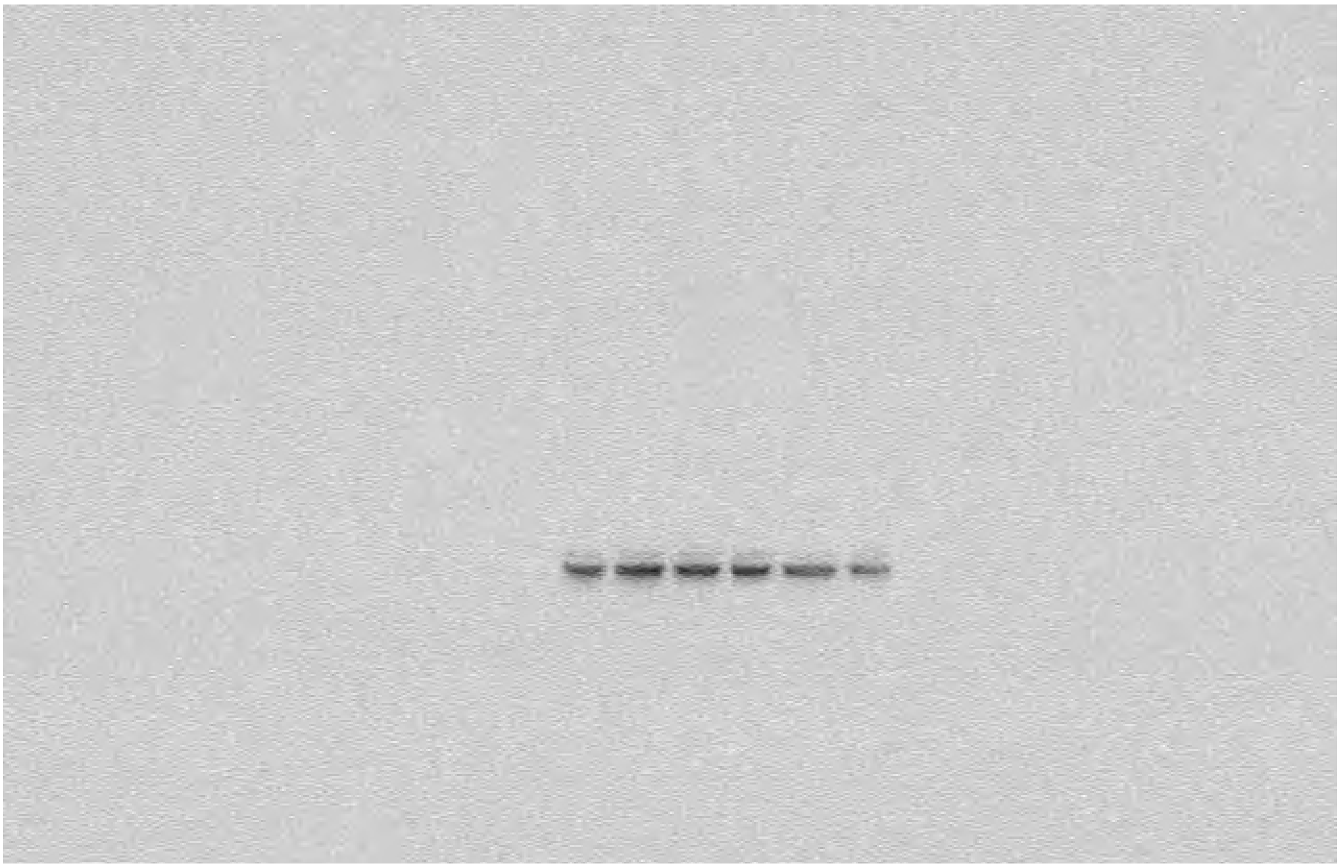

Figure S21

Western blot for ERK1/2 (Upper gel) and Phospho-ERK1/2 (Thr202/Tyr204, lower gel) protein expression in 769P cell line after siFGFR4 transfection. From left, Control (treatment free), Negative control, siFGFR4-1 (5 nM), siFGFR4-2 (5 nM), siFGFR4-1 (25 nM), siFGFR4-2 (25 nM).

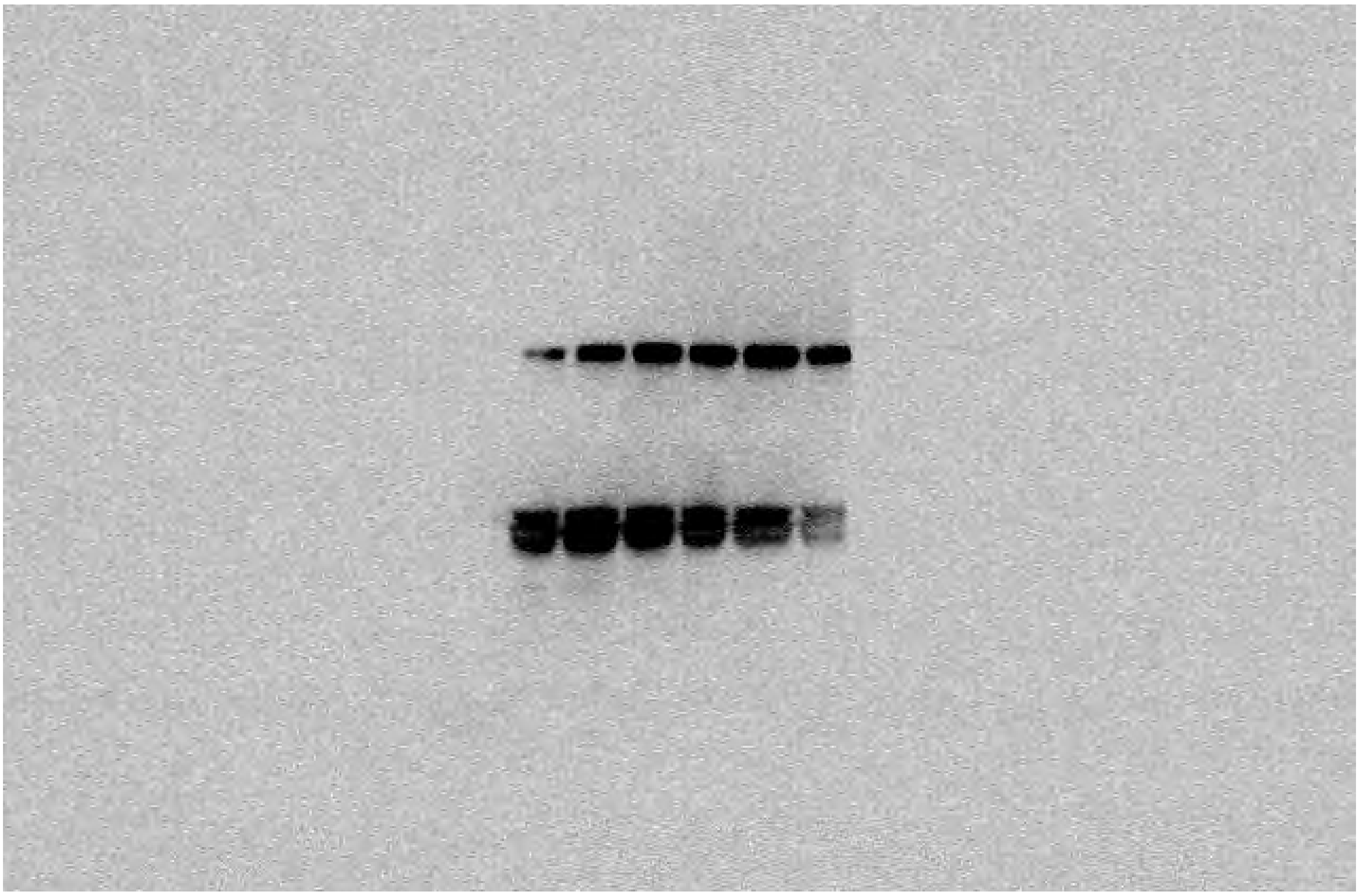

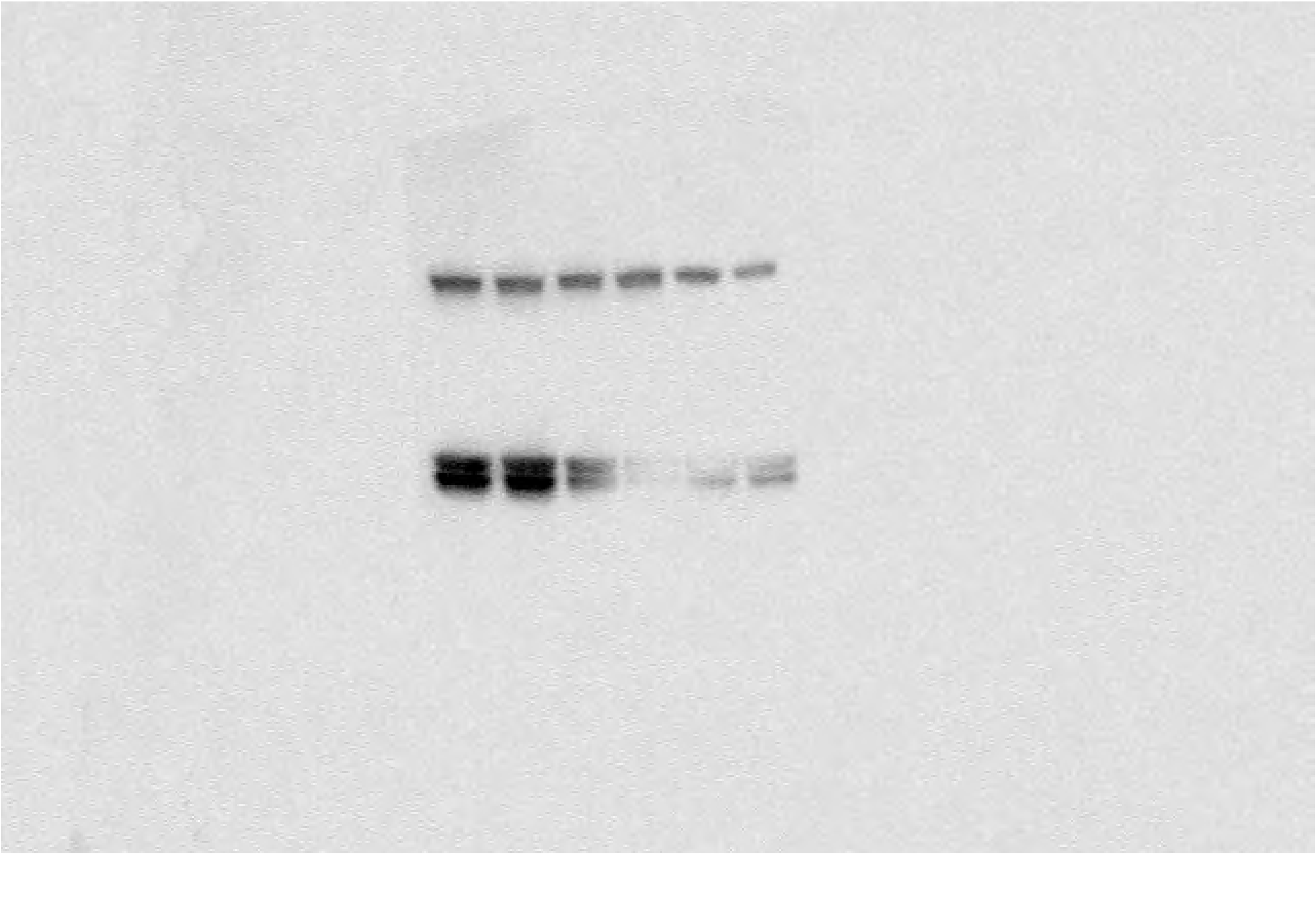

Figure S22

Western blot for STAT3 (Upper gel) and Phospho-STAT3 (Tyr705, lower gel) protein expression in A498 cell line after siFGFR4 transfection. From left, Control (treatment free), Negative control, siFGFR4-1 (5 nM), siFGFR4-2 (5 nM), siFGFR4-1 (25 nM), siFGFR4-2 (25 nM). The lower band of each gel is a remnant of Phosho-ERK1/2 experiment.

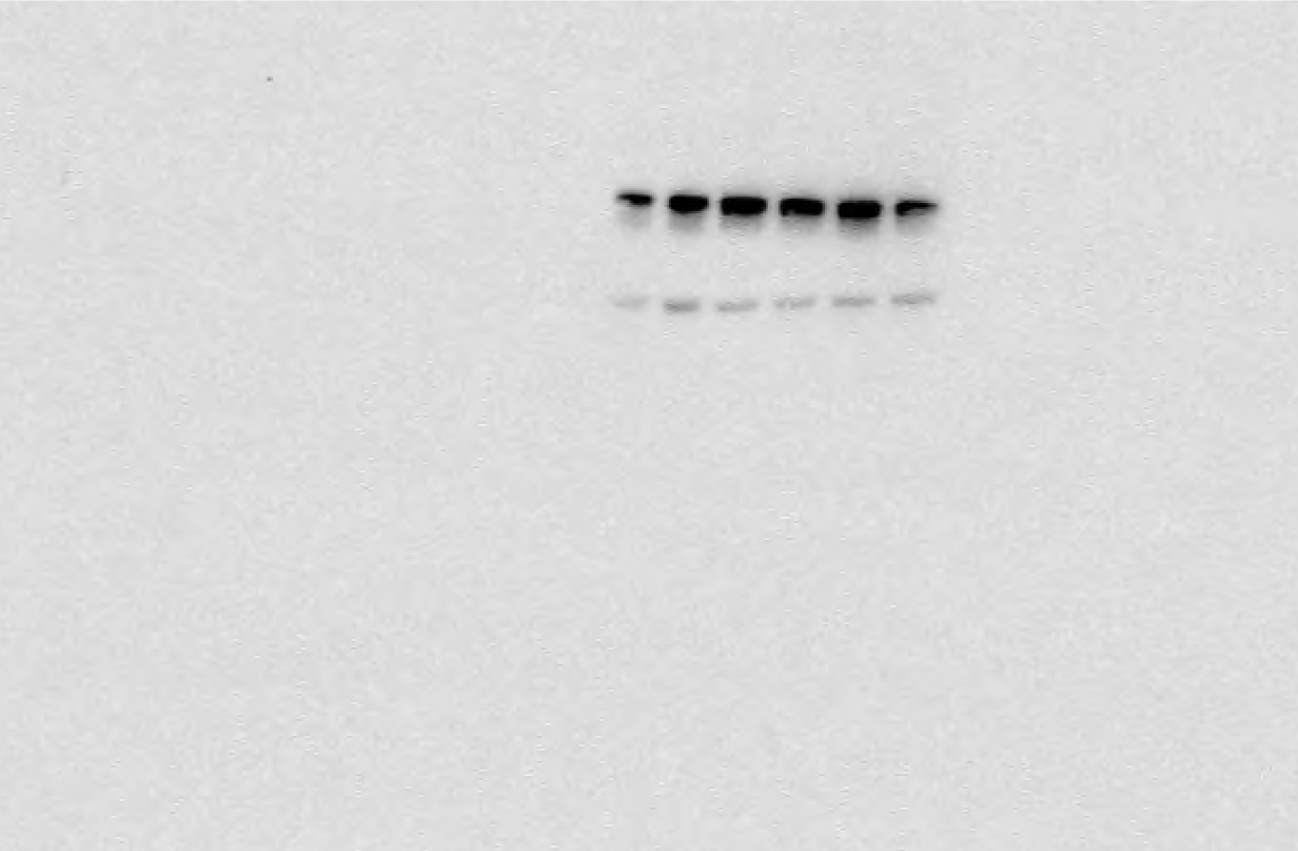

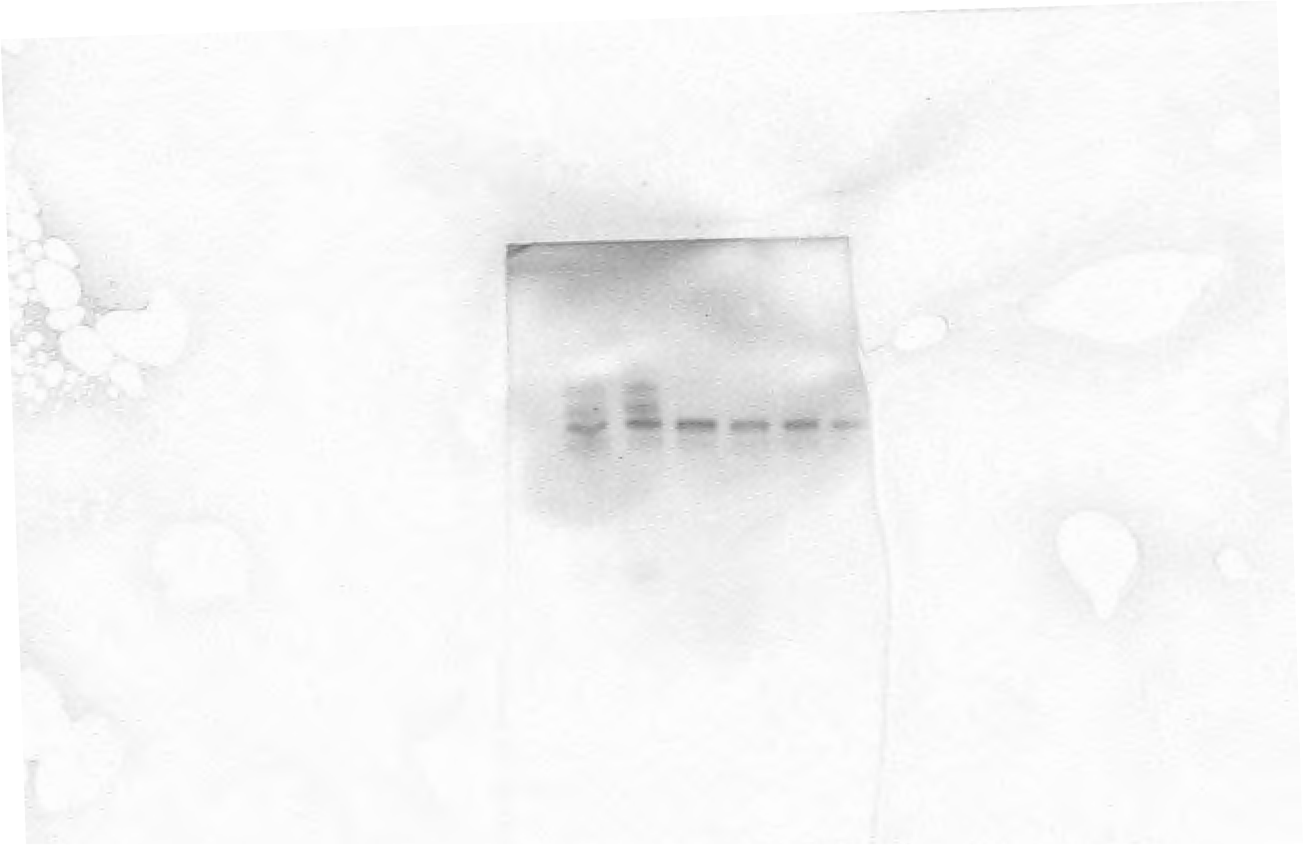

Figure S23 Western blot for STAT3 (Upper gel) and Phospho-STAT3 (Tyr705, lower gel) protein expression in A704 cell line after siFGFR4 transfection. From left, Control (treatment free), Negative control, siFGFR4-1 (5 nM), siFGFR4-2 (5 nM), siFGFR4-1 (25 nM), siFGFR4-2 (25 nM).

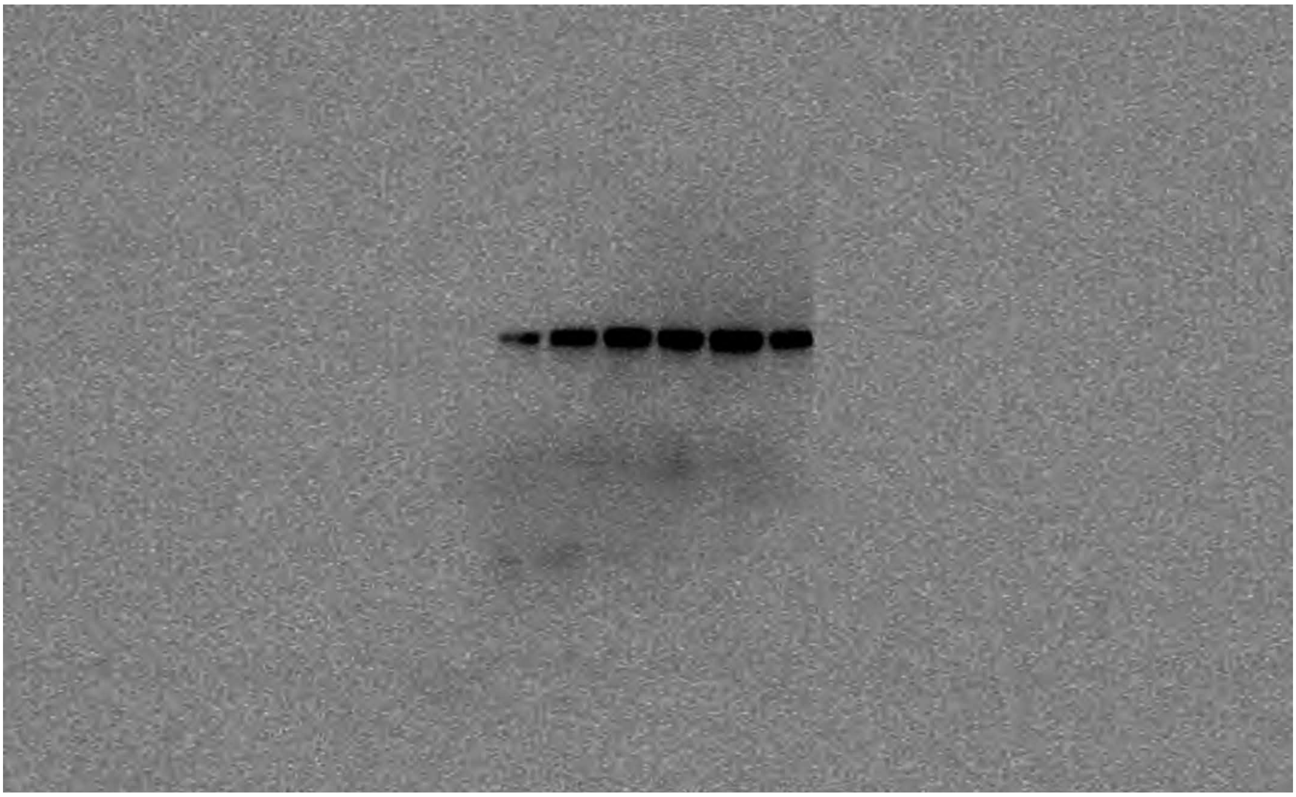

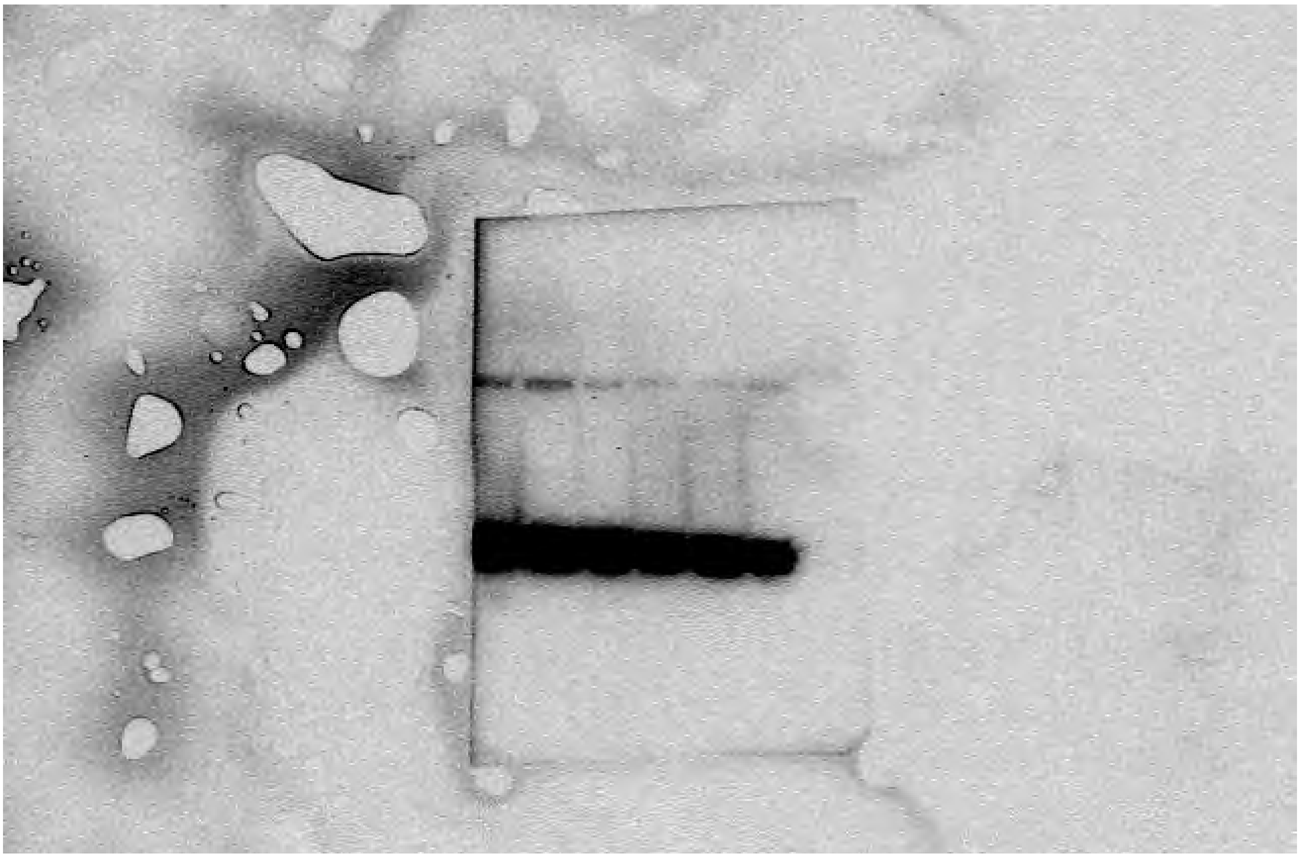

Figure S24 Western blot for STAT3 (Upper gel) and Phospho-STAT3 (Tyr705, lower gel) protein expression in 769P cell line after siFGFR4 transfection. From left, Control (treatment free), Negative control, siFGFR4-1 (5 nM), siFGFR4-2 (5 nM), siFGFR4-1 (25 nM), siFGFR4-2 (25 nM).

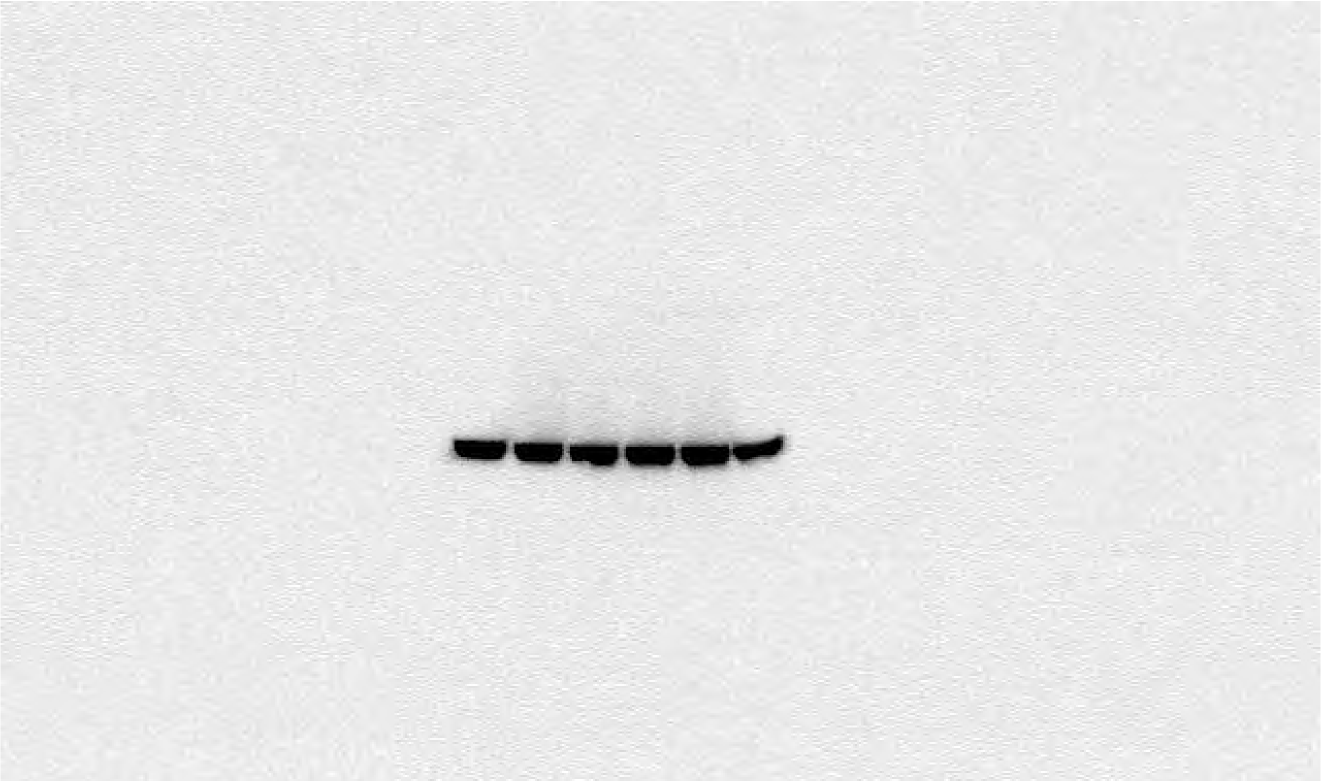

Figure S25 Western blot for α-Tubulin expression of A498 cells after siFGFR4 transfection.

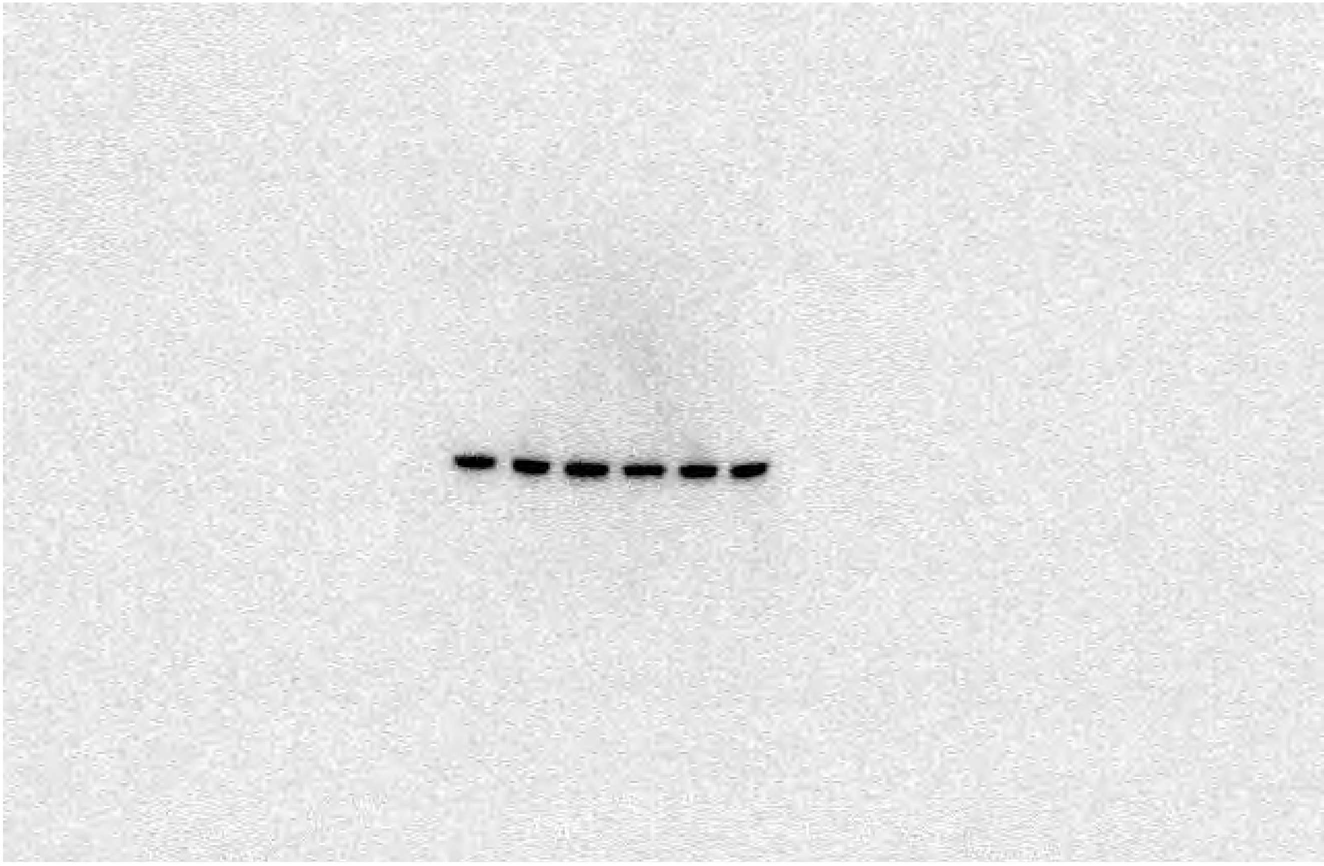

Figure S26 Western blot for α-Tubulin expression of A704 cells after siFGFR4 transfection.

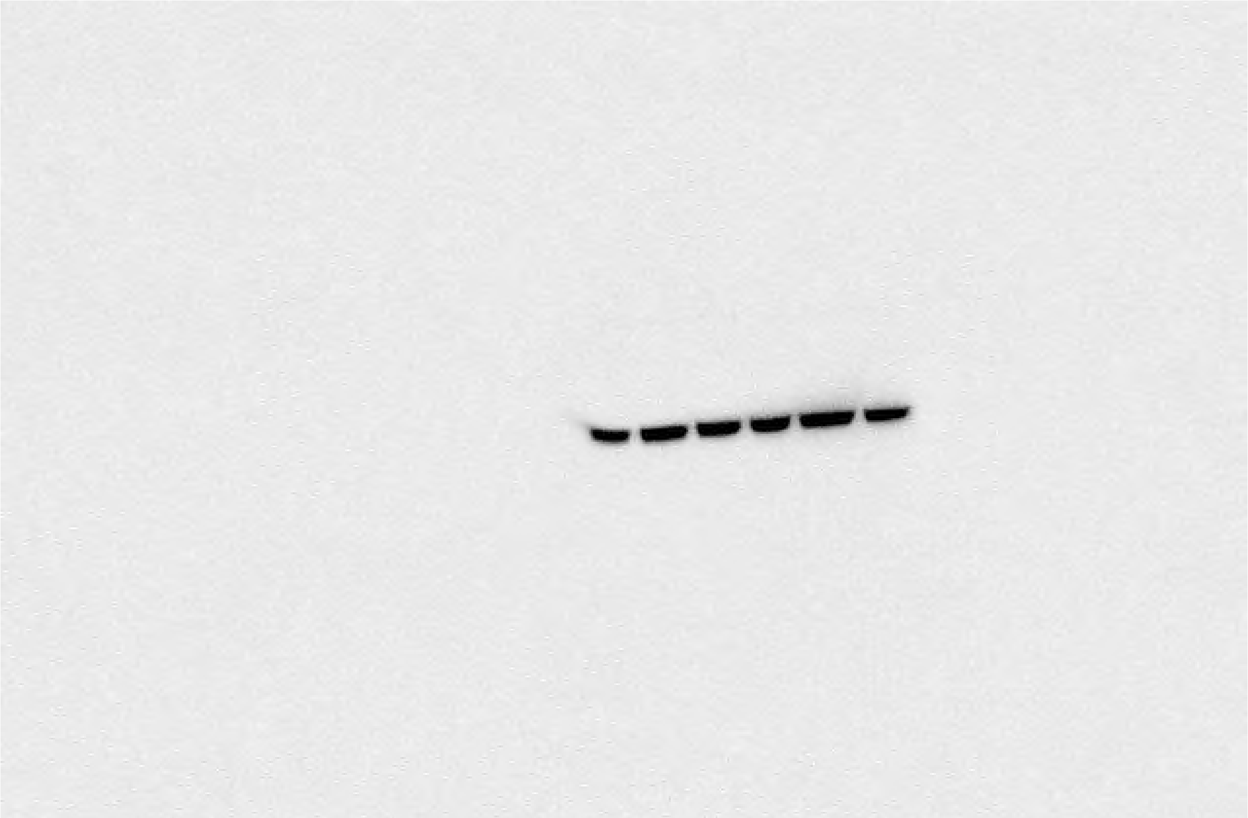

Figure S27 Western blot for α-Tubulin expression of 769P cells after siFGFR4 transfection.

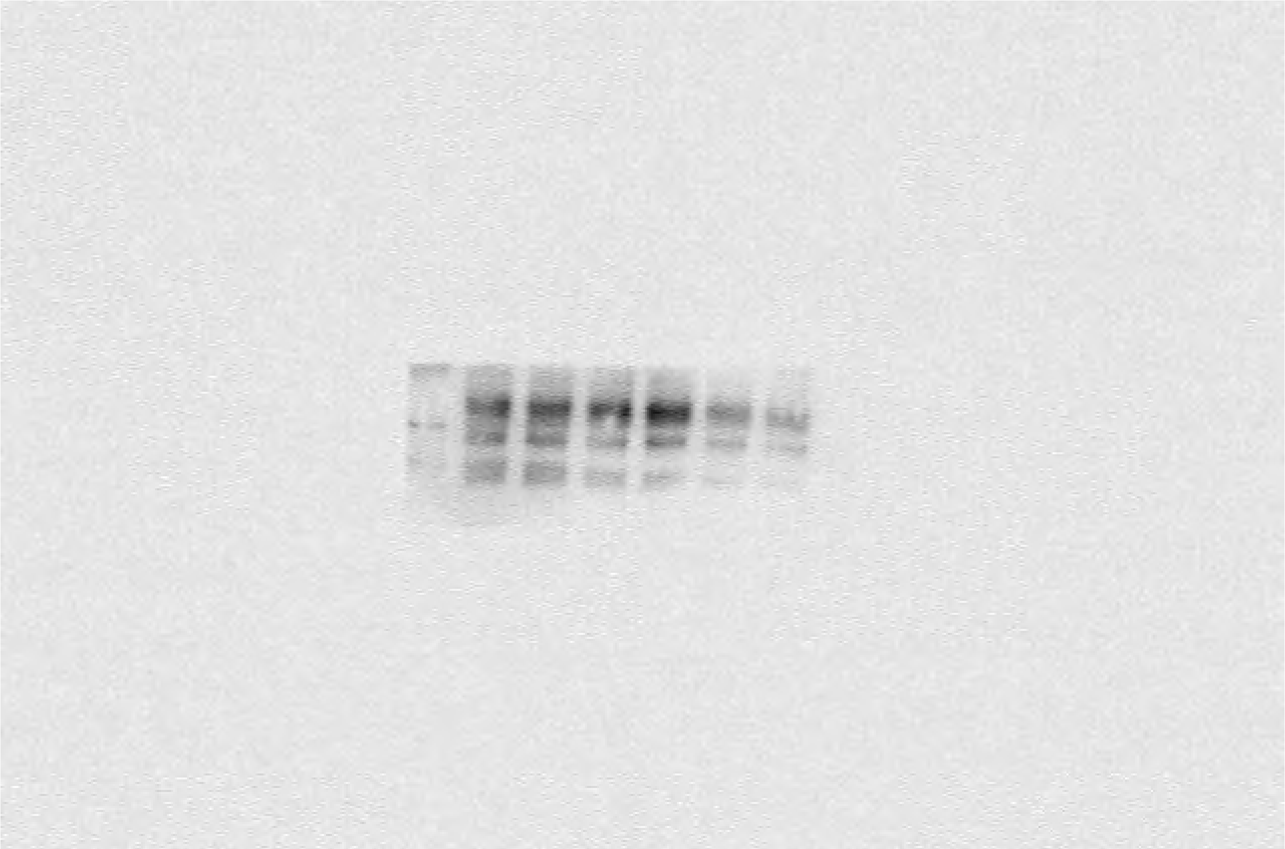

Figure S28 Western blot for phospho-FGFR (Thr653/654) of A498 cell line after BLU9931 treatment. From left, MW marker, Control, 1 nM, 10 nM, 100 nM, 1 μM, 10 μM of BLU9931 treatment concentration. Gel were cut at 70 kDa and other side membrane was used for Western blotting for other examinations.

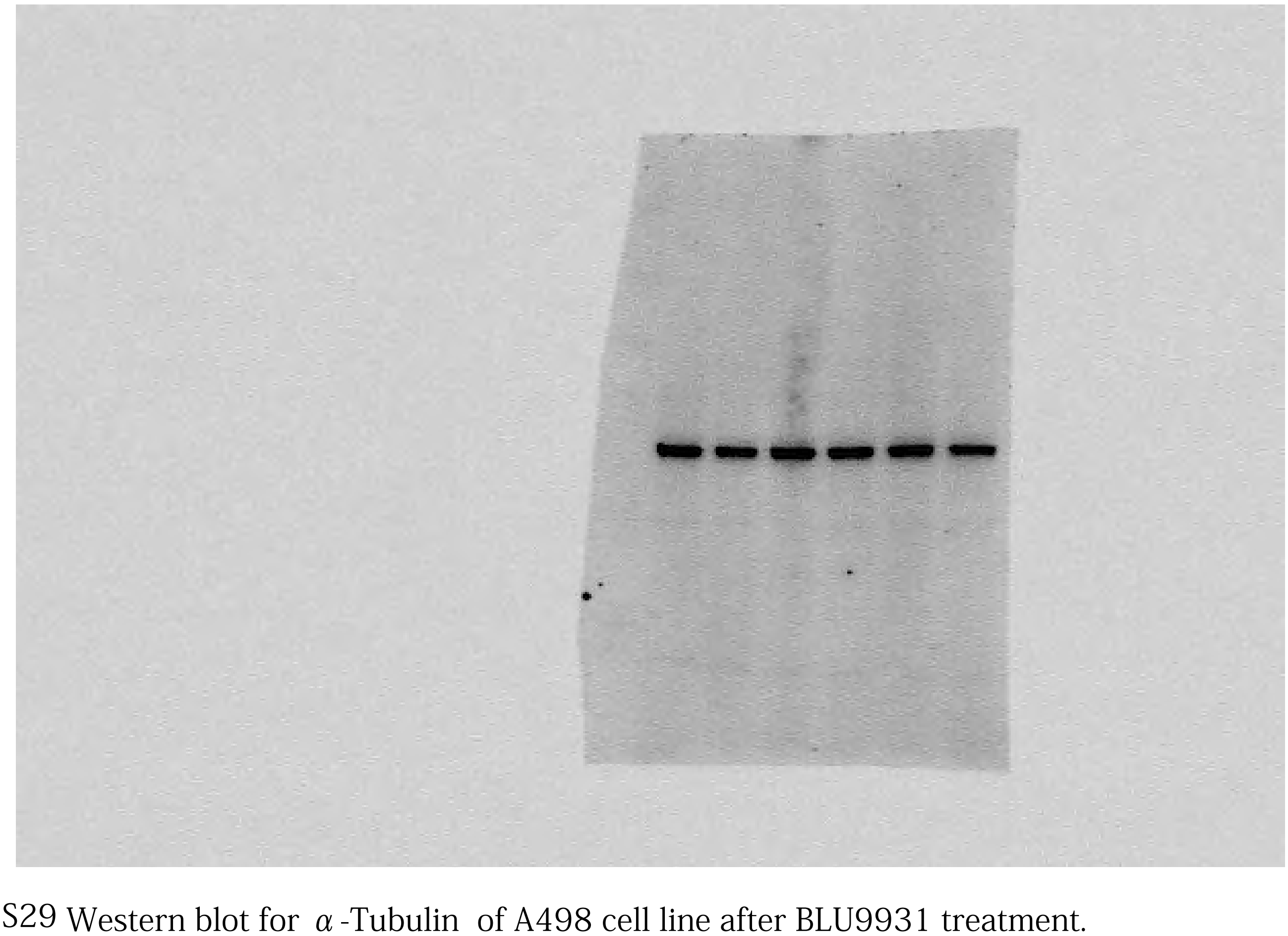

Figure S29 Western blot for α-Tubulin of A498 cell line after BLU9931 treatment. From left, Control, 1 nM, 10 nM, 100 nM, 1 μM, 10 μM with BLU9931 treatment concentrations.

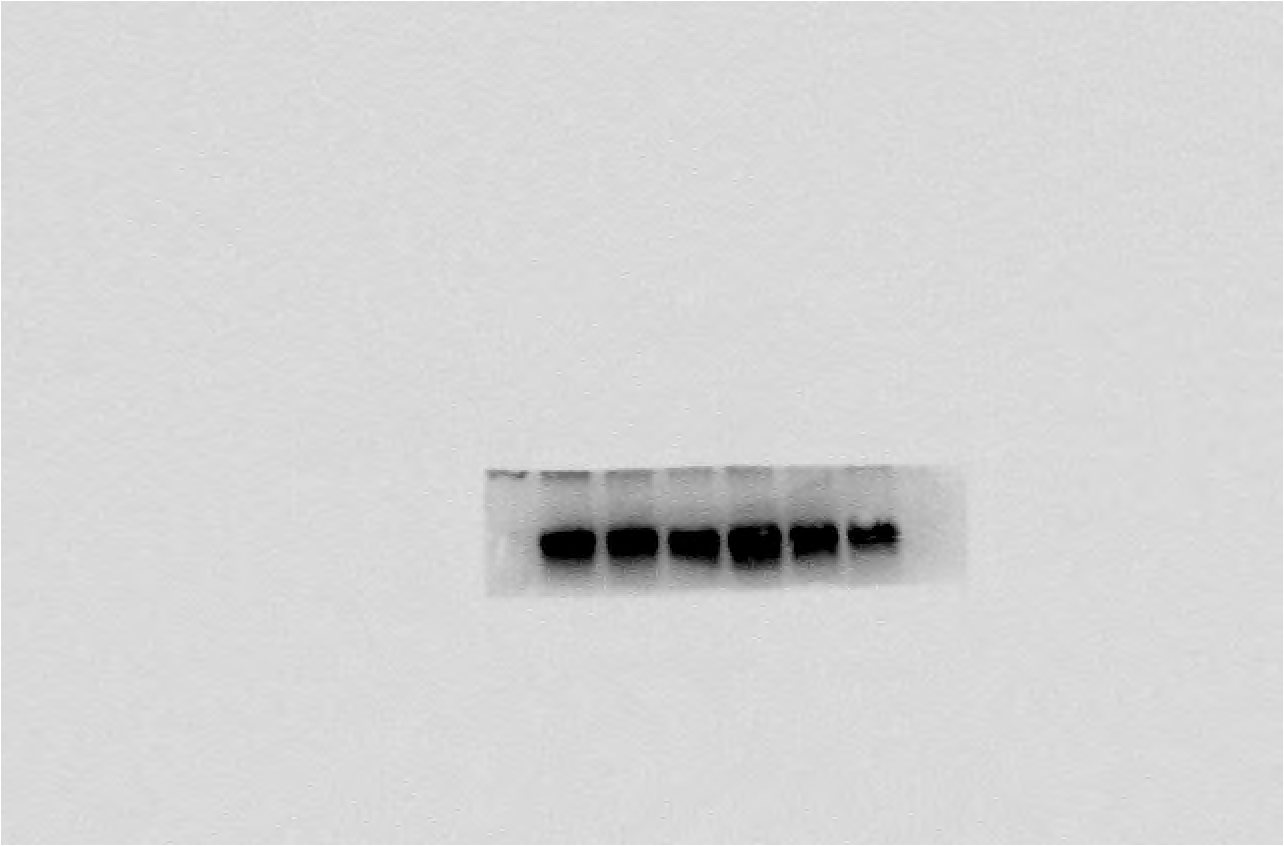

Figure S30 Western blot for AKT of A498 cell line after BLU9931 treatment.
From left, Control, 1 nM, 10 nM, 100 nM, 1 μM, 10 μM with BLU9931 treatment concentrations.

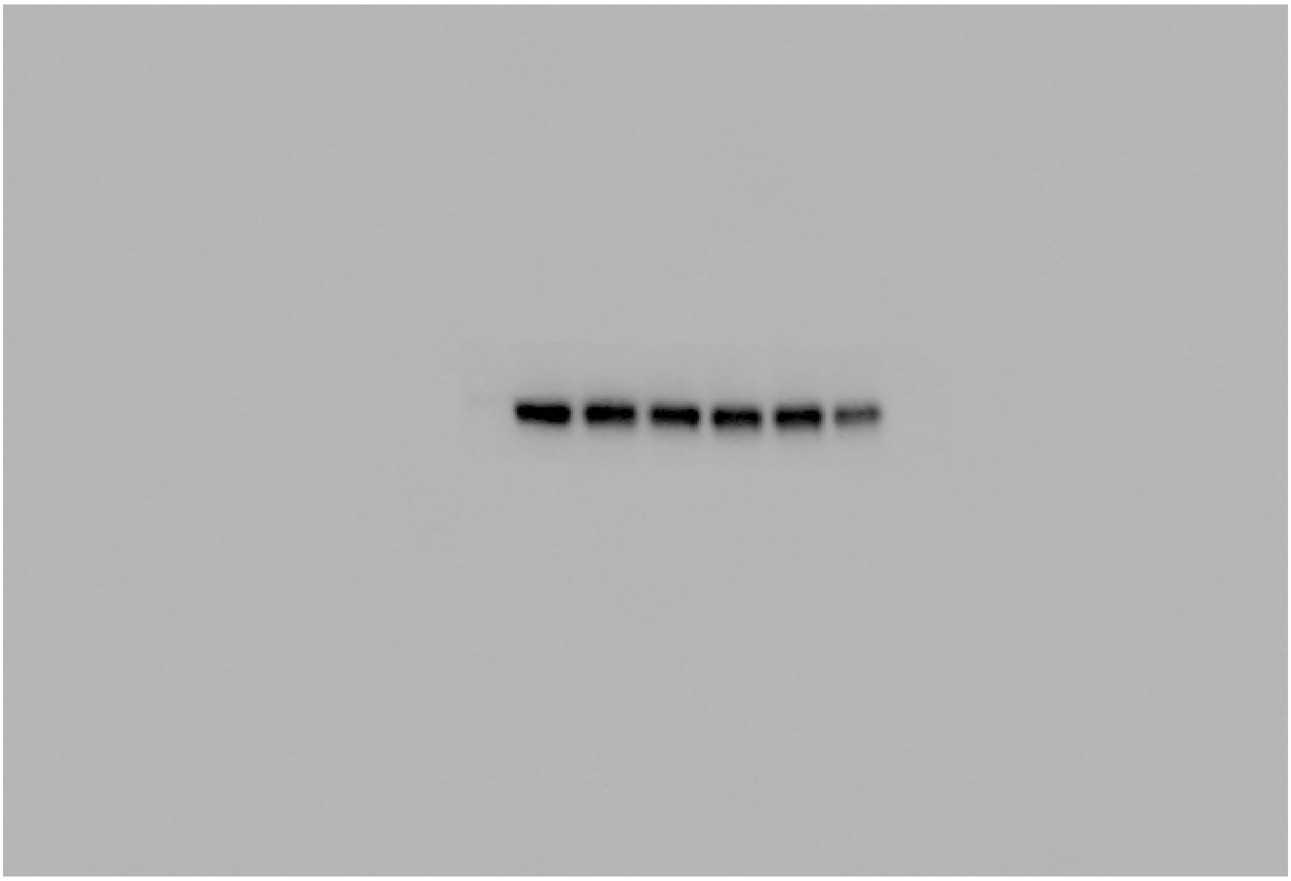

Figure S31 Western blot for phospho-AKT (Ser473) of A498 cell line after BLU9931 treatment.
From left, Control, 1 nM, 10 nM, 100 nM, 1 μM, 10 μM with BLU9931 treatment concentrations.

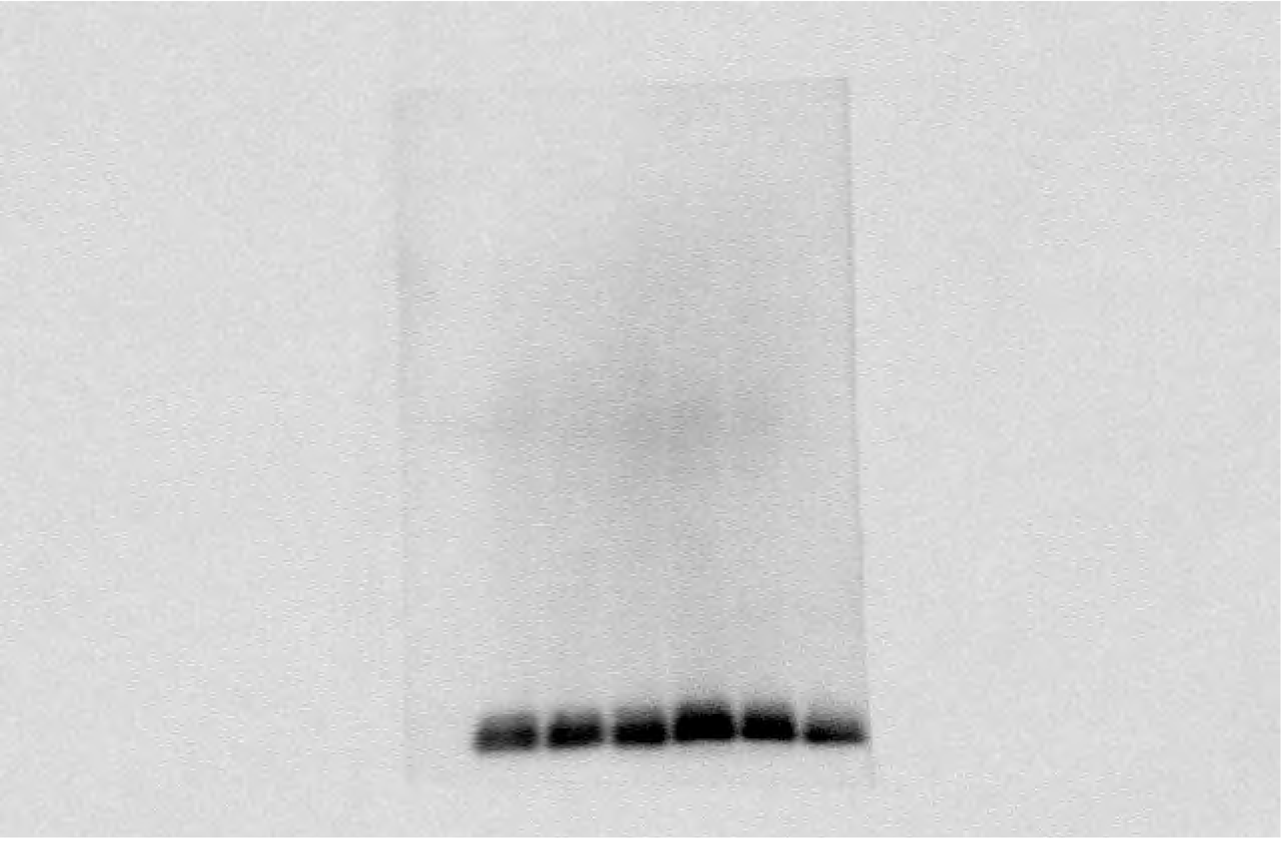

Figure S32 Western blot for 4EBP1 of A498 cell line after BLU9931 treatment.
From left, Control, 1 nM, 10 nM, 100 nM, 1 μM, 10 μM with BLU9931 treatment concentrations.

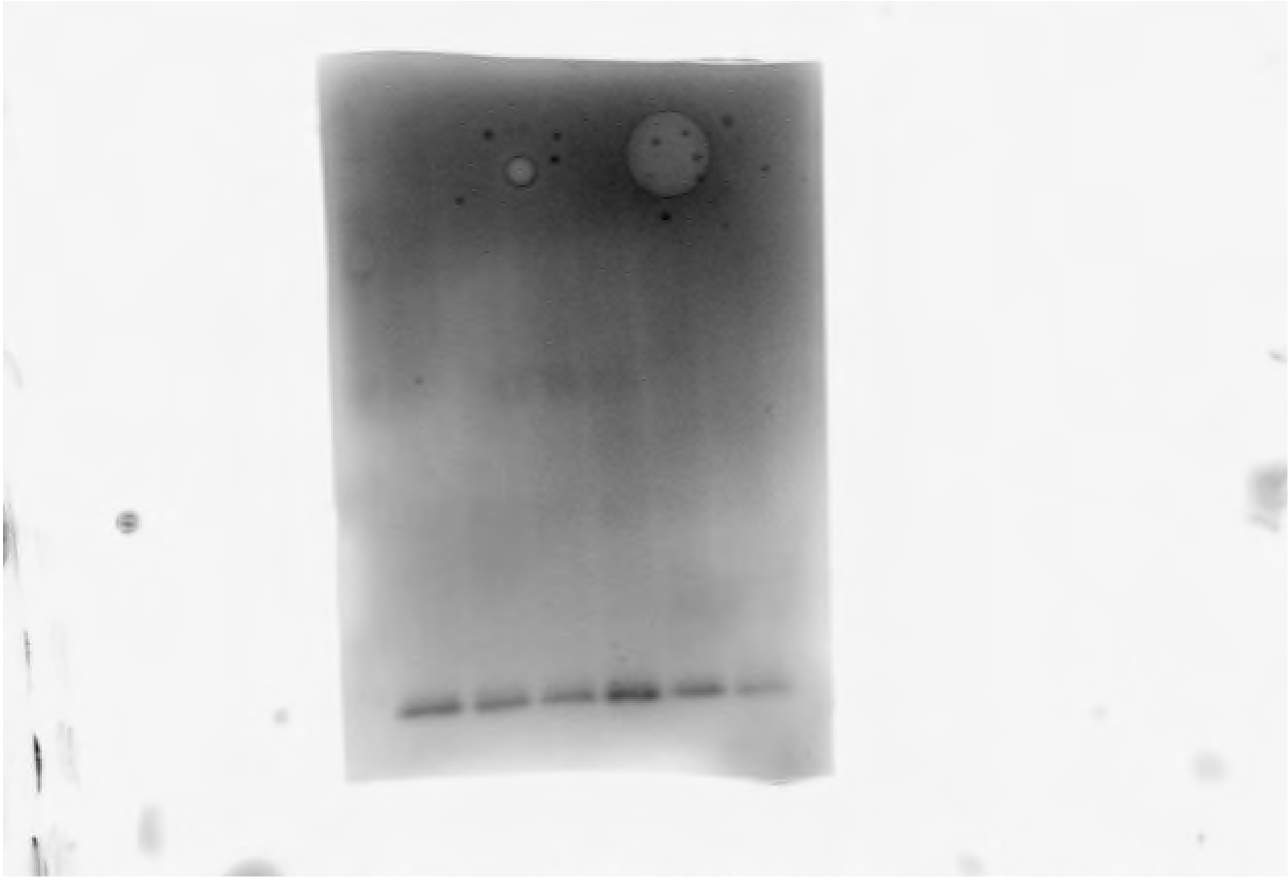

Figure S33 Western blot for phospho-4EBP1 (Thr70) of A498 cell line after BLU9931 treatment. From left, Control, 1 nM, 10 nM, 100 nM, 1 μM, 10 μM with BLU9931 treatment concentrations.

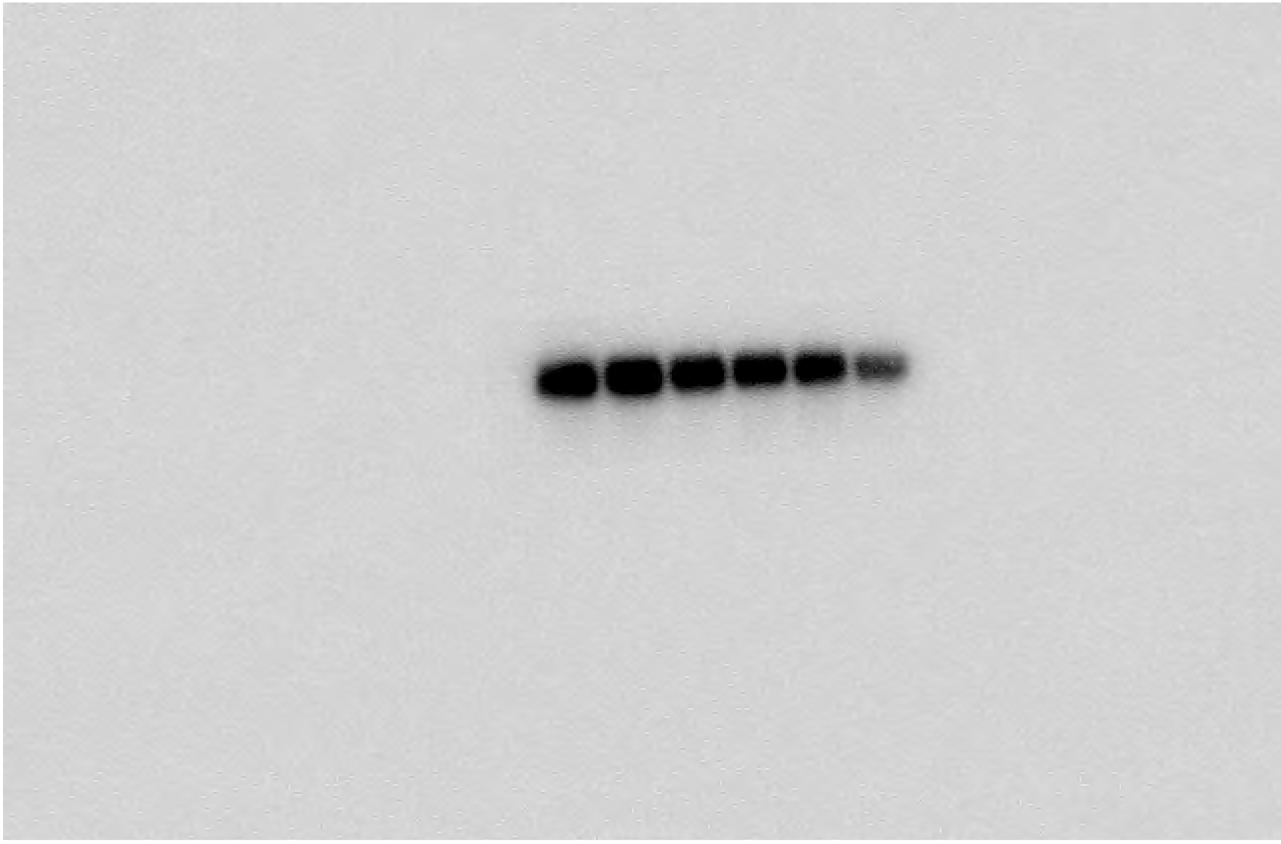

Figure S34 Western blot for S6RP of A498 cell line after BLU9931 treatment.
From left, Control, 1 nM, 10 nM, 100 nM, 1 μM, 10 μM
with BLU9931 treatment concentrations.

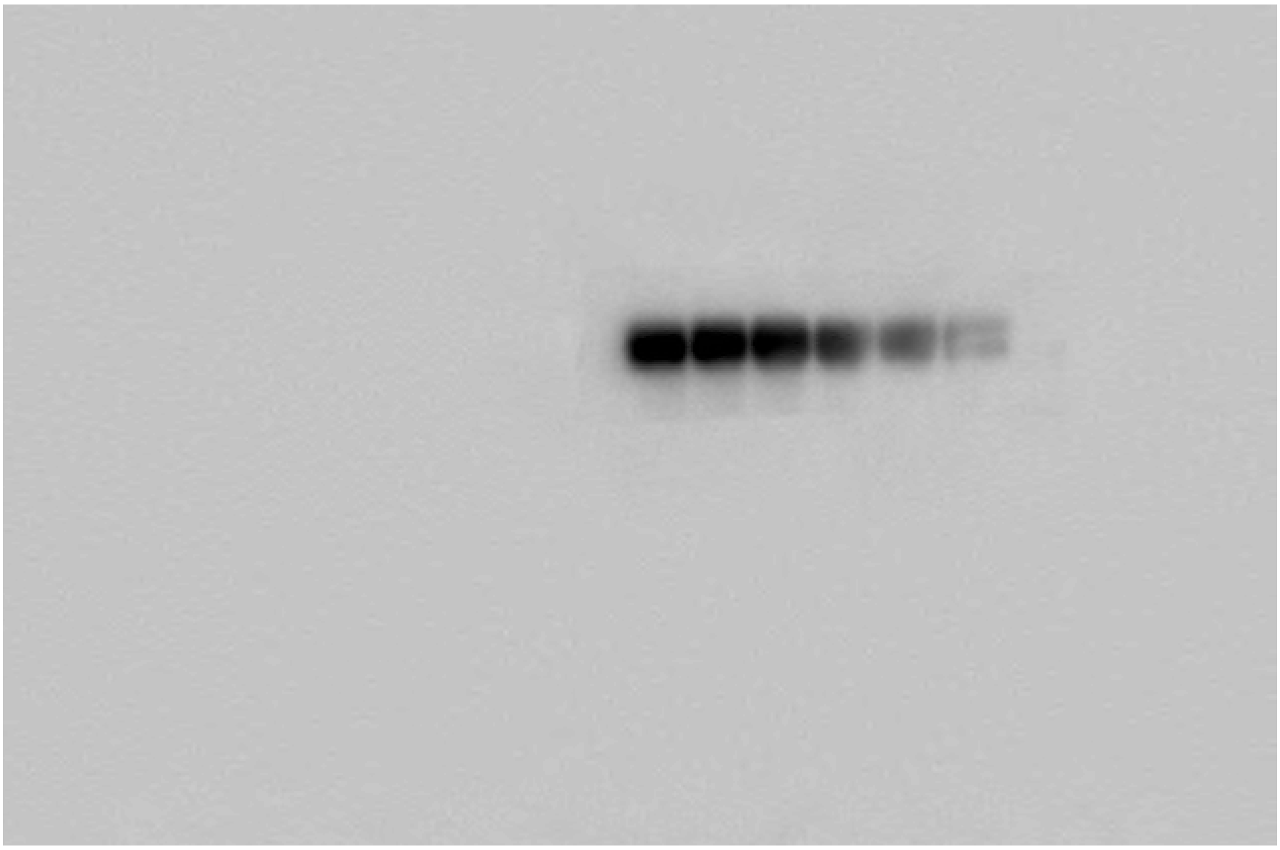

Figure S35 Western blot for phospho-S6RP (Ser235/236) of A498 cell line after BLU9931 treatment.
From left, Control, 1 nM, 10 nM, 100 nM, 1 μM, 10 μM
with BLU9931 treatment concentrations.

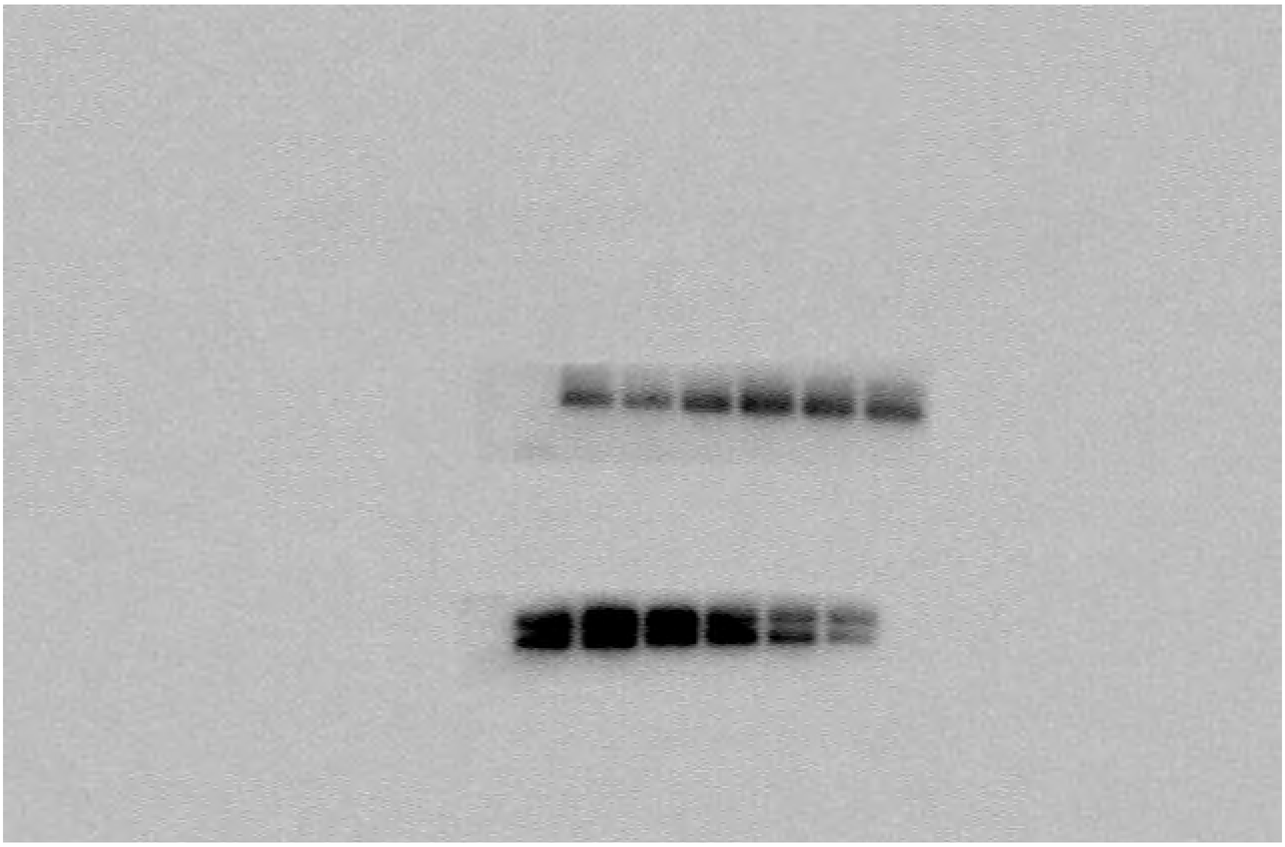

Figure S36 Western blot for ERK1/2 of A498 cell line after BLU9931 treatment (above).
From left, Control, 1 nM, 10 nM, 100 nM, 1 μM, 10 μM with BLU9931 treatment concentrations. (The below membrane showed a reproductive result of phospho-ERK1/2.)

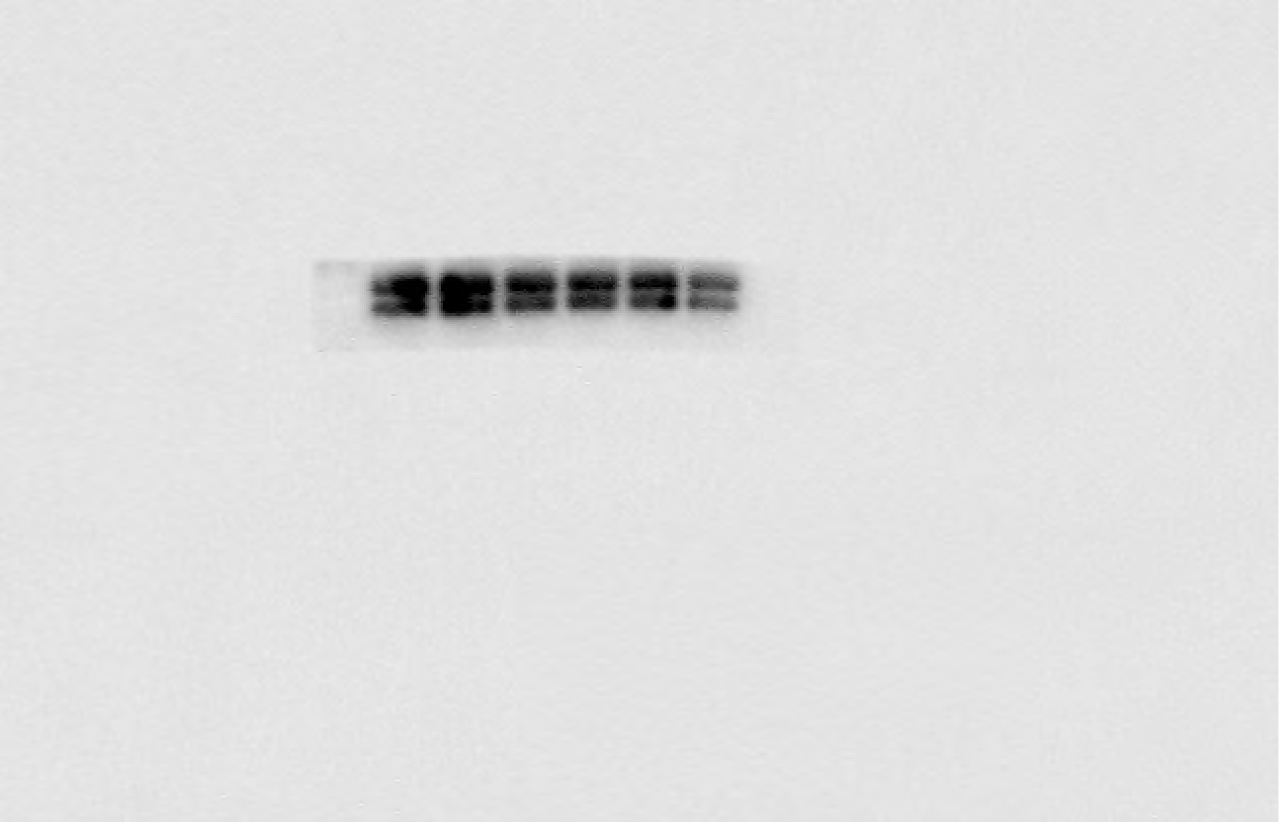

Figure S37 Western blot for phospho-ERK1/2 (Thr202/Tyr204) of A498 cell line after BLU9931 treatment. From left, Control, 1 nM, 10 nM, 100 nM, 1 μM, 10 μM with BLU9931 treatment concentrations.

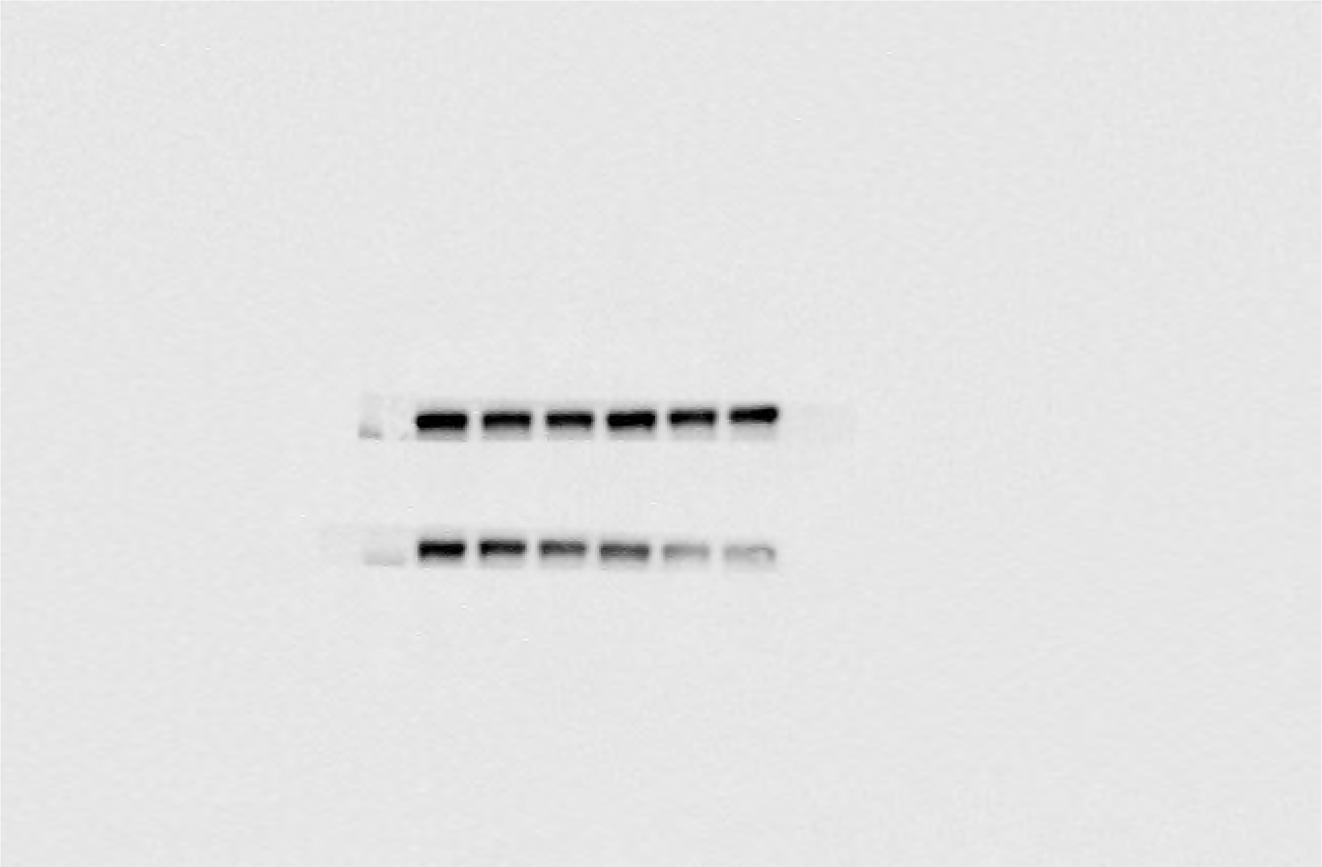

Figure S38 Western blot for STAT3 (above) and phospho-STAT3 (Tyr705, below) of A498 cell line after BLU9931 treatment. From left, Control, 1 nM, 10 nM, 100 nM, 1 μM, 10 μM with BLU9931 treatment concentrations.

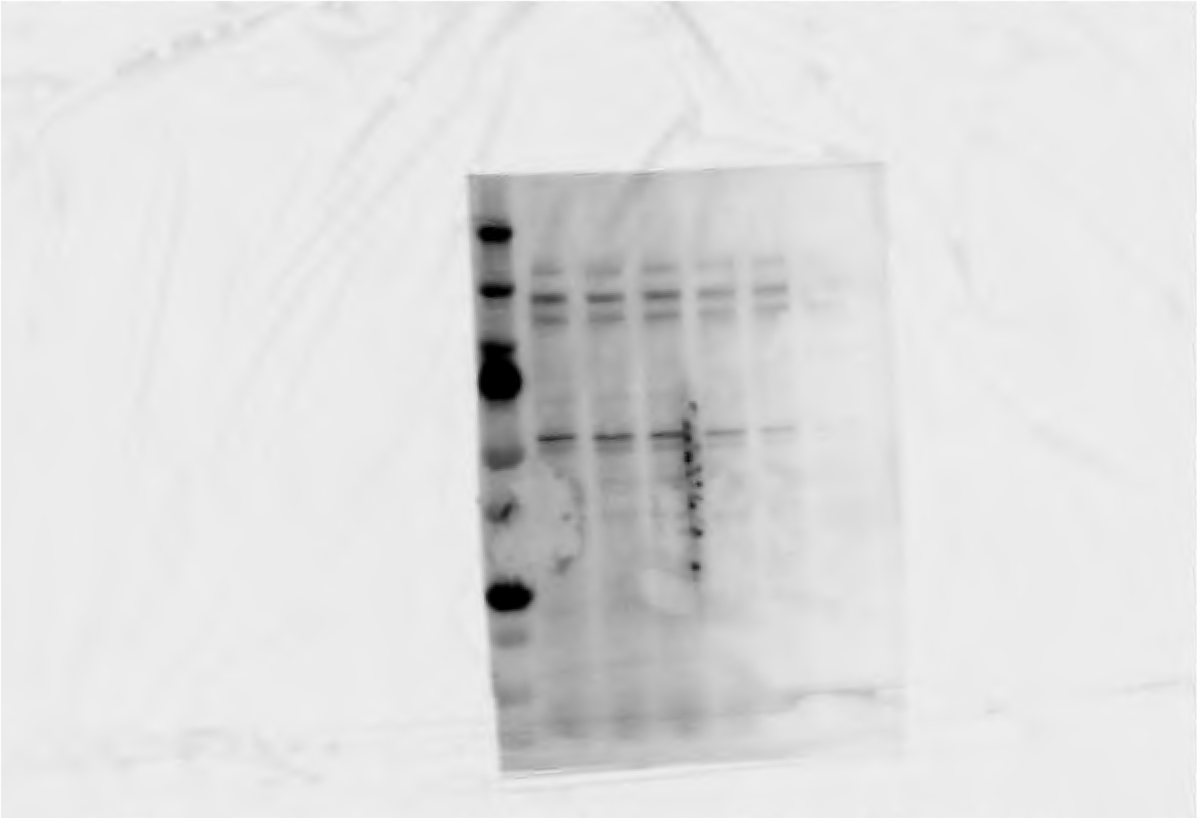

Figure S39 Western blot for phospho-FGFR (Thr653/654) of A704 cell line after BLU9931 treatment. From left, MW marker, From left, Control, 1 nM, 10 nM, 100 nM, 1 μM, 10 μM with BLU9931 treatment concentrations.

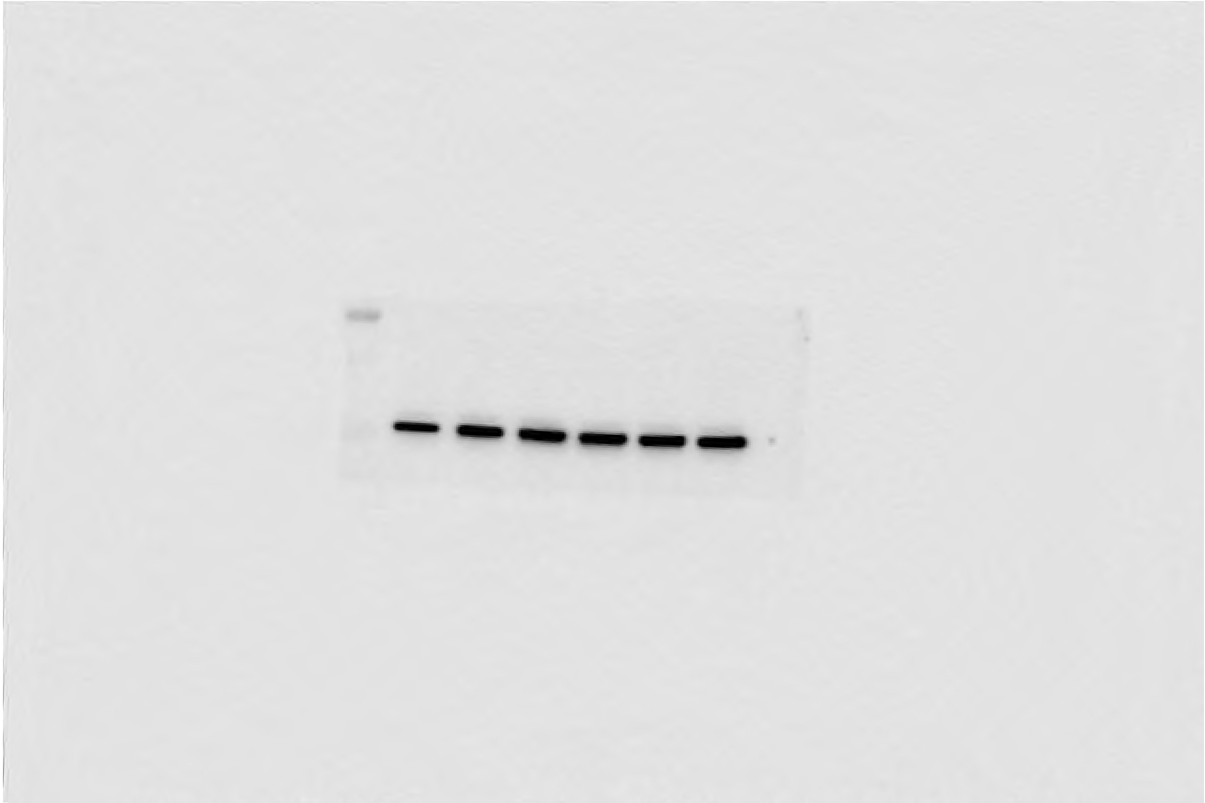

Figure S40 Western blot for α-Tubulin of A704 cell line after BLU9931 treatment. From left, Control, 1 nM, 10 nM, 100 nM, 1 μM, 10 μM with BLU9931 treatment concentrations.

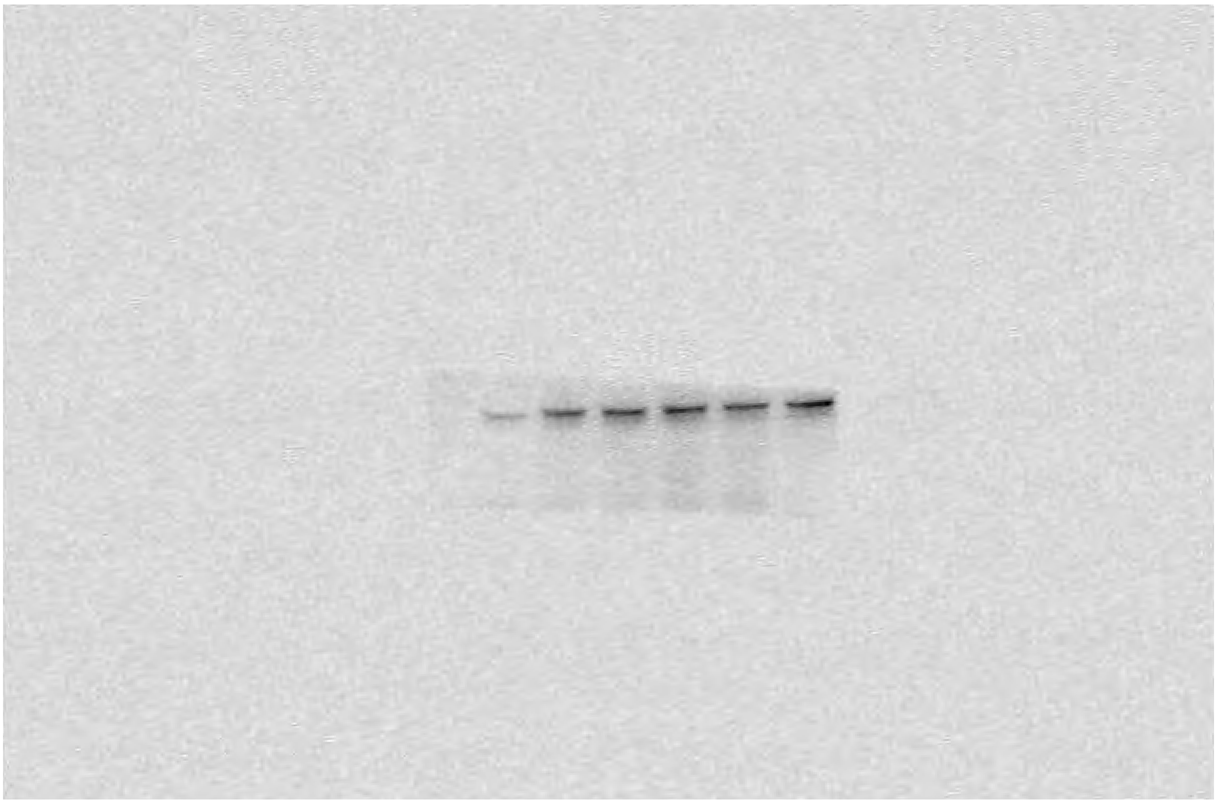

Figure S41 Western blot for AKT of A704 cell line after BLU9931 treatment.
From left, Control, 1 nM, 10 nM, 100 nM, 1 μM, 10 μM with BLU9931 treatment concentrations.

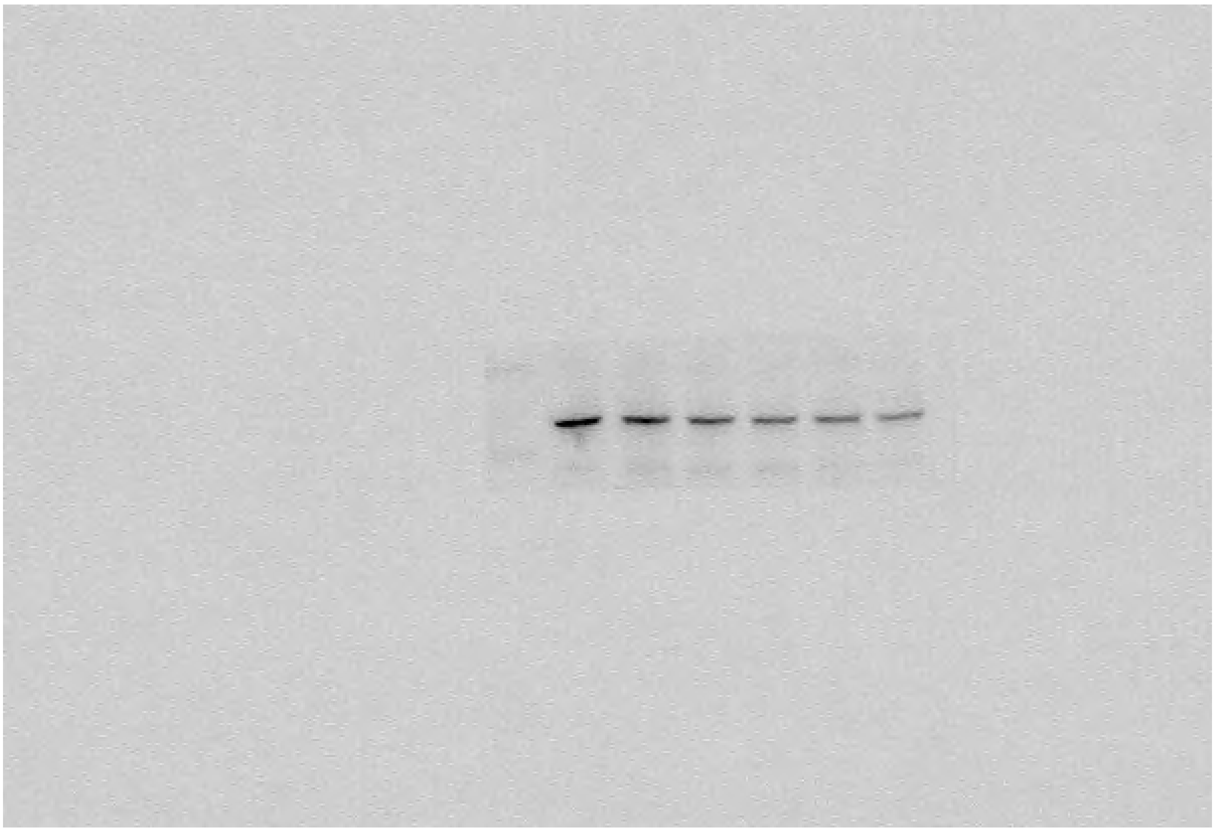

Figure S42 Western blot for phospho-AKT (Ser473) of A704 cell line after BLU9931 treatment.
From left, Control, 1 nM, 10 nM, 100 nM, 1 μM, 10 μM with BLU9931 treatment concentrations.

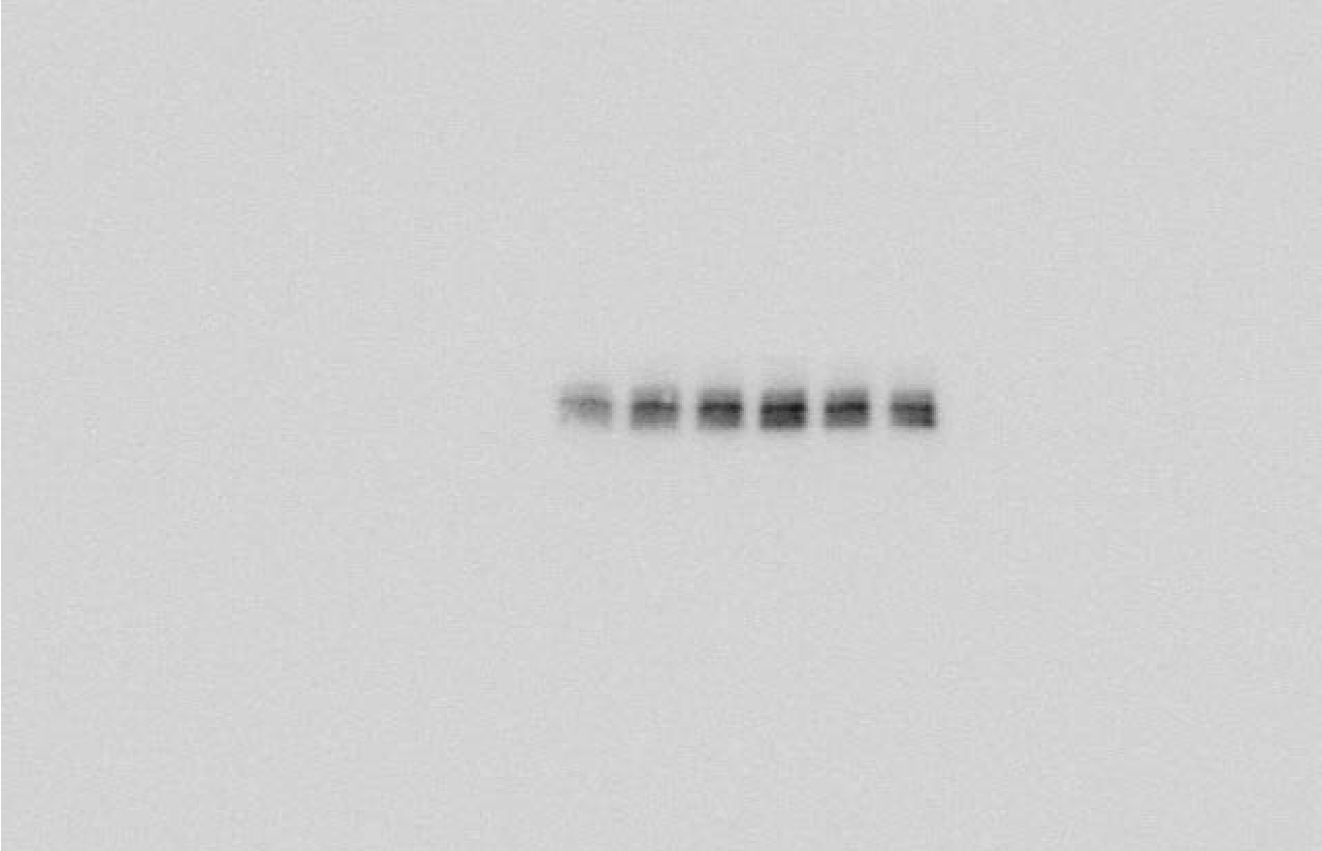

Figure S43 Western blot for 4EBP1 of A704 cell line after BLU9931 treatment.
From left, Control, 1 nM, 10 nM, 100 nM, 1 μM, 10 μM with BLU9931 treatment concentrations.

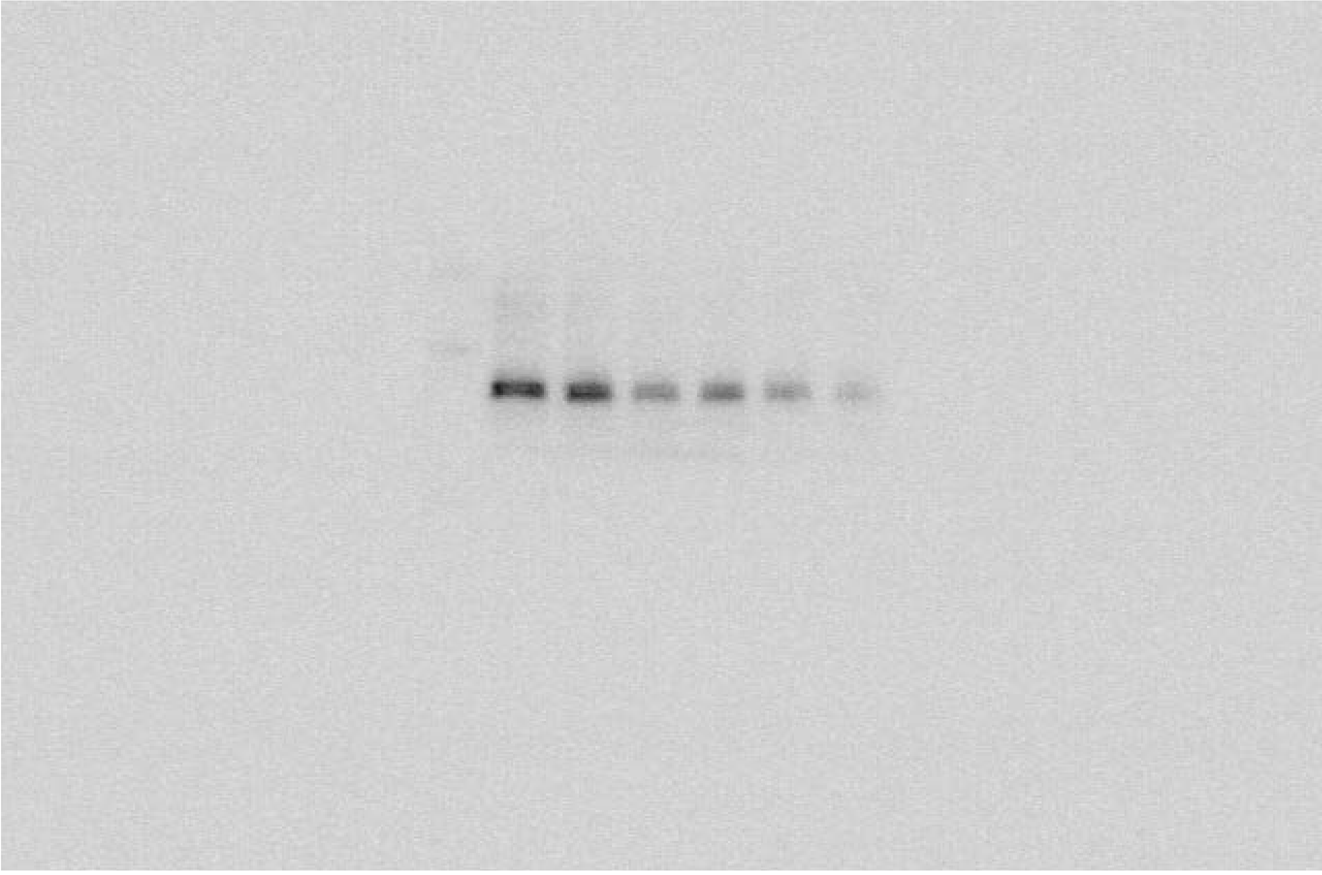

Figure S44 Western blot for phospho-4EBP1 (Thr70) of A704 cell line after BLU9931 treatment.
From left, Control, 1 nM, 10 nM, 100 nM, 1 μM, 10 μM with BLU9931 treatment concentrations.

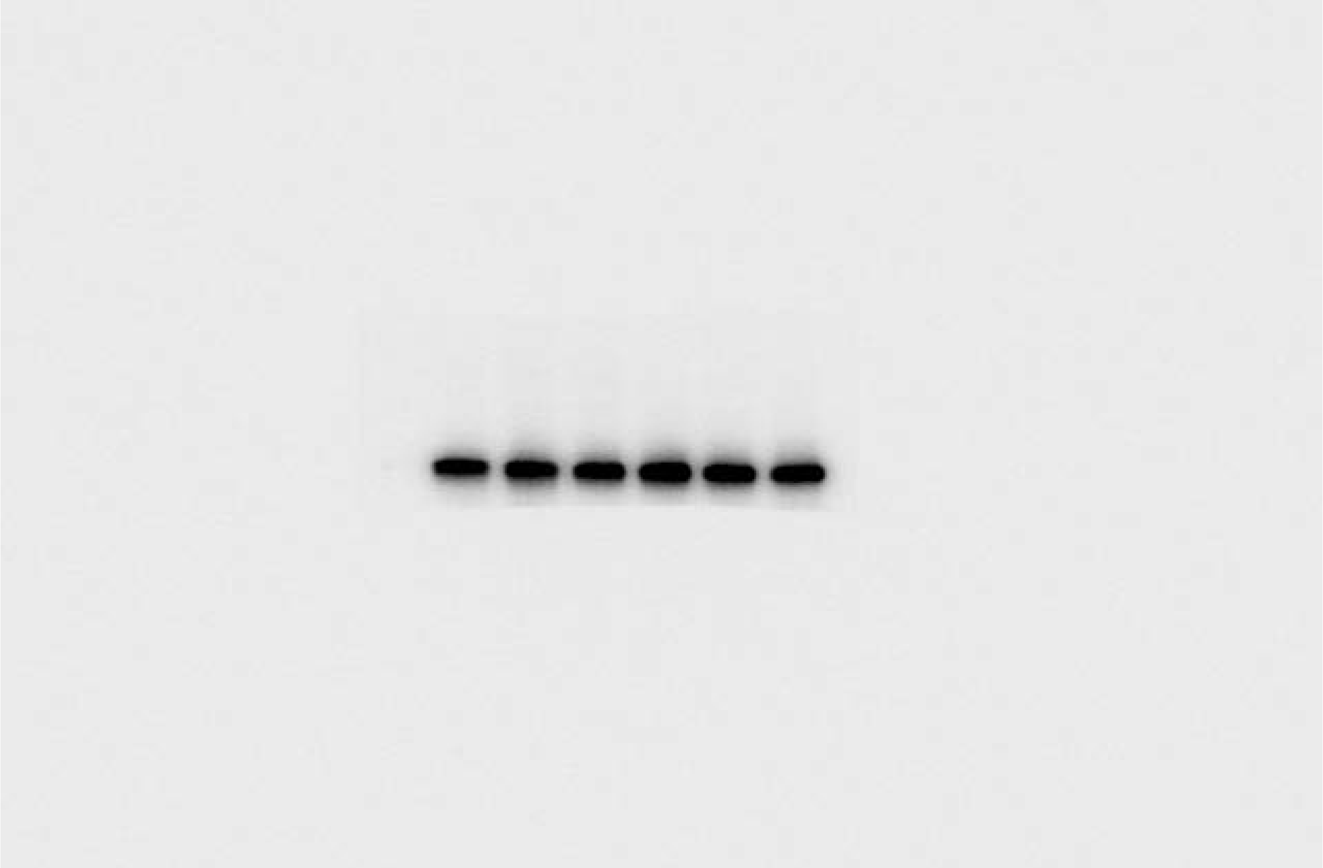

Figure S45 Western blot for S6RP of A704 cell line after BLU9931 treatment. From left, Control, 1 nM, 10 nM, 100 nM, 1 μM, 10 μM with BLU9931 treatment concentrations.

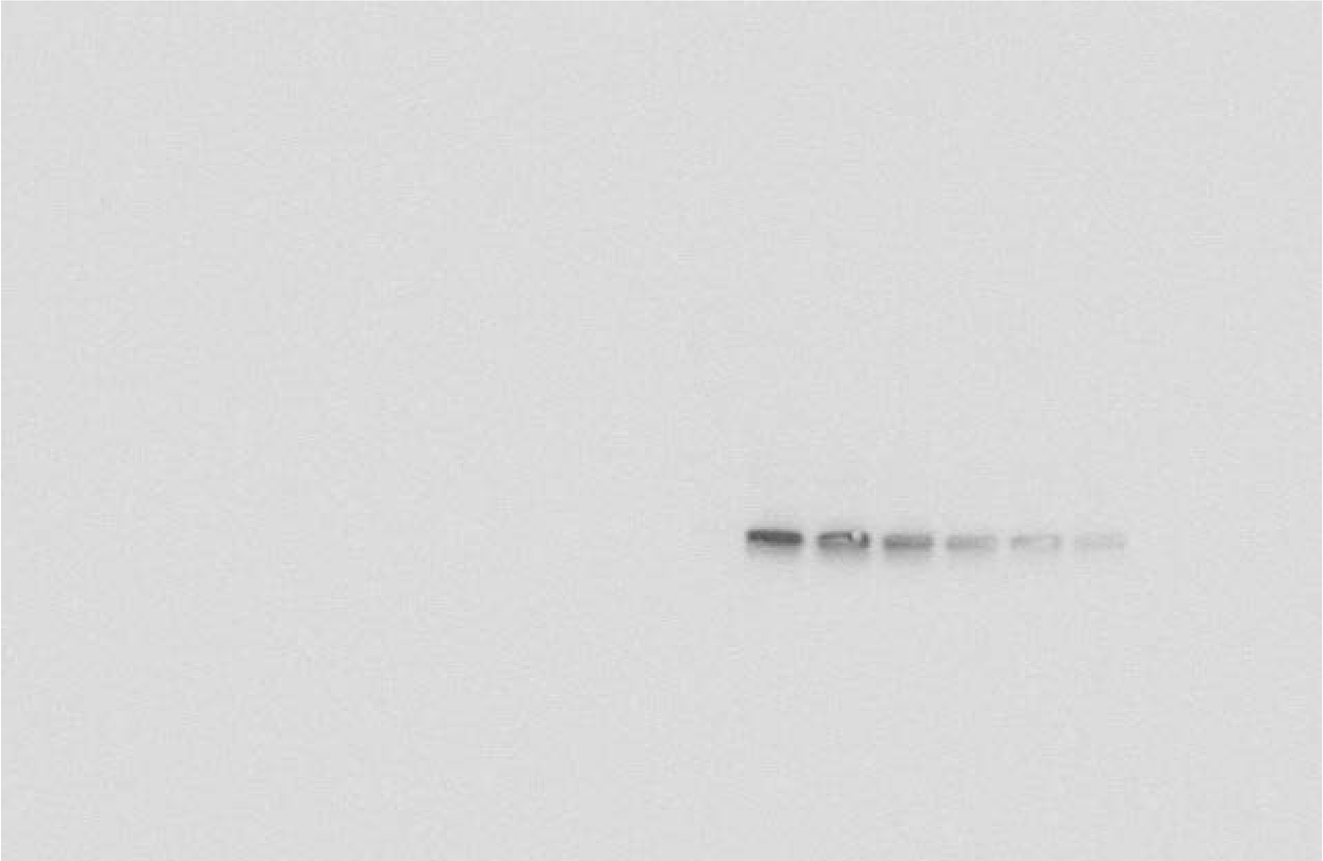

Figure S46 Western blot for phospho-S6RP (Ser235/236) of A704 cell line after BLU9931 treatment. From left, Control, 1 nM, 10 nM, 100 nM, 1 μM, 10 μM with BLU9931 treatment concentrations.

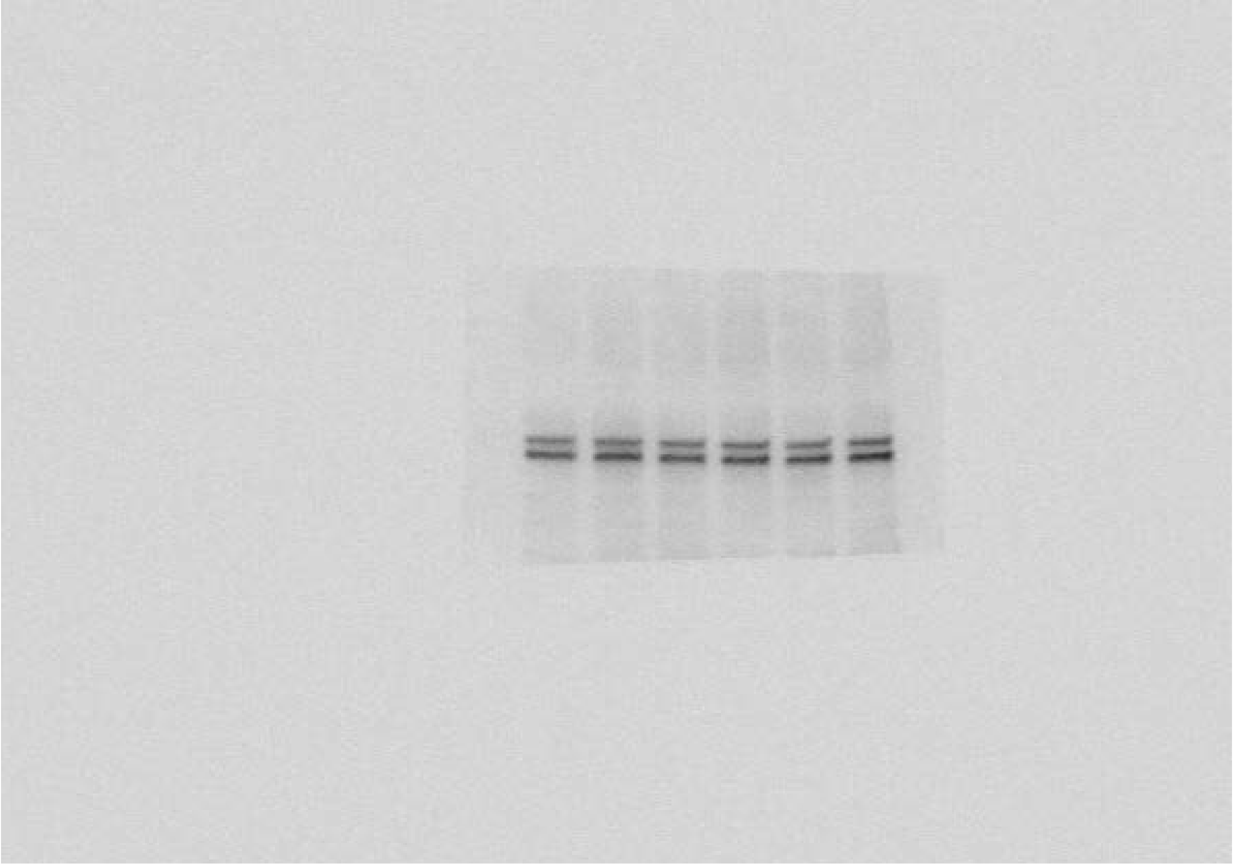

Figure S47 Western blot for ERK1/2 of A704 cell line after BLU9931 treatment. From left, Control, 1 nM, 10 nM, 100 nM, 1 μM, 10 μM with BLU9931 treatment concentrations.

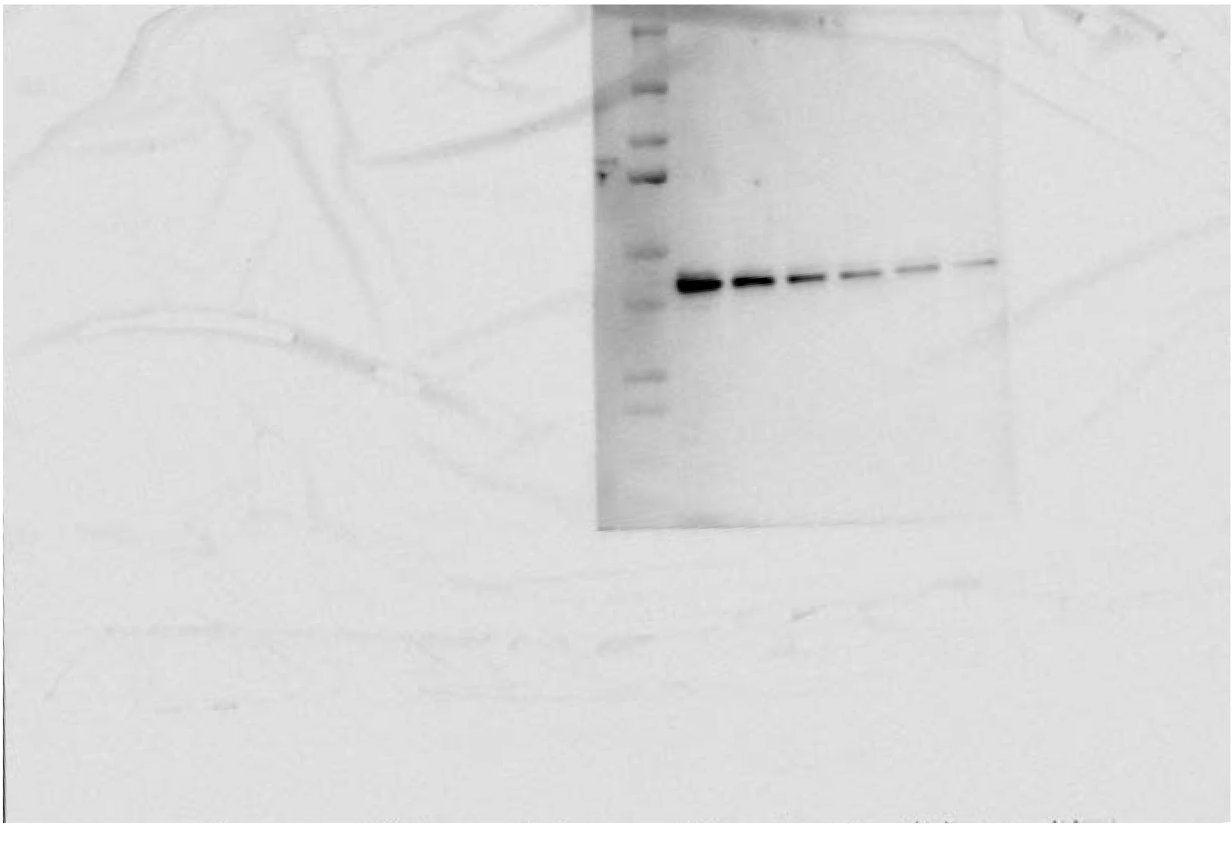

Figure S48 Western blot for phospho-ERK1/2 (Thr202/Tyr204) of A704 cell line after BLU9931 treatment. From left, Control, 1 nM, 10 nM, 100 nM, 1 μM, 10 μM with BLU9931 treatment concentrations.

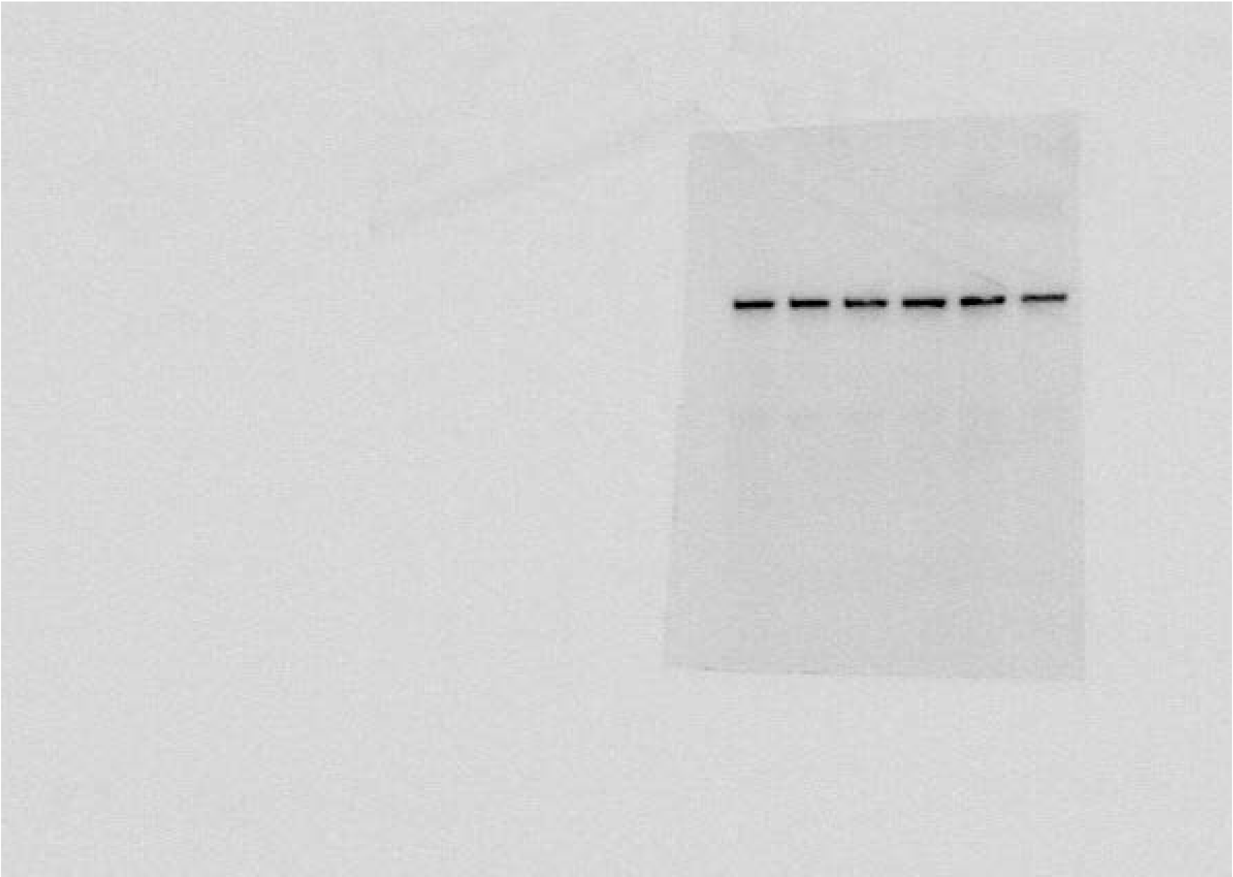

Figure S49 Western blot for STAT3 of A704 cell line after BLU9931 treatment.
From left, Control, 1 nM, 10 nM, 100 nM, 1 μM, 10 μM with BLU9931 treatment concentrations.

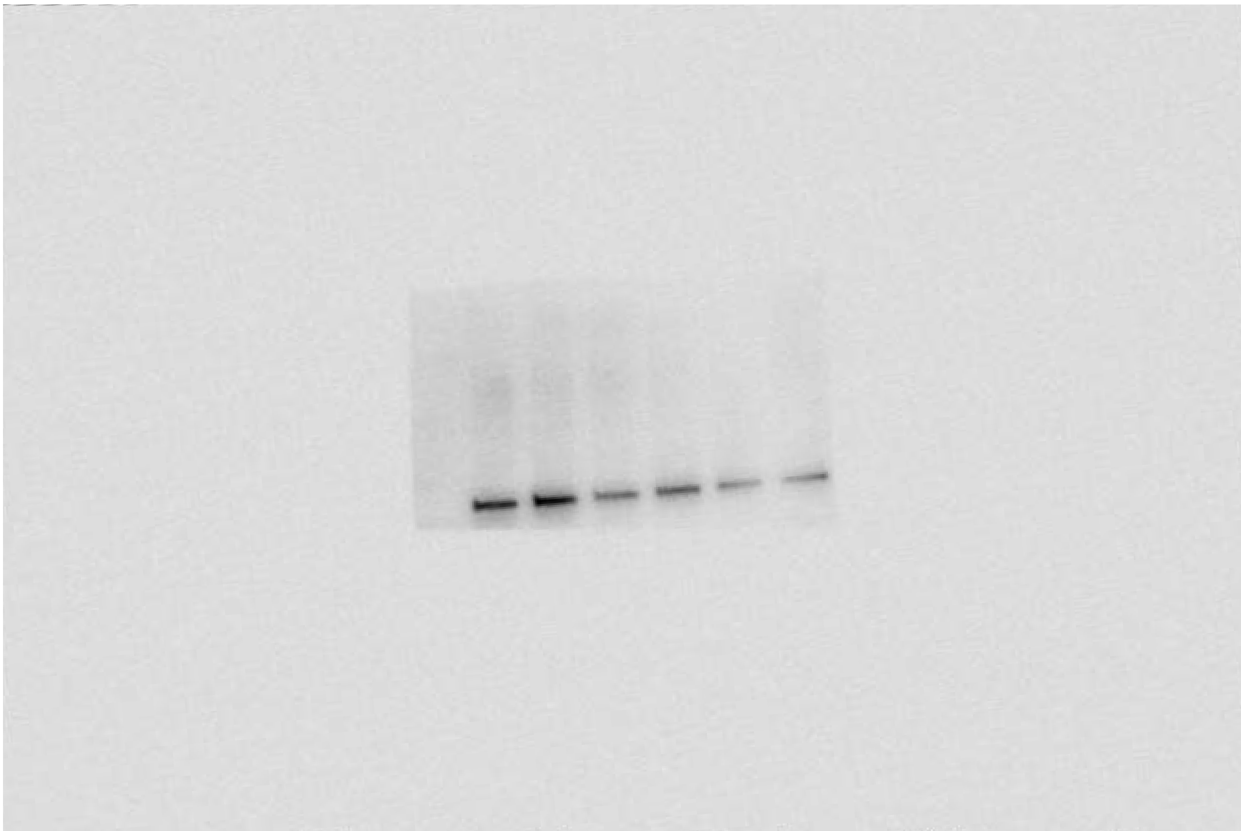

Figure S50 Western blot for phospho-STAT3 (Tyr705) of A704 cell line after BLU9931 treatment.
From left, Control, 1 nM, 10 nM, 100 nM, 1 μM, 10 μM with BLU9931 treatment concentrations.

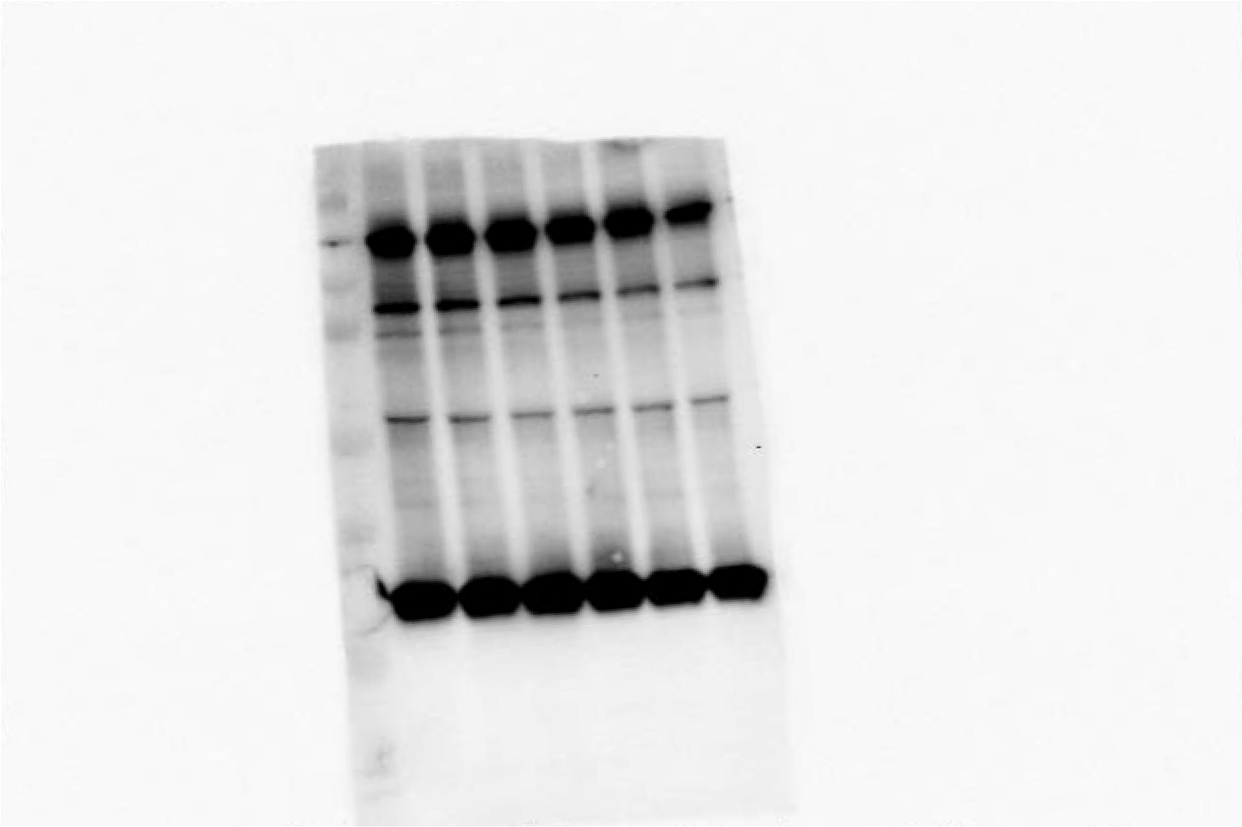

Figure S51 Western blot for phospho-FGFR (Thr653/654) of 769P cell line after BLU9931 treatment. From left, Control, 1 nM, 10 nM, 100 nM, 1 μM, 10 μM with BLU9931 treatment concentrations.

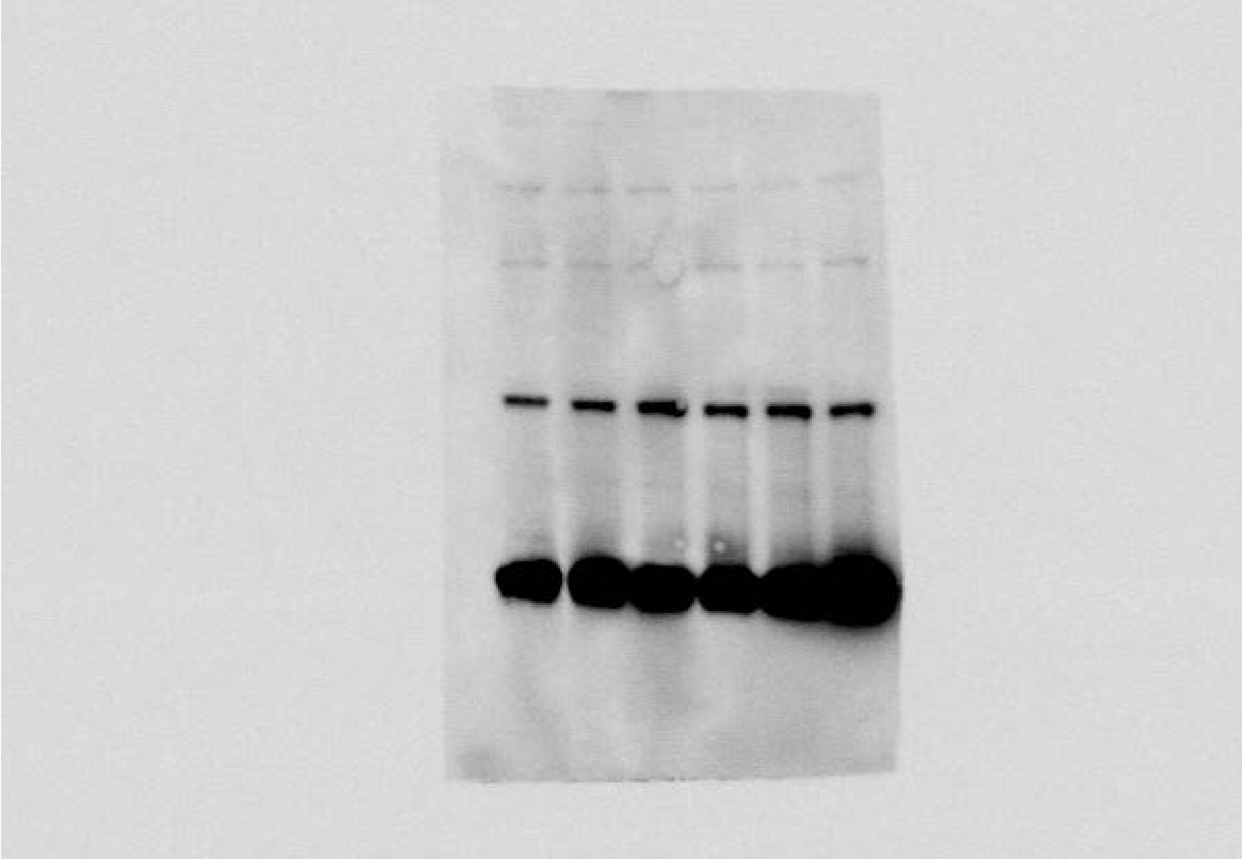

Figure S52 Western blot for α-Tubulin of 769P cell line after BLU9931 treatment. From left, Control, 1 nM, 10 nM, 100 nM, 1 μM, 10 μM with BLU9931 treatment concentrations.

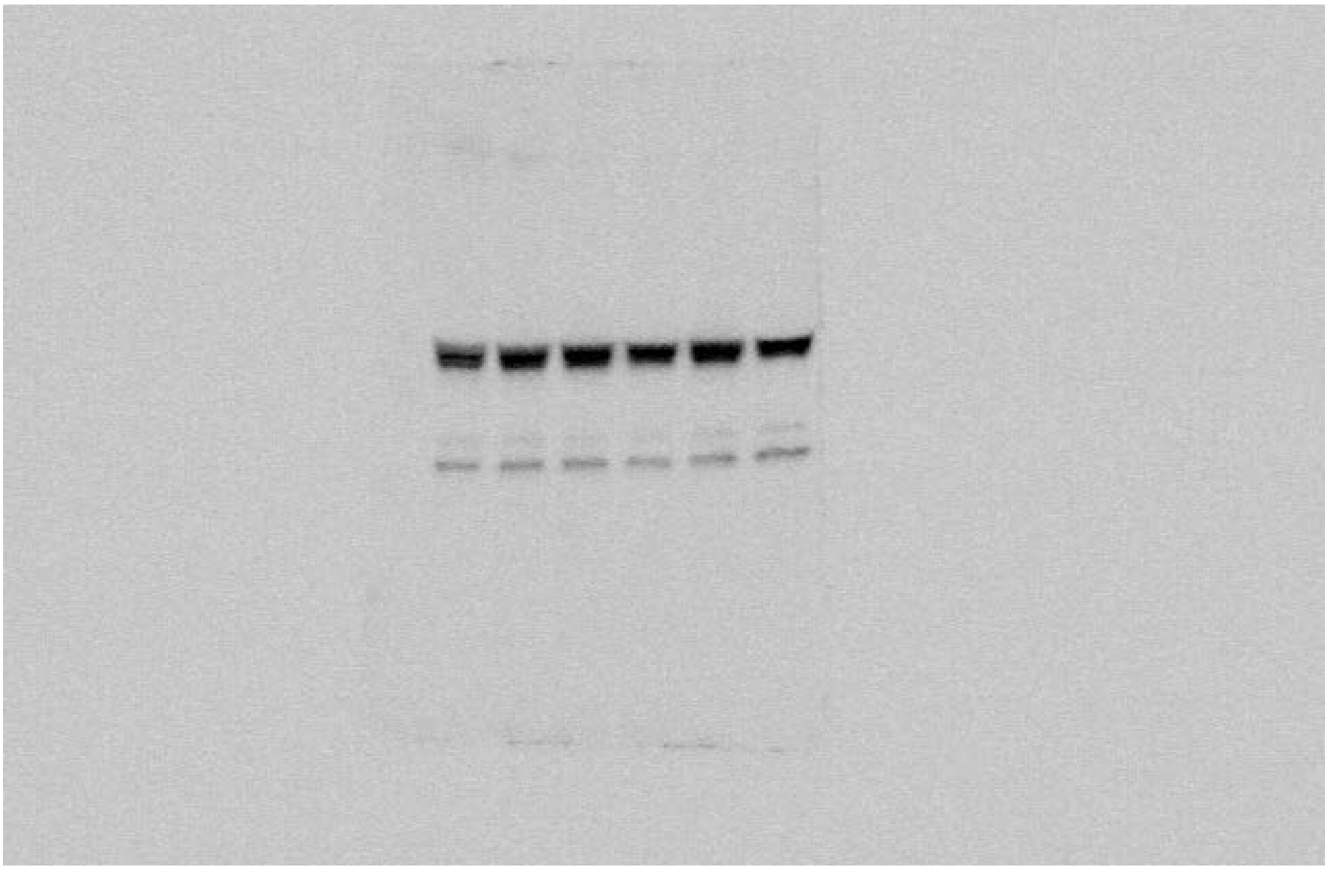

Figure S53 Western blot for AKT of 769P cell line after BLU9931 treatment.
From left, Control, 1 nM, 10 nM, 100 nM, 1 μM, 10 μM with BLU9931 treatment concentrations.

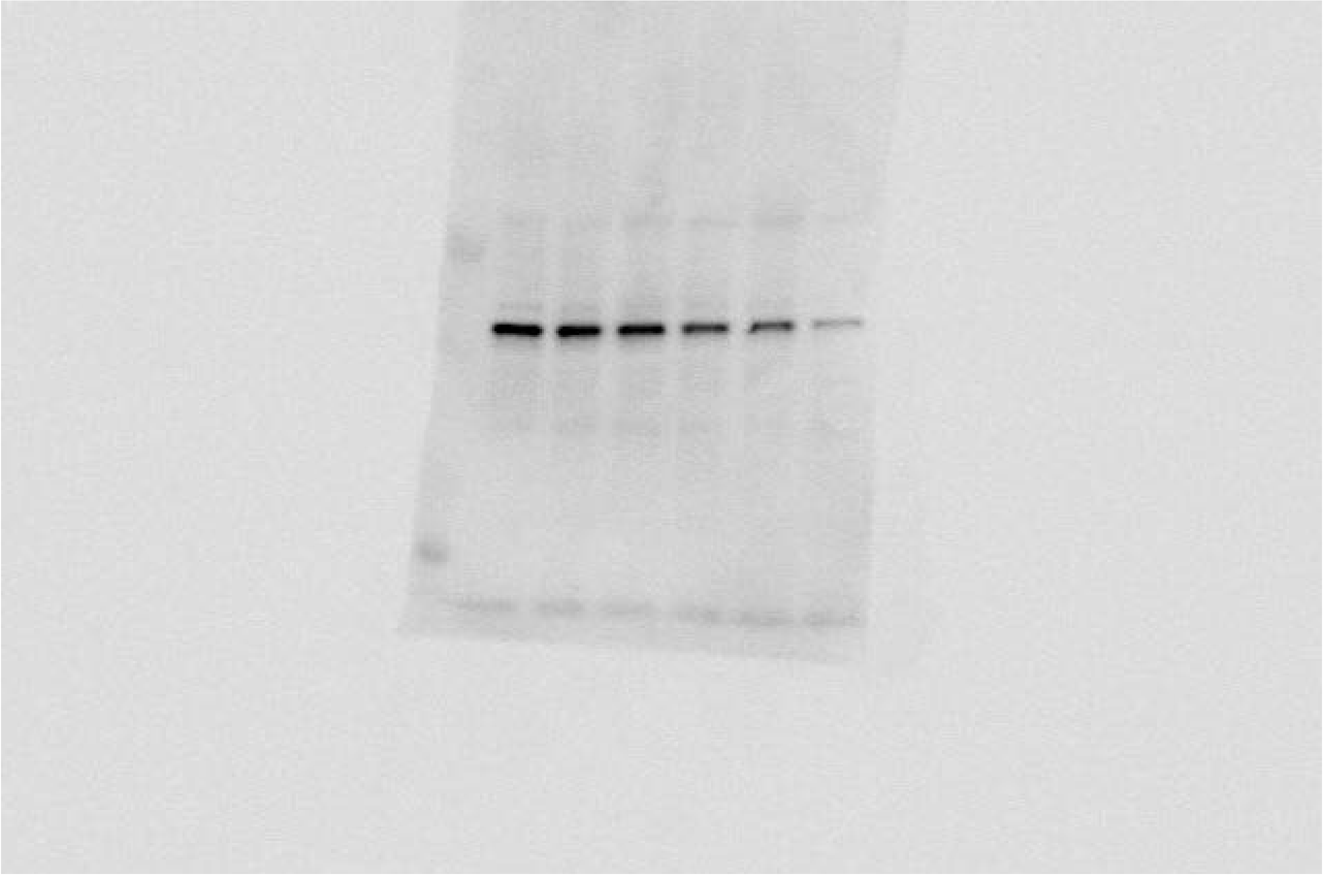

Figure S54 Phospho-AKT (Ser 473) of 769P cell line after BLU9931 treatment.
From left, Control, 1 nM, 10 nM, 100 nM, 1 μM, 10 μM with BLU9931 treatment concentrations.

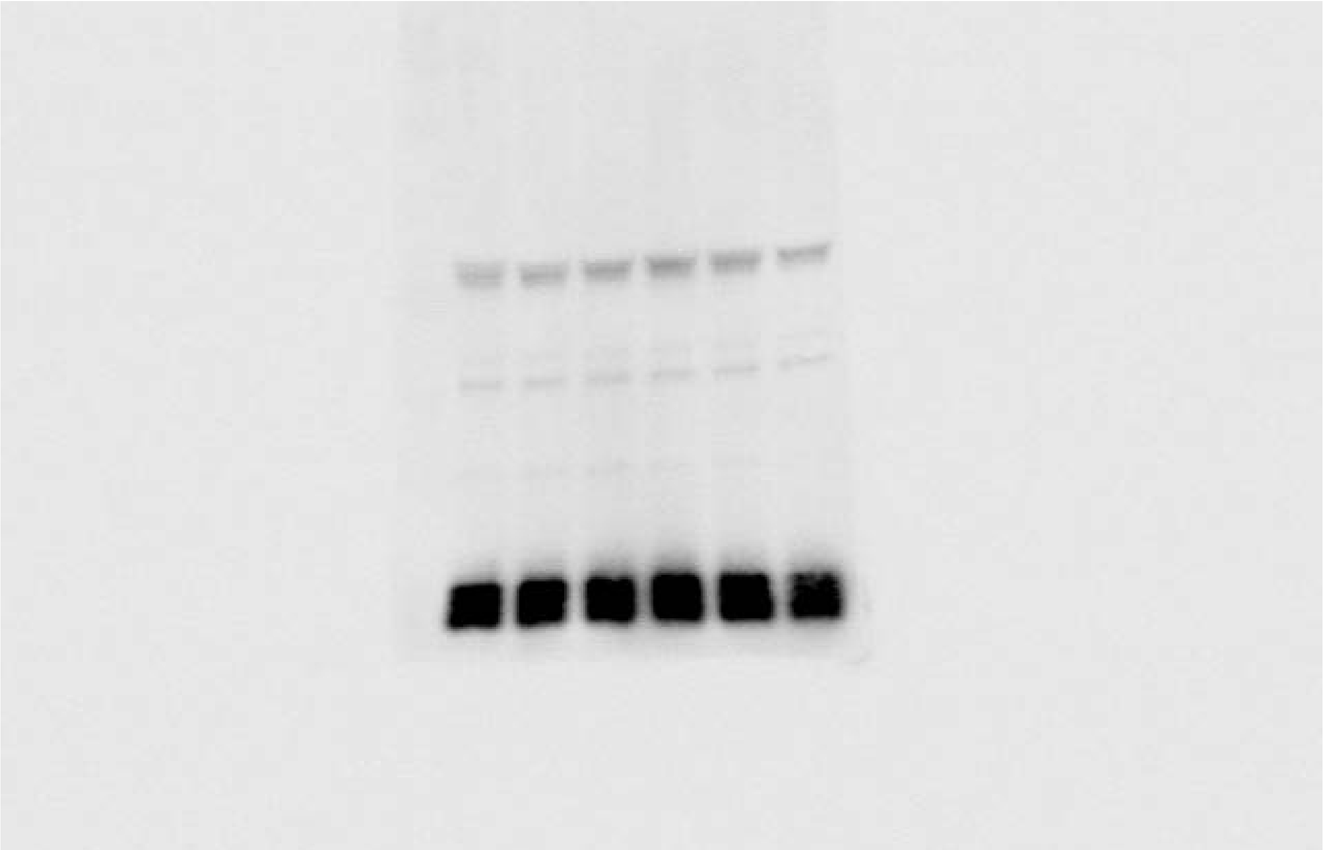

Figure S55 Western blot for 4EBP1 of 769P cell line after BLU9931 treatment.
From left, Control, 1 nM, 10 nM, 100 nM, 1 μM, 10 μM with BLU9931 treatment concentrations.

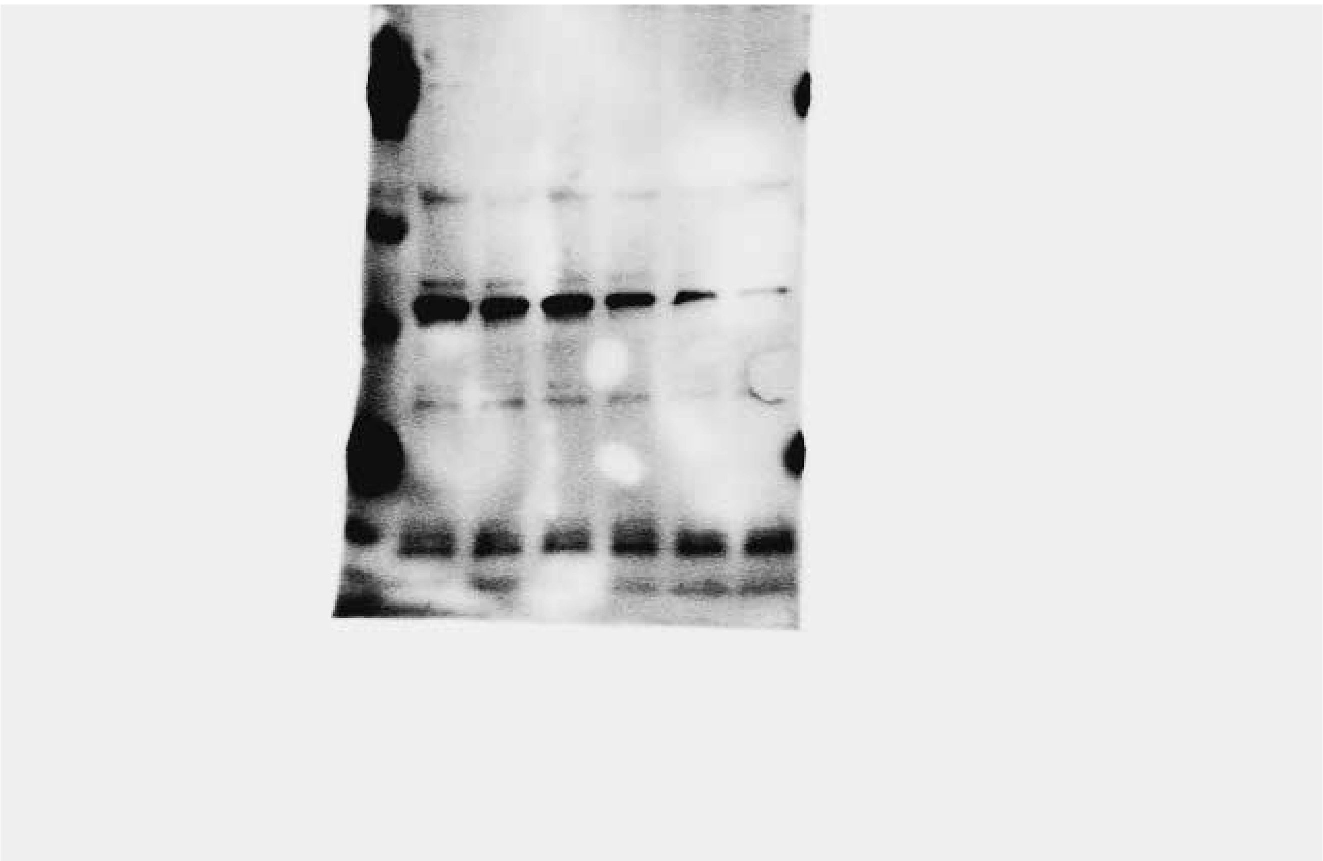

Figure S56 Western blot for phospho-4EBP1 (Thr70) of 769P cell line after BLU9931 treatment.
From left, Control, 1 nM, 10 nM, 100 nM, 1 μM, 10 μM with BLU9931 treatment concentrations.

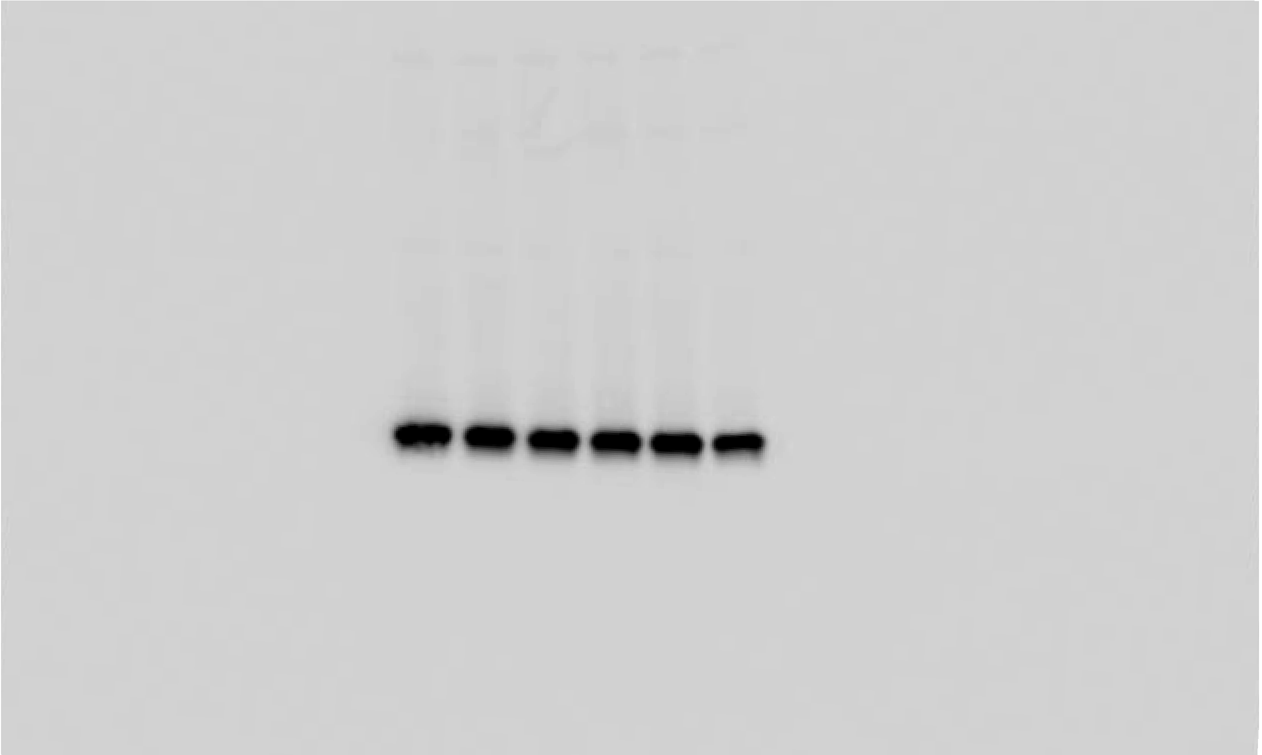

Figure S57 Western blot for S6RP of 769P cell line after BLU9931 treatment.
From left, Control, 1 nM, 10 nM, 100 nM, 1 μM, 10 μM with BLU9931 treatment concentrations.

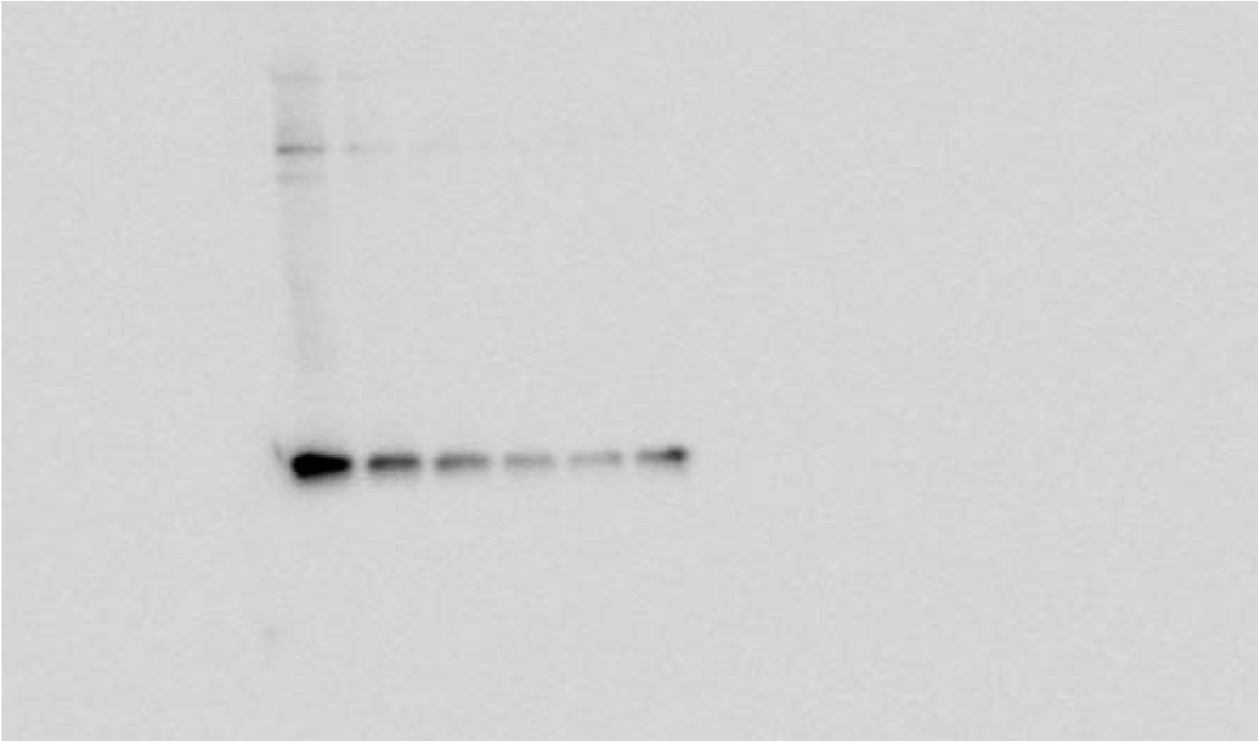

Figure S58 Western blot for phospho-S6RP (Ser235/236) of 769P cell line after BLU9931 treatment.
From left, Control, 1 nM, 10 nM, 100 nM, 1 μM, 10 μM with BLU9931 treatment concentrations.

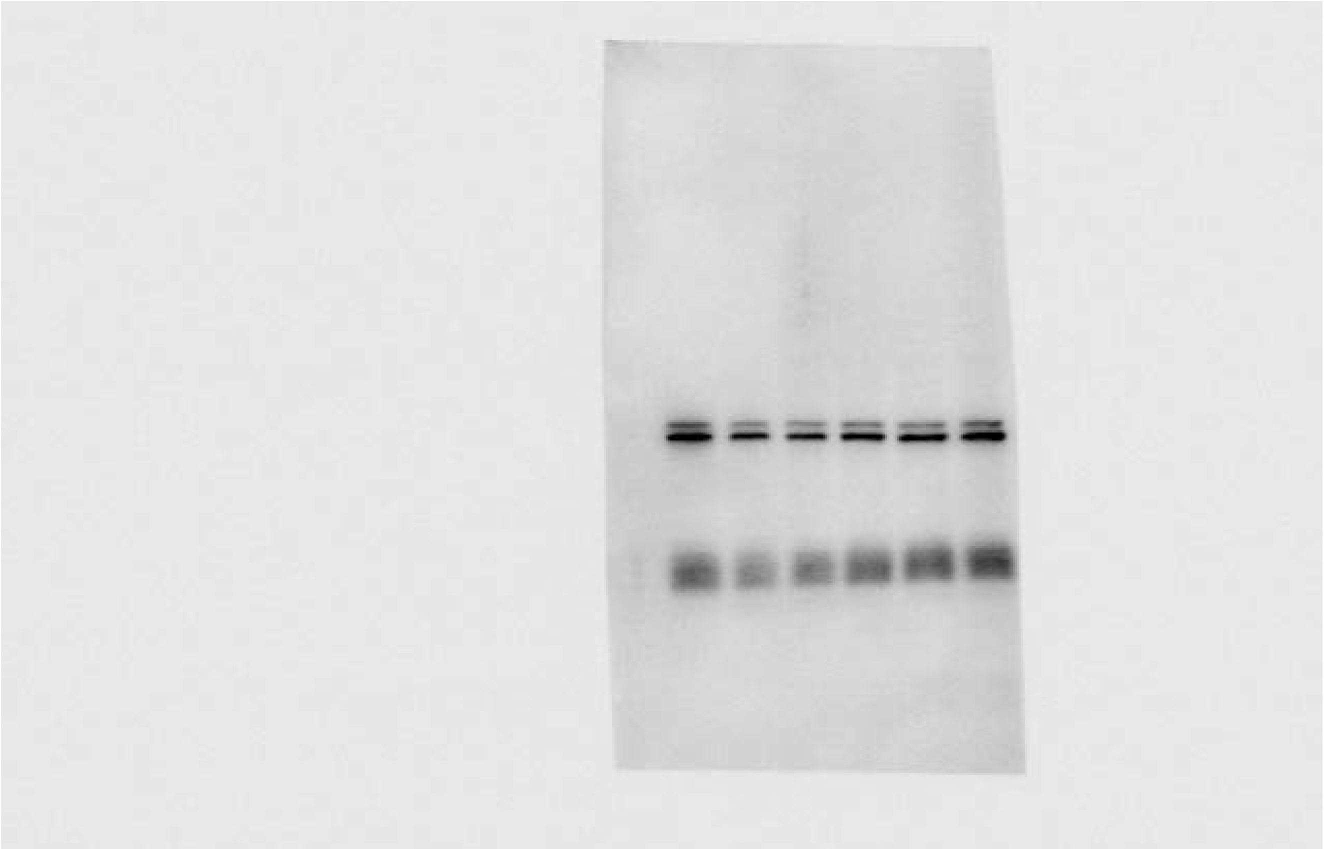

Figure S59 Western blot for ERK1/2 of 769P cell line after BLU9931 treatment.
From left, Control, 1 nM, 10 nM, 100 nM, 1 μM, 10 μM with BLU9931 treatment concentrations.

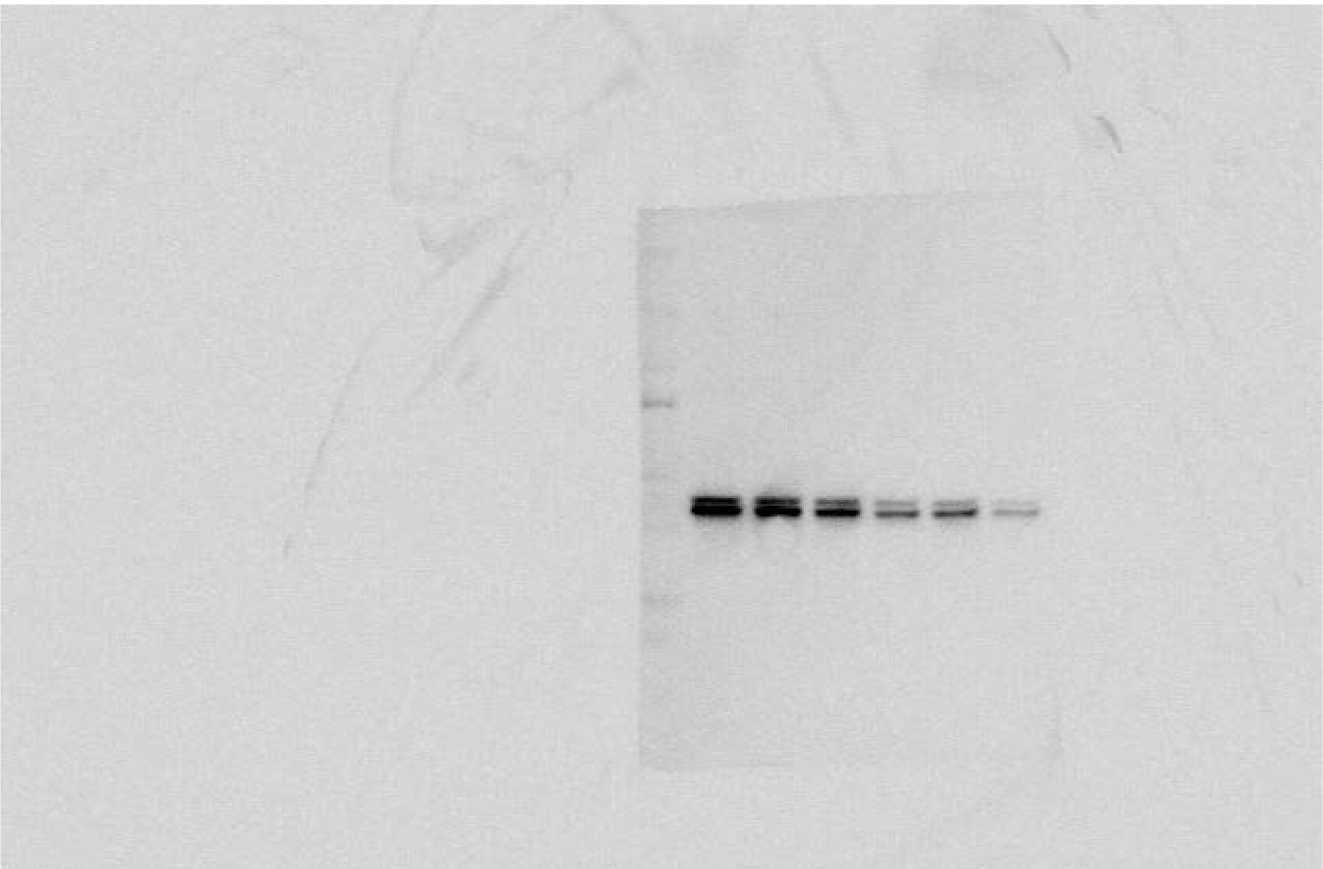

Figure S60 Western blot for phospho-ERK1/2 (Thr202/Tyr204) of 769P cell line after BLU9931 treatment. From left, Control, 1 nM, 10 nM, 100 nM, 1 μM, 10 μM with BLU9931 treatment concentrations.

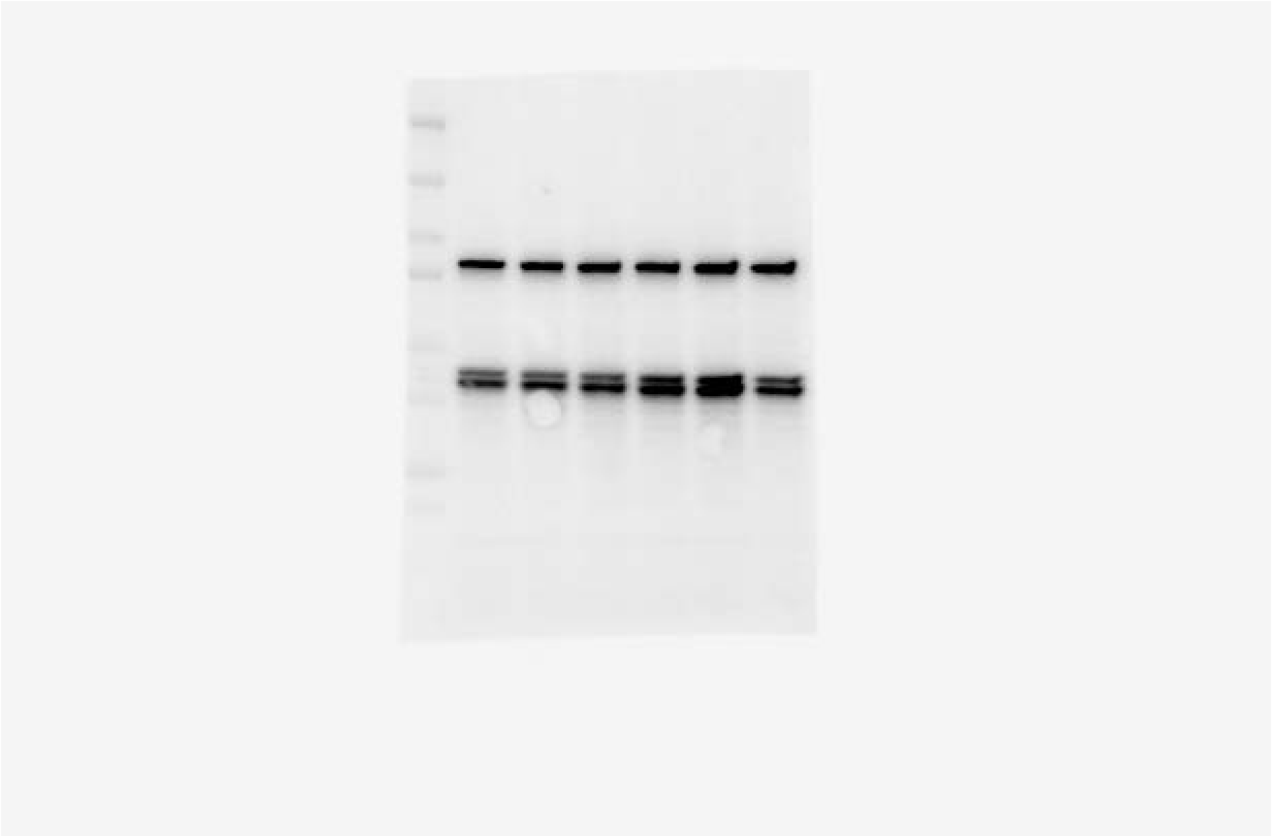

Figure S61 Western blot for STAT3 of A704 cell line after BLU9931 treatment.
From left, Control, 1 nM, 10 nM, 100 nM, 1 μM, 10 μM with BLU9931 treatment concentrations.

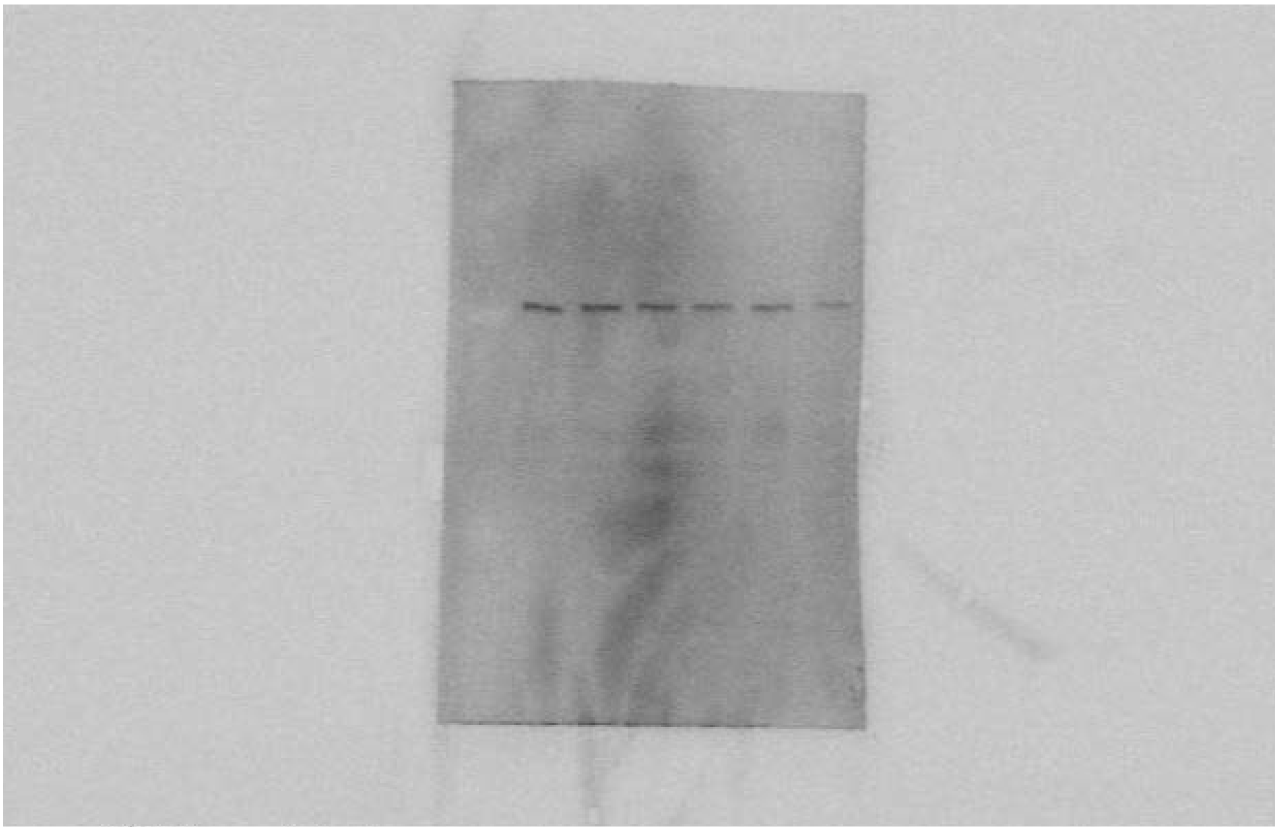

Figure S62 Western blot for phospho-STAT3 (Tyr705) of A704 cell line after BLU9931 treatment.
From left, Control, 1 nM, 10 nM, 100 nM, 1 μM, 10 μM with BLU9931 treatment concentrations.
